# A marine isotope stage 11 coastal Acheulian workshop with associated wood at Amanzi Springs Area 1, South Africa

**DOI:** 10.1371/journal.pone.0273714

**Published:** 2022-10-20

**Authors:** Andy I. R. Herries, Lee J. Arnold, Giovanni Boschian, Alexander F. Blackwood, Coen Wilson, Tom Mallett, Brian Armstrong, Martina Demuro, Fiona Petchey, Matthew Meredith-Williams, Paul Penzo-Kajewski, Matthew V. Caruana

**Affiliations:** 1 Palaeoscience, Dept. Archaeology and History, La Trobe University, Melbourne Campus, Bundoora, Victoria, Australia; 2 The Palaeo-Research Institute, University of Johannesburg, Auckland Park, South Africa; 3 Environment Institute and Institute for Photonics and Advanced Sensing (IPAS), Department of Earth Sciences, School of Physical Sciences, University of Adelaide, Adelaide, South Australia, Australia; 4 Dipartimento di Biologia, Università di Pisa. 1, Pisa, Italy; 5 Human Evolution Research Institute, University of Cape Town, Western Cape, South Africa; 6 Environmental Research Institute, University of Waikato, Hamilton, New Zealand; New York State Museum, UNITED STATES

## Abstract

Amanzi Springs is a series of inactive thermal springs located near Kariega in the Eastern Cape of South Africa. Excavations in the 1960s exposed rare, stratified Acheulian-bearing deposits that were not further investigated over the next 50 years. Reanalysis of the site and its legacy collection has led to a redefined stratigraphic context for the archaeology, a confirmed direct association between Acheulian artefacts and wood, as well as the first reliable age estimates for the site. Thermally transferred optically stimulated luminescence and post-infrared infrared stimulated luminescence dating indicates that the Acheulian deposits from the Amanzi Springs Area 1 spring eye formed during Marine Isotope Stage (MIS) 11 at ~ 404–390 ka. At this time, higher sea levels of ~13-14m would have placed Amanzi Springs around 7 km from a ria that would have formed along what is today the Swartkops River, and which likely led to spring reactivation. This makes the Amanzi Springs Area 1 assemblage an unusual occurrence of a verified late occurring, seaward, open-air Acheulian occupation. The Acheulian levels do not contain any Middle Stone Age (MSA) elements such as blades and points that have been documented in the interior of South Africa at this time. However, a small number of stone tools from the upper layers of the artefact zone, and originally thought of as intrusive, have been dated to ~190 ka, at the transition between MIS 7 to 6, and represent the first potential MSA identified at the site.

## Introduction

The Acheulian techno-complex, primarily defined by the production of Large Cutting Tools (LCTs) (i.e., handaxes and cleavers), appears to have its origins soon after 1.8 million years ago (Ma) in eastern Africa [1], and represents a long-lasting technological tradition persisting until a period between ~500–300 thousand years (ka) with the origins of the Middle Stone Age (MSA) [2]. While the Acheulian was initially considered an unchanging tradition with slow rates of cultural evolution, a great deal of temporal and regional variation is now evident [2–8]. After ~600–500 ka the occurrence of the first blades and points in combination with LCTs has produced a great deal of debate as to whether such assemblages should be seen as late occurring Acheulian, early MSA or as separate transitional industries such as the Fauresmith [2].

In an age when establishing the chronology of the Acheulian was impossible, Clark [9] simply separated Acheulian sites into Lower and Upper Acheulian occurrences, with distinct industries such as the Fauresmith and Sangoan being referred to as an evolved Acheulian tradition. At the time these were both considered to be Middle Pleistocene in age, with the Upper Acheulian extending into the Upper Pleistocene based on emerging radiocarbon (^14^C) ages, such as those from the site of Amanzi Springs in South Africa [10]. Some of these young ages were likely due to insufficient removal of exogenous carbon contaminants prior to the development of stringent ^14^C pretreatment procedures but this has not been tested at Amanzi springs till now. With the advent of radiometric and other dating techniques (e.g. palaeomagnetism) the beginning of the Acheulian has also changed and been pushed much further back in time and so sites that may once have been considered as ‘Early’ Acheulian may now represent later phases from a chronological perspective.

Kuman [11] defines Early (1.8–1.0 Ma), Middle (1.0–0.6 Ma) and Later (post 600 ka) Acheulian assemblages in part based on the notion that LCT morphologies become increasingly standardized through time. However, the size and shape of LCTs, as well as their degree of shaping do not always correlate with the expected age of a site and so ages defined entirely by such typological approaches are often incorrect [6, 12–20]. Moreover, other researchers prefer to define this post 600 ka period as transitional or early MSA, focusing on the first occurrence of new MSA technology such as blades and Levallois points in the record, rather than the continued occurrence of LCTs [2]. In such a scenario, using the term Middle Acheulian for the period between ~1.0–0.6 Ma makes little sense and these terminologies become hard to apply, increasingly confusing, and sometimes misleading. While some occurrences that have been defined as transitional/Fauresmith likely represent mixing and reworking of Acheulian artefacts into younger deposits [2], it is clear that at many sites in eastern and southern Africa that a transition occurs between the Acheulian and MSA with new MSA elements occurring alongside LCTs [21–23]. However, a great deal of variation occurs in the timing of such transitions across various parts of the world, with African style Acheulian without MSA elements occurring as late as ~190 ka in Arabia [3].

**Fig 1:**
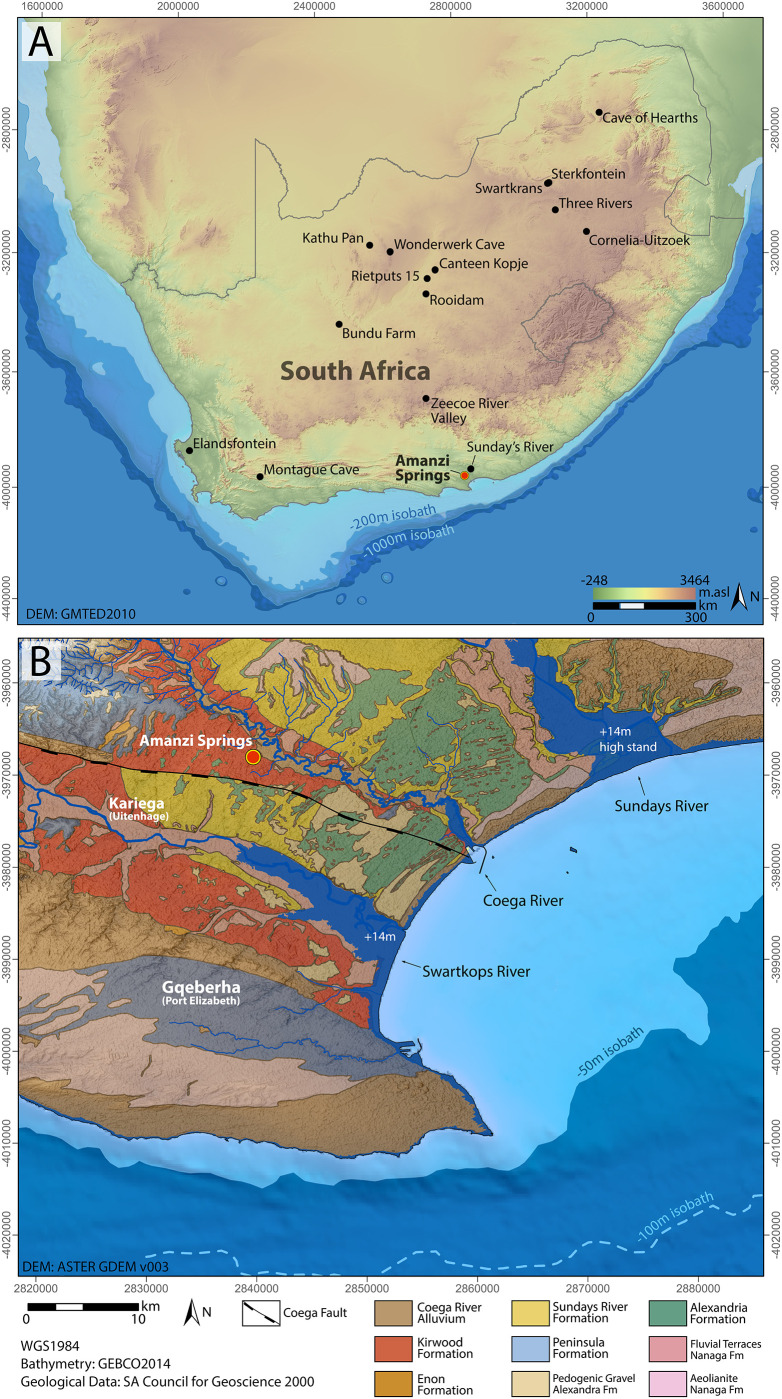
Location of Amanzi Springs. A) compared to other sites mentioned in the text; B) compared to the coastline today, during MIS 11 (+14m) and at the height of glacial periods (-100 to -120m). The Figure was created using open access data sources from [171–173].

Changes through the Acheulian are well documented in eastern Africa, where sequences can be dated using potassium/argon-argon dating [24]. In contrast, the dating of the south African record has been a lot more difficult due to a historic lack of methods to date the very different Acheulian contexts in South Africa, as well as a general lack of well stratified sites; especially in primary context [2, 25, 26]. The timing of, and changes within the Acheulian industry in South Africa is thus far from certain, as is geographic variation. The vast majority of well-studied and dated Acheulian sites occur on the highveld in northern central South Africa, with a few sites also occurring in the western part of the Western Cape below the Great Escarpment (Fig 1). In contrast, very little is known about the south-eastern part of the country, especially along the coastal region, other than from the site of Amanzi Springs, and more recently discovered sites along the Sunday’s River [10, 13, 14, 27, 28].

Kuman and colleagues [11, 25, 29] and Chazan et al [18] have argued that the Acheulian first appears contemporaneously with the eastern African record. In contrast, Herries and colleagues [2, 26, 30] have argued that there is no definitive evidence for the Acheulian prior to ~1.4 Ma. Much of the evidence for the earlier occurrence of the Acheulian comes from cosmogenic nuclide burial dating (Al/Be), such as at Wonderwerk Cave [18, 31]. However, recent uranium-lead (U-Pb) dating of stratified speleothem in the deposits instead date the Acheulian to either side of the Brunhes-Matuyama reversal at 780 ka [19]. Moreover, recent reanalysis of the Al/Be dating now suggests the Acheulian is not older than 1.2 Ma [20]. Other purported early Acheulian sites in the Cradle of Humankind, such as Swartkrans Member 2 and 3 and Sterkfontein Member 5, suffer from various issues of secondary context, being washed into the cave, small sample sizes, and often a lack of LCTs; Acheulian being suggested mainly on the basis of large flake sizes, as well as the occurrence of LCTs in the lime miners dumps [25]. Both also appear to date to less than ~1.4 Ma, [26, 30, 32–34]. Al/Be dates from sites along the Vaal River have very broad upper and lower age limits from published studies [2, 29] and at other river gravel sites the data sets and methods supporting the ages have not been published [2, 27, 28, 35]. Until the data underlying these cosmogenic dates is published the reliability of these age estimates should be treated with caution.

Others have divided the Acheulian into earliest, middle, and younger phases based on the presence or absence of prepared core technology and MSA tools such as blades and points [11, 36–38]. From about ~1.2–0.8 Ma onwards, various forms of prepared core technology appear to coincide with the production of large flake blanks for LCT manufacturing [16, 37]. In South Africa, Victoria West cores have been suggested to occur at Canteen Kopje after 1.20 ± 0.07 Ma based on a cosmogenic burial age from the base of the level B gravel layer [39, 40]. However, the data behind this cosmogenic burial age has yet to be published. The youngest Acheulian period containing LCTs with MSA-like or transitional looking elements has often been considered a transitional phase by some researchers, while others have been defined as Early MSA (EMSA), or have been specifically referred to the Fauresmith or Sangoan Industry [2, 41–44]. In South Africa, Mason [45] divided the Acheulian into Earlier (e.g., Sterkfontein, Vereeniging), Middle (Riverview Estates, Canteen Koppie Younger Gravel) and Later (Cave of Hearths Bed 1–3) periods based on typology and faunal dating, preferring not to distinguish Fauresmith assemblages, which he saw as simply later Acheulian variation. This was a view also followed by Sampson [46], although he retained the use of the term Sangoan and Final Acheulian for specific younger Acheulian material. While Inskeep [47] and Deacon [10] described Sangoan-like picks and heavy unstandardized bifaces from Amanzi Springs, they did not specifically assign it to the Sangoan. Sampson [46] similarly used the term Acheulian for Amanzi Springs and defined that no Sangoan sites existed in South Africa, using the term instead for what he considered definitive Sangoan sites such as Kalambo Falls. Re-excavation and dating of new excavations at Kalambo Falls by Barham et al. [42] identified Mode 3 technology sometime between 500 and 300 ka but they note that no further distinction (i.e. Sangoan or early Lupemban) can be made based on the small sample size. In such a model, Fauresmith and Sangoan assemblages are defined as Mode 3, defining them as early MSA, rather than later Acheulian (Mode 2) [42]. Fauresmith sites are thought to represent more open arid settings, while Sangoan more closed wooded or forested settings [2].

Regardless of the terms for the assemblages, recent dating suggests that Mode 3 technologies first emerge around 500 ka, with LCTs still prevalent [21, 22]. In eastern Africa, Middle Stone Age elements such as blades are seen prior to ~509 ka, with points occurring soon afterwards [23]. A similar trend is suggested in the interior highveld of South Africa at Kathu Pan, where points and blades appear to occur by at least 417 ka [22]. This age is only based on single electron spin resonance (542 ± 140 ka; ESR) and optically stimulated luminescence (OSL; 464 ± 47 ka) ages for slightly different units [22]. Overlying MSA layers have been dated to 291 ± 45 ka [22]. Dating the Acheulian and early Middle Stone Age in South Africa has been hampered by a lack of well-stratified sites, especially ones that are in primary context, as well as small assemblage size and a lack of methods to date this time period [2]. This lack of secure ages from southern African terminal Acheulian/early MSA sites complicates temporal divisions, and the timing of these phases remains ill-defined [2]. To advance the debate about the timing of the Acheulean and its transition to the MSA in South Africa it is thus critical to find new stratified and datable sites, as well as to apply new dating methods to sites that have not been studied for a long time. Only then will regional variance in the Acheulian and MSA be established.

**Fig 2:**
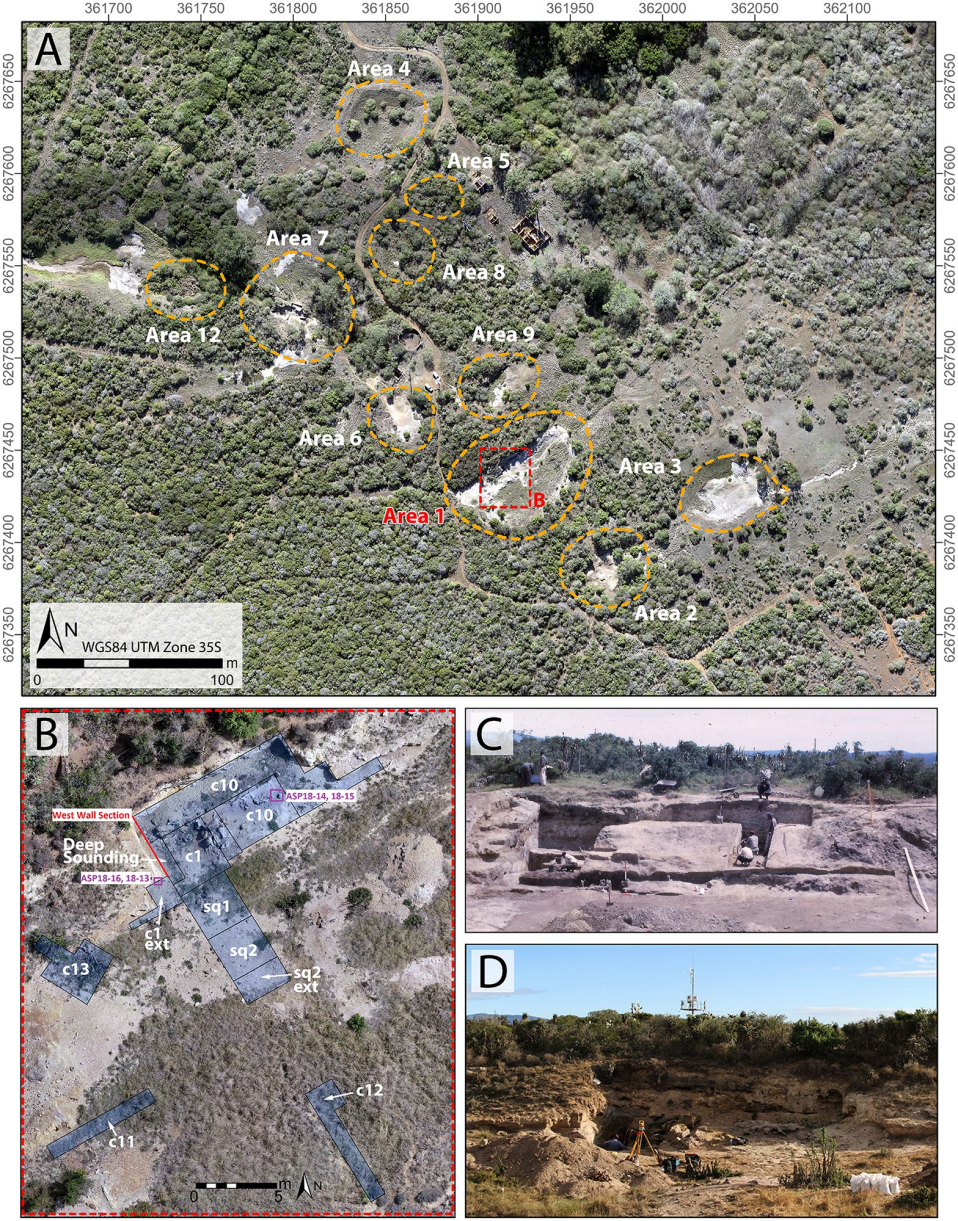
The Amanzi Springs site. A. Drone survey of Amanzi Springs showing the location of the various spring eyes, including Area 1; B. The Area 1 excavations of Deacon [10] showing the main excavation cuttings and squares, as well as luminescence dating locations and the location of the West Wall section shown in Fig 3; C and D, The Area 1 Cutting 1 and 10 excavations in the 1960s (C) and 2019 (D).

**Fig 3:**
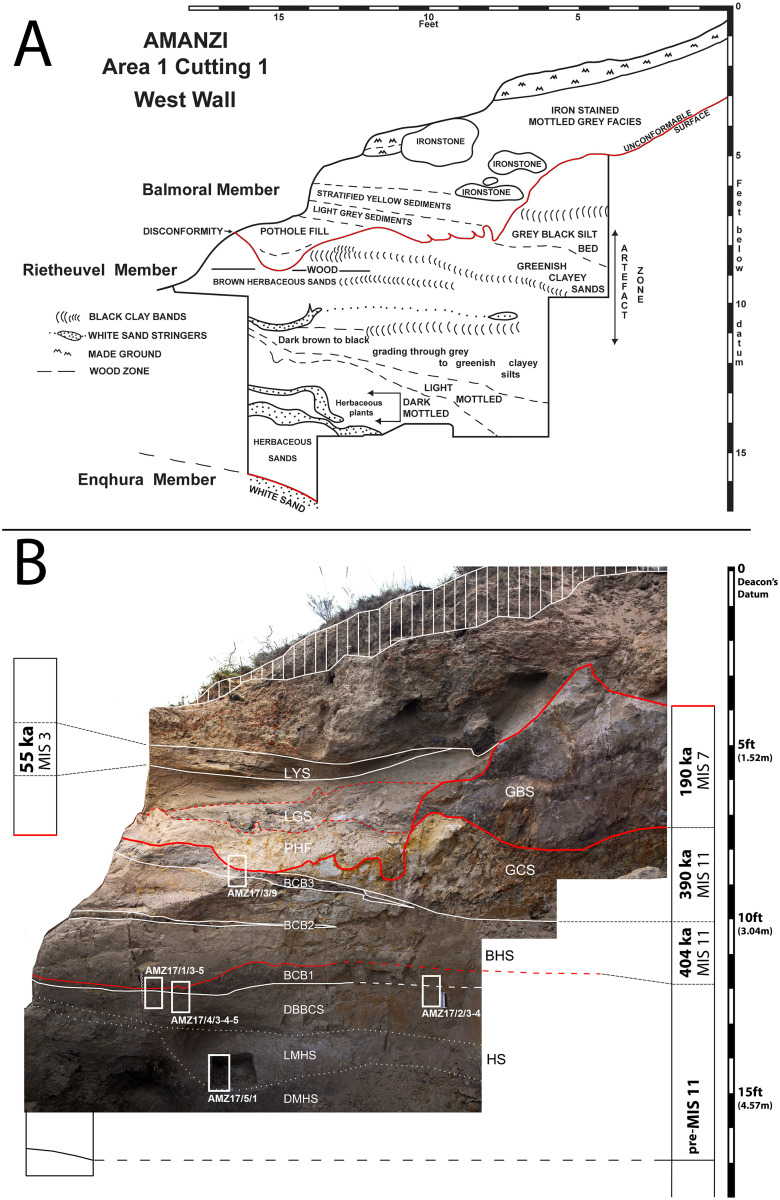
Amanzi Area 1 profile of the western section wall of Cutting 1. A) Redrawn section from Deacon [11]; B) Photomosaic of present-day situation, with indication of the stratigraphic units and members described in this paper, main disconformity (red line) and location of micromorphology samples (white rectangles).

A site that has the potential to resolve part of the Acheulian and early MSA record of South Africa is Amanzi Springs (Fig 1). Archaeological excavations in two of the eleven spring eyes (Fig 2) have yielded large Acheulian stone tool assemblages from stratified sediments [10], which is a unique geological setting for Earlier Stone Age localities in this region [2]. In fact, the majority of Acheulian sites in South Africa, including Canteen Kopje, Rietputs 15, Munro’s Site, Power’s Site, Sheppard Island and the Sunday’s River, are formed within alluvial deposits that are indicative of post-depositional alteration and complicate the contextual history of artefact assemblages [2, 27, 28, 40, 48, 49]. In the cave sites of the Cradle of Humankind the assemblages have often been washed in from the surrounding landscape [25]. As such, the stratified nature of the Amanzi Springs deposits (Fig 3) represents a rare opportunity to study Acheulian materials from a potentially near-primary sedimentary context.

While Deacon [10] recovered thousands of artefacts from his excavations, they were only partially analysed in his single volume published on the site. Deacon [10] attributed the assemblages to “a late or terminal Acheulian industry”, albeit noting that the materials appeared large and unstandardized [10] (p. 98). At this time, the Sangoan industry, represented by large, minimally-shaped core-axes and picks, was thought to proceed the Acheulian as a transitional phase before the onset of the MSA in southern Africa [46]. As such, Deacon may have thought that the unstandardized nature of the archaeology from Amanzi Spring reflected a gradation into Sangoan-like tool production, although most likely represented a later Acheulian assemblage given the frequency of LCTs. Since Deacon’s [11] work at Amanzi Springs it has remained virtually unstudied, with its collections solely used for occasional comparative purposes [e.g., 50, 51]. Recent work by two of us (MVC, AIRH) on the LCTs from the Area 1 museum collections has suggested that the reason for the unstandardized, and thick nature of LCTs is due to the fact that they represented various stages of failed production [13] and that Area 1 represents a raw material procurement and LCT production site [14].

In 2015, the Amanzi Springs Archaeological Project was formed to reassess the site and develop a chronological framework to contextualize its Acheulian assemblages. This has been operationalized through combined single-grain thermally transferred optically stimulated luminescence (TT-OSL), single-grain OSL and multiple-grain post-infrared infrared stimulated luminescence (pIR-IRSL) dating, palaeomagnetism, ground penetrating radar, micromorphological examination of the stratigraphic sequences, and an analysis of the complete archaeological collections previously excavated by Deacon. Here we present results of this research for Area 1, which includes an age for the Acheulian-bearing sediments, a micromorphological assessment of the stratigraphy and preliminary results of the archaeological analysis. The original goals were to: 1) determine the mineralogical composition of the sediments, to ascertain whether luminescence age determination would be possible; 2) explore the nature, origin and preservation of the organic matter, which gives some sediments a characteristic dark grey or black colour; 3) verify the origin of the reworked sediment aggregates that can be observed within some lithologic units; 4) observe the characteristics of the discontinuities and determine if they represent hiatuses in deposition or erosion processes; and 5) reconstruct depositional and post-depositional processes. Sediment samples were taken for preliminary TT-OSL, pIR-IRSL, OSL and palaeomagnetic analysis to test whether the deposits were suitable for those methods and could be dated. We constrain the deposition of the wood layer associated with Acheulian from Area 1 and propose a new stratigraphic nomenclature for the corresponding sedimentary layers. Lastly, we provide a preliminary description of the wider lithic assemblage and insights into the nature of this late occurring Acheulian occupation at Amanzi Springs, one that overlaps in time with sites in the interior that already contain Mode 3 technology.

## The Amanzi Springs site

Amanzi Springs is located approximately 10 km east of Kariega (formerly Uitenhage) and 25 km northeast of Gqeberha (formerly Port Elizabeth) in the Eastern Cape of South Africa (Fig 1). The site consists of 11 identified spring eyes occurring on the north side of a low hill (187m amsl), capped by silcrete, and is situated on a low plateau stretching NW-SW, delimited by the parallel valleys of Coega and Swartkops River, respectively (Figs 1 and 2). The Coega River lies ~1.5 km to the north and east of the site, the Swartkops ~7.5 km to the south and west. The bedrock of the plateau is ascribed to the Late Jurassic-Early Cretaceous Uitenhage Group, including light grey mudstones of the Kirkwood Formation that outcrop in the site area, as well as similar sediments of the younger Sundays River Formation [52, 53]. Older remnants of Enon Formation conglomerate also outcrop to the NW of Kariega [54]. These formations are paraconformably overlain by sediments of the Alexandria Fm. (Algoa Group), including conglomerates overlain by poorly cemented sandstones. Although the Alexandria Fm. does not occur in the vicinity of Amanzi Springs because it was completely eroded, some remains can be observed a few kilometres to the south of the site, whereas extensive outcrops are preserved 5–10 km to the north where it is overlain by wide patches of dunes ascribed to the Nanaga Fm. (Early Miocene-Early Quaternary; Algoa Group) [55] (Fig 1). To the west are extensive outcrops of Peninsula Formation Sandstone (aka Table Mountain Sandstone [TMS]), a quartzitic sandstone (Fig 1).

Amanzi Springs was first explored by Ray Inskeep [47] in 1963 who excavated the upper layers of Cutting 1 in Area 1 (Figs 2 and 3). Hilary Deacon [10] continued excavations from 1964 to 1965 as part of his Masters thesis. Deacon excavated a second spring, Area 2, as well as expanding (Cutting 10) and deepening Inskeep’s excavations (Cutting 1 Extension and Deep Sounding) into the northern rim of the Area 1 Spring depression, and excavating the central part of the spring (Squares 1 and 2) (Fig 2). In Area 1, Deacon [10] exposed a layered stratigraphic sequence (Fig 3), which he split into three Members (Enqhura, Rietheuvel, Balmoral from bottom to top), also described by Butzer [56], who correlated them across both the Area 1 and 2 springs. Deacon did not excavate into the ‘white sand’ (i.e., the lowest observed horizon) of the Enqhura Member in Cutting 1 or 10, just to its interface with the overlying Rietheuvel Member (Fig 3A). He excavated into the Enqhura Member in the centre of the spring eye in Square 1 and 2, where he noted that archaeological remains occur at its interface with the overlying units (Brown Herbaceous Sands of the Rietheuvel Member in Square 1 and modern fill in Square 2; [10]) (Fig 2). Deacon [10] states that excavation was limited due to the unconsolidated nature of the white sands.

The White Sands of the Enqhura Member are overlain by supposedly archaeologically sterile deposits at the base of the Rietheuvel Member (DMHS, LMHS, DBBCS and BCB1 in Fig 3). The nature of these units is variable with marked grading south to north across the West Wall section (Fig 3 [10; Fig 7]). The main Acheulian artefact bearing layers occurs between ~2.3 m (7.6 ft.) and 3.3 m (11 ft.) below Deacon’s datum (now gone) within Brown Herbaceous Sands (BHS; southern facies) and Greenish Clayey Sands (GCS; northern facies; also called ‘Yellow Clayey Silts’ in Deacon’s unpublished notes and section drawings). This primarily outcropped in Deacon’s Cutting 1 and 10 excavations (Figs 2 and 3), with the BHS facies also found in Square 1 and 2 (Sq 1, 2; Fig 2). The BHS facies have also yielded remnants of wood and the stems of herbaceous plants [57]. Wood from Ray Inskeep’s excavations of the upper levels of BHS in Cutting 1 (Inskeep’s 1a level Fig 3) [47] was ^14^C dated to >47,000 BP (GrN-4407-4546 extract) and 60,600 ± 1,100 BP (GrN-4407-4546 enriched) [10, 58] indicating a minimum age for the deposits [58]. Radiocarbon dates undertaken by Deacon [11] from BHS in Square 1 gave an age of 32,900 ± 600 BP (SR-103) [10], which might suggest the occurrence of intrusive younger wood in this area (SR-103) as these units do contain Acheulian [11]. These facies are overlain by a Grey Black Silt (GBS) facies that contained few stone tools, but included what Deacon [10 p110] described as “typologically foreign elements”. The youngest Balmoral Member consists of a complex mottled ironstone facies and laminated sand facies (LYS in Fig 3) overlying an artefact bearing ‘Pothole Fill’ (PHF), which lies over a distinct unconformable surface (Deacon’s Disconformity) which cuts the deposits of the underlying Rietheuvel Member (Fig 3). Deacon [10] notes the occurrence of Acheulian artefacts in his ‘Sample 4’ assemblage from the PHF, but concludes this sample is undiagnostic because it is likely derived and mixed. A piece of wood (shrunk within an isolated cavity) from the Balmoral Member (1ft above the disconformity at the front of Cutting 10 associated with the laminated sediments) was ^14^C dated to 31,000 ± 1200 BP (I-2241) [10], similar to the age from Square 1. Part of the new work at Amanzi is to re-evaluate the context of these old dates and the wood suggested to be associated with the Acheulian, as well as provide new dates for the site.

## Samples and methods

### Sampling

’All necessary permits were obtained for the described study, which complied with all relevant regulations.’ Excavations and sampling at the site was granted by the landowner and was carried out through permit number 2/2/AMP-PERMIT/16/09/110 issued by the Eastern Cape Provincial Heritage Authority (ECPHRA). Study of the archaeological collections excavated by Deacon [10] were granted by the Albany Museum, Makhanda that house the collection.

**Fig 4:**
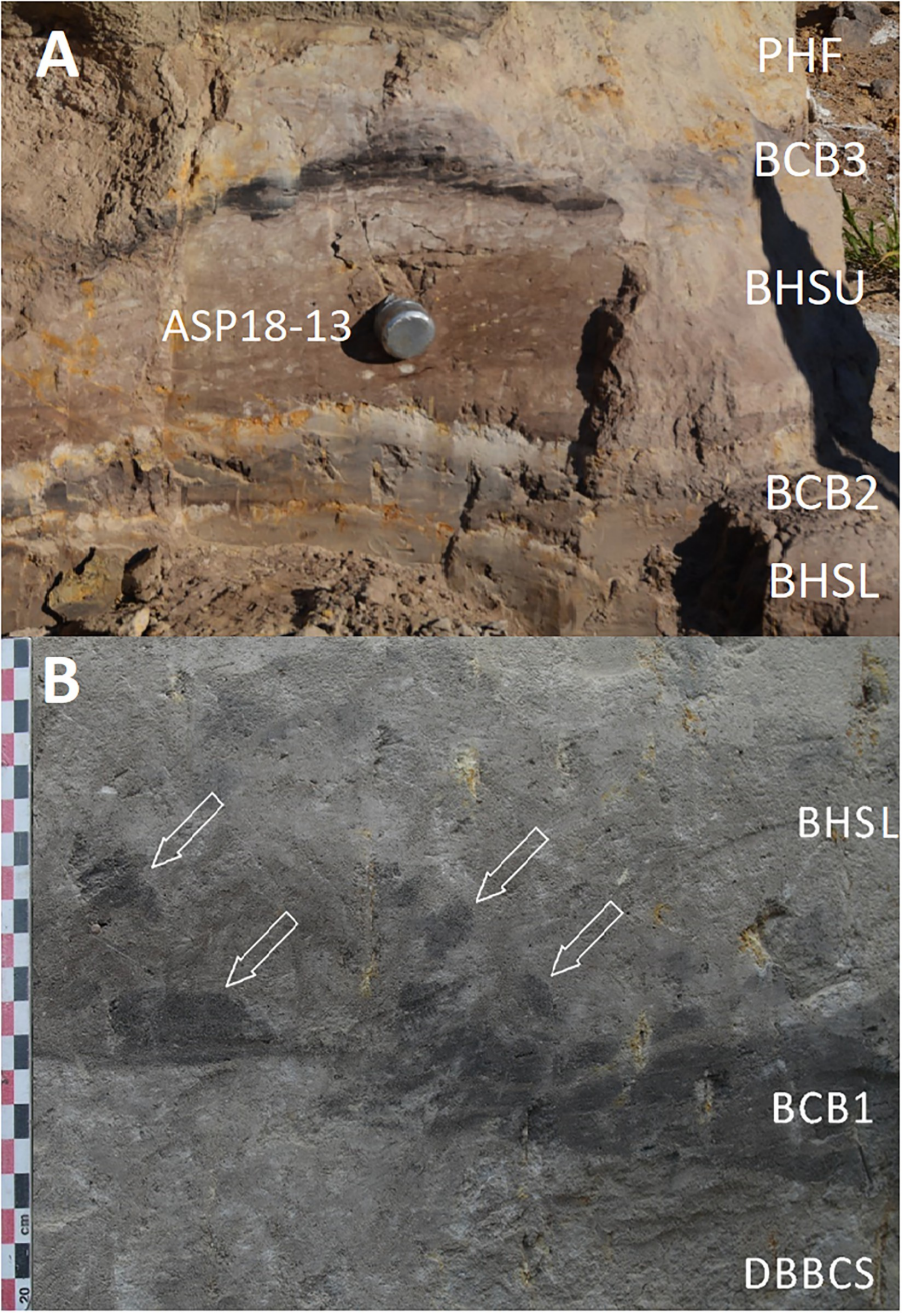
A. Location of luminescence dating sample ASP18-13 in the BHSU of Cutting 1 Extension B. Close-up of the boundaries between DBBCS, (BCB), and BH). BCB1 tapers leftwards and its top is evidently shaped by an erosion feature. Reworked clods of BCB1 (arrows) are embedded in BHSL.

Samples for micromorphology were taken from the West Wall section of Deacon’s (1970) Cutting 1 Deep Sounding (Fig 3). Luminescence and palaeomagnetic samples from the GBS and GCS facies were taken from the central area of Cutting 10 where Deacon stopped his excavations at the disconformity, thus exposing GBS below. Further luminescence samples were taken from the Cutting 1 Extension section Wall (Fig 4A). We excavated a small 1 x 1m trench through the edge of this exposure to reveal the GBS and GCS layers (Fig 2). This excavation confirmed a sharp unconformable contact between these two units (Fig 4B). Radiocarbon dating samples (AMZ1-259; AMZ1-264) of wood and charcoal were also taken from Acheulian bearing levels of BHS in a test excavation undertaken adjacent to Deacon’s Square 2 as this is where an age of ~38,100 BP was yielded from a wood sample by Deacon [10].

#### Radiocarbon

One sample of wood and another of charcoal were recovered from the BHS in our Square 2 Extension test trench in direct association with Acheulian stone tools (Fig 19B). AMS ^14^C analyses were performed at the University of Waikato radiocarbon laboratory. The ^14^C samples were prepared using standard acid-base-acid pre-treatment with isotopic fractionation corrected for using measured δ^13^C values [59]. Percent carbon (%C) recorded from combustion was used to assess sample quality, which should yield carbon percentages of >50%. This parameter has been found to be useful for assessing the preservation state of charcoal close to the limit of the radiocarbon technique [59] (S1.5 Table in S1 File).

#### Luminescence

To assess the age of the archaeological layers and associated wood, three luminescence dating samples were taken from the archaeological bearing deposits of Deacon’s [10] Rietheuvel Member and one from the Balmoral Member (see Fig 2B for sample locations). Samples from GBS (sample ASP18-14) and the uppermost Acheulian-bearing GCS (sample ASP18-15) layers were taken in the central area of Cutting 10, and from the lowermost Acheulian-bearing BHS horizon (sample ASP18-13) in Cutting 1 extension (BHSL; Fig 4A). An additional sample was taken from the LYS layer (Fig 3) of the Balmoral Member (sample ASP18-16) in the Cutting 1 Extension (Fig 2), the same layer that yielded a ^14^C age of ~32,900 BP by Deacon [10]. The luminescence samples were taken from cleaned sedimentary exposures using PVC or metal tubes, with additional bulk sediment collected from the surrounding few cm of each tube for beta dose rate determination and water content analysis. Given the expected antiquity of Area 1 (as indicated by the presence of LCTs), we have utilised two semi-independent, extended-range luminescence dating techniques (single-grain quartz TT-OSL dating and multiple-grain K-feldspar pIR-IRSL dating; e.g., [60, 61]), which exhibit considerably higher dose saturation properties than conventional quartz OSL dating [62] and offer the potential to establish finite depositional chronologies over Middle Pleistocene timescales [63–68]. Traditional single-grain quartz OSL dating has additionally been applied to sample ASP18-16 as a finite, albeit tentative, ^14^C age had previously been obtained on the Balmoral Member [10], suggesting that the LYS deposits could be significantly younger than the underlying Rietheuvel Member. The combined TT-OSL, pIR-IRSL and OSL dating approach employed in this study enabled us to cross-check the reliability of the luminescence chronologies for Area 1 using multiple mineral fractions (quartz versus K-feldspar) and at different scales of D_e_ analyses (multiple-grain versus single-grain).

K-feldspar (90–125 μm diameter) and quartz (212–250 μm diameter) grains were extracted from the un-illuminated centres of the tubes under safe light (dim red LED) conditions at the University of Adelaide’s Prescott Environmental Luminescence Laboratory, and prepared for burial dose estimation using standard procedures [69]. The latter included etching by hydrofluoric (HF) acid to remove the alpha-irradiated external layers (10% HF digestion for 10 min to etch the K-feldspar fractions; 48% HF digestion for 40 min to etch the quartz fractions) [70, 71] and subsequent washing in 30% hydrochloric acid to remove any precipitated fluorides. K-feldspar pIR-IRSL, quartz TT-OSL and quartz OSL equivalent dose (D_e_) measurements were made using the experimental apparatus, single-aliquot regenerative-dose (SAR) procedures, and quality assurance criteria described previously by [64, 67, 72] (see S1 File for full details). Multiple-grain K-feldspar dose-response curves were constructed from the first 10 s of each pIR-IRSL signal after subtracting a mean background count from the last 10 s of stimulation. Single-grain OSL and TT-OSL dose-response curves were determined using the first 0.08 s of each green laser stimulation after subtracting a mean background count obtained from the last 0.25 s of the signal. Multiple-grain pIR-IRSL D_e_ measurements were made on 10–15 aliquots of K-feldspars per sample (each aliquot containing ~400 grains), while single-grain TT-OSL and OSL D_e_ measurements were made on 500–1000 individual quartz grains per sample (Table 1). For TT-OSL and OSL D_e_ measurements, 212–250 μm quartz grains were measured in aluminium discs drilled with an array of 300 × 300 μm holes to ensure true single-grain resolution [73].

**Table 1:** Dose rate data, equivalent doses and luminescence ages.

Sample	Layer ^a^	Grain size (μm)	Water content (%) ^b^	Environmental dose rate (Gy/ka)					Equivalent dose (D_e_) data					
				Beta dose rate ^c^	Gamma dose rate ^d^	Cosmic dose rate ^e^	Internal dose rate ^f, g^	Total dose rate ^h^	D_e_ type ^i^	No. of grains or aliquots ^j^	Overdis-persion (%) ^k^	Age model ^l^	D_e_ (Gy) ^h^	Age (ka) ^h, m^
ASP18-16	LYS	212–250	5 / 18	0.56 ± 0.03	0.61 ± 0.02	0.12 ± 0.01	0.03 ± 0.01	1.32 ± 0.08	SG OSL	106 / 500	51 ± 4	CAM	114 ± 6	86.0 ± 7.3
												MAM-3	75 ± 9	56.3 ± 8.0
		212–250		0.56 ± 0.03	0.61 ± 0.02	0.12 ± 0.01	0.03 ± 0.01	1.32 ± 0.08	SG TT-OSL	101 / 1000	63 ± 6	CAM	150 ± 11	112.9 ± 11.0
												MAM-3	81 ± 13	61.2 ± 10.4
		90–125		0.60 ± 0.03	0.61 ± 0.02	0.12 ± 0.01	0.49 ± 0.04	1.82 ± 0.09	MG pIR-IRSL	15 / 15	35 ± 6	CAM	158 ± 14	86.8 ± 9.2
												MAM-3	99 ± 7	54.3 ± 4.8
ASP18-14	GBS	212–250	11 / 22	0.83 ± 0.04	0.59 ± 0.02	0.11 ± 0.01	0.03 ± 0.01	1.56 ± 0.10	SG TT-OSL	136 / 900	39 ± 4	CAM	325 ± 14	208.8 ± 16.4
		90–125		0.89 ± 0.05	0.59 ± 0.02	0.11 ± 0.01	0.49 ± 0.04	2.07 ± 0.11	MG pIR-IRSL	12 / 12	14 ± 3	CAM	362 ± 15	174.9 ± 12.0
ASP18-15	GCS	212–250	21 / 27	0.80 ± 0.04	0.65 ± 0.02	0.10 ± 0.01	0.03 ± 0.01	1.58 ± 0.10	SG TT-OSL	95 / 1000	25 ± 4	CAM	608 ± 24	385.8 ± 28.9
		90–125		0.86 ± 0.04	0.65 ± 0.02	0.10 ± 0.01	0.49 ± 0.04	2.09 ± 0.11	MG pIR-IRSL	10 / 10	11 ± 2	CAM	814 ± 26	389.6 ± 24.5
ASP18-13	BHS	212–250	9 / 21	0.76 ± 0.04	0.58 ± 0.02	0.11 ± 0.01	0.03 ± 0.01	1.47 ± 0.09	SG TT-OSL	103 / 1000	35 ± 4	CAM	587 ± 26	398.7 ± 31.6
		90–125		0.81 ± 0.04	0.58 ± 0.02	0.11 ± 0.01	0.49 ± 0.04	1.98 ± 0.11	MG pIR-IRSL	10 / 10	5 ± 1	CAM	800 ± 14	403.9 ± 23.4

See main text for explanations for columns (c-g and k-m)a LYS = Laminated Yellow Sediments; GBS = Grey Black Silts; GCS = Greenish Clayey Sands; BHS = Brown Herbaceous Sands. b Present-day water content / long-term estimated water content, expressed as % of dry mass of mineral fraction, with an assigned relative uncertainty of ±5%. The long-term water content of these samples is taken as 70% of the saturated water content estimate (see S1 File for details).

h Mean ± total uncertainty (68% confidence interval), calculated as the quadratic sum of the random and systematic uncertainties.i SG OSL = single-grain optically stimulated luminescence; SG TT-OSL = single-grain thermally transferred OSL; MG pIR-IRSL = multiple-grain aliquot post-IR IRSL performed at 250°C. j Number of D_e_ measurements that passed the SAR rejection criteria and were used for D_e_ determination / total number of D_e_ values analysed.

Detailed sensitivity analyses were initially performed on related luminescence dating samples collected from another spring eye at the site, Area 7, to ascertain suitable SAR preheating and measurement conditions for the various sedimentary deposits preserved at Amanzi Springs. The optimum SAR conditions determined for Area 7, which are summarised in S1.1 Table in S1 File, were then cross-checked for the Area 1 sediments using a series of dose recovery tests performed on samples ASP18-13 and ASP18-16. The optimum multiple-grain K-feldspar SAR protocol from Area 7, which involves pIR-IR stimulation at 250 °C after a preheat of 280 °C for 60 s (pIR-IRSL_250_ signals) (S1.1 Table in S1 File), yielded a dose recovery ratio in agreement with unity at 1σ (1.02 ± 0.02) for sample ASP18-13, and is therefore considered suitable for dating the Area 1 deposits (see S1 File for details). The optimum single-grain quartz OSL SAR protocol from Area 7 includes a preheat of 240°C for 10 s prior to measurement of the natural (L_n_) and regenerative dose (L_x_), and a preheat of 200°C for 10 s prior to measurement of the test-dose (T_n_ and T_x_) OSL signals (S1.1 Table in S1 File). These preheating conditions yielded an accurate measured-to-given dose ratio of 1.00 ± 0.02, and an overdispersion value of 13 ± 2%, for a 100 Gy dose recovery test performed on individual grains of ASP18-16 from Area 1 (S1.1 Fig in S1 File). The single-grain TT-OSL SAR protocol (S1.1 Table in S1 File) makes use of a TT-OSL test dose to correct for sensitivity change, four preheats of 260 °C for 10 s in each SAR cycle, and two high temperature OSL treatments to prevent TT-OSL signal carry over from previous L_x_ and T_x_ measurement steps. A single-grain TT-OSL measured-to-given dose ratio of 0.98 ± 0.06 was obtained when applying this SAR procedure to sample ASP18-13 (see S1 File for details), supporting the suitability of the TT-OSL D_e_ measurement conditions for dating purposes. Anomalous fading assessments were performed on subsets of aliquots from each sample to investigate the potential for long-term athermal loss of K-feldspar pIR-IRSL_250_ signals over burial timescales. Anomalous fading measurements were made on aliquots that had previously been used to derive D_e_ values following the procedures of Auclair et al. [74], which involved undertaking repeated SAR L_x_/T_x_ measurements after different storage times of 0.2–30 h. Anomalous fading rates (*g*-value) normalised to 2 days were calculated as described in Huntley and Lamothe [75], and used to quantify the expected percentage of signal loss per decade of storage time.

Dose rate evaluations have been undertaken using a combination of *in situ* gamma-ray spectrometry and low-level beta counting of dried and homogenised, bulk sediments collected directly from the luminescence sampling positions (Table 1). Gamma dose rates were determined from *in situ* gamma spectrometry using the ‘windows’ method described in [76] and Duval and Arnold [77]. External beta dose rates were determined from dry and homogenised sediment sub-samples collected from each luminescence dating sample position using a Risø GM-25-2 beta counter [78] Cosmic-ray dose rate contributions have been calculated using the equations of Prescott and Hutton [79] after taking into consideration site altitude, geomagnetic latitude, and density, thickness and geometry of sediment overburden.

Radionuclide concentrations and specific activities have been converted to dose rates using the conversion factors given in Readhead [80] and Guérin et al. [81] (see Table 1 footnotes for details), making allowance for beta-dose attenuation [82, 83], minor internal alpha dose rate contributions [84], and long-term water content [85], see Table 1 and S1 File. Present-day water content / long-term estimated water content is expressed as % of dry mass of mineral fraction, with an assigned relative uncertainty of ±5%. The long-term water content of these samples as presented in Table 1(b) is taken as 70% of the saturated water content estimate (see S1 File for details). Beta dose rates in Table 1(c) were calculated using a Risø GM-25-5 low-level beta counter [78], after making allowance for beta dose attenuation due to grain-size effects and HF etching [82, 83]. Radionuclide concentrations and specific activities of beta counting standards have been converted to dose rates using the conversion factors given in Guérin et al. [81]. Gamma dose rates in Table 1(d) were calculated from in situ measurements made at each sample position with a NaI:Tl detector using the ‘energy windows’ method detailed in Arnold et al. [73] and Duval and Arnold [77]. Radionuclide concentrations and specific activities of gamma spectrometry calibration materials, and K, U, Th concentrations determined from the field gamma-ray spectra have been converted to dose rates using the conversion factors given in Guérin et al. [81]. Cosmic-ray dose rates in Table 1(e) were calculated according to Prescott and Hutton [79] and assigned a relative uncertainty of ± 10%. The assumed internal alpha + beta dose rate in Table 1(f) for quartz, with an assigned relative uncertainty of ±30%, is based on intrinsic ^238^U and ^232^Th contents published by Mejdahl [86], Bowler et al. [84], Jacobs et al. [87], Pawley et al. [88] and Lewis et al. [89], and an a-value of 0.04 ± 0.01 [90, 91]. Intrinsic radionuclide concentrations and specific activities have been converted to dose rates using the conversion factors given in Guérin et al. [81], making allowance for beta dose attenuation due to grain-size effects [82]. The assumed internal feldspar dose rate in Table 1(g) is based on assumed internal ^40^K and ^87^Rb concentrations of 12.5 ± 0.5% [92] and 400 ± 100 ppm [93], respectively, yielding an internal beta dose rate of 0.43 ± 0.03 Gy / ka for the 90–125 μm K-feldspar grains measured in this study. An additional internal alpha + beta dose rate of 0.06 ± 0.03 Gy / ka has been calculated for the K-feldspar fractions using assumed intrinsic ^238^U and ^232^Th concentrations of 0.15 ± 0.03 ppm and 0.35 ± 0.07 ppm, respectively (following [86, 94–96]), and an a-value of 0.09 ± 0.03 (following [90, 97–101]). Intrinsic radionuclide concentrations and specific activities have been converted to dose rates using the conversion factors given in Guérin et al. [81] and Readhead [80], making allowance for beta dose attenuation due to grain-size effects [80, 82]. The relative spread in the De dataset beyond that associated with the measurement uncertainties for individual De values in Table 1(k) were calculated using the central age model (CAM) of Galbraith et al. [102]. The age model used to calculate the sample-averaged De value for each sample in Table 1(l). MAM-3 = 3-parameter minimum age model; CAM = central age model [102]. Sample ASP18-16 exhibits complex De distribution characteristics and high overdispersion values for all three luminescence signals, indicative of enhanced extrinsic and/or intrinsic D¬e scatter (see main text for further discussion). We have therefore provided both the MAM-3 and CAM ages for this sample. The choice of whether to use the three or four parameter minimum age model (MAM-3 or MAM-4) for this sample has been made on statistical grounds using the maximum log likelihood score criterion outlined by Arnold et al. [103]. Single-grain OSL and TT-OSL minimum age model De estimates were calculated after adding, in quadrature, a relative error of 20% to each individual De measurement error to approximate the underlying dose overdispersion observed in the dose recovery tests for samples ASP18-13 and ASP18-16, and for an ‘ideal’ (well-bleached and unmixed) sedimentary sample from this site (ASP18-15). Multiple-grain pIR-IRSL minimum age model De estimates were calculated after adding, in quadrature, a relative error of 5% to each individual De measurement to approximate the underlying dose overdispersion observed in the dose recovery test for sample ASP18-13 and for an ‘ideal’ (well-bleached and unmixed) sedimentary sample from this site (ASP18-13). The total uncertainty in Table 1(m) includes a systematic component of ±2% associated with laboratory beta-source calibration.

#### Palaeomagnetism

Oriented block samples were taken from the GCS archaeological-bearing layers sampled for luminescence in the Rietheuvel Member using a Suunto magnetic compass and clinometer. These were subsequently transported and cut into standard palaeomagnetic 8ccm subsamples at The Australian Archaeomagnetism Laboratory (TAAL), La Trobe University, Australia. Subsamples were subject to a range of demagnetisation strategies at TAAL including thermal (TH) demagnetisation, alternating field (AF) demagnetisation, and a hybrid approach utilising low-field AF steps (up to 8–10 mT) prior to TH demagnetisation (AF/TH). TH demagnetisation was undertaken using a Magnetic Measurements MMTD80 Thermal Demagnetiser in a zero-field cage and AF demagnetisation was undertaken using an AGICO LDA5 Alternating Field Demagnetizer. All remanence measurements were undertaken using an AGICO JR-6 Spinner Magnetometer (2.4 μAm sensitivity). The characteristic remanent magnetisation (ChRM) for each subsample was assigned using principle component analysis [104] in PuffinPlot 1.03 [105], isolating the most stable magnetic component on trend towards the origin of vector diagrams. ChRMs were composed of >5 consecutive demagnetisation data points and calculated unanchored to avoid any potential distortion to directional data [106]. Acceptance criteria for ChRM directions were principle components with a median angle of deflection (MAD) of <10°. Declination values were corrected for local secular variation values at Amanzi Springs (*D*: 333.0, *I*: -64.4) using the 12th-generation International Geomagnetic Reference Field (http://geomag.bgs.ac.uk/data_service/models_compass/igrf_form.shtml). Final mean directions for each block and Fisher statistics [107] were calculated in PuffinPlot [105]. Additional rock magnetic and X-ray absorption near edge structure (XANES) analysis were also performed to help understand the magnetic mineralogy of the samples (see S1 File).

#### Micromorphology

Five undisturbed sediment monoliths, approximately 12x7x3 cm were detached from the West Wall profile of Deacon’s [10] Cutting 1 Deep Sounding in Area 1 (Fig 3). Sampling loci were selected prevalently within dark layers and/or at boundaries with other layers and are shown in Fig 3. These monoliths were air-dried in ventilated oven at 35 °C for one week and subsequently impregnated with low-concentration acetone-dissolved polyester resin under moderate vacuum. Polymerisation was fostered by heating at 40 °C for about 30 days, then the resin was left to harden for 3 months. The impregnated monoliths were cut by diamond saw, glued on 90x60 mm microscope slides, reduced to about 0.5 mm again by diamond saw, and finally ground to 30 μm by corundum and aluminium oxide powder using petroleum as coolant. The slides were finally covered by standard microscopy protection glass. The thin sections were examined using a Zeiss Axio Scope.A1 standard petrographic microscope under 2.5x, 10x, 20x and 50x magnification. Thin section descriptions follow the standard criteria proposed by Stoops [108]. Thin sections were also directly scanned at 2400 DPI resolution by an Epson Perfection V550 Photo Scanner. Microphotographs were shot using a 7 Mpx Panasonic-Lumix DMC-L1 camera attached to the microscope. Tiff-format images were processed by decorrelation stretching, a technique enhancing the colour separation of images with significant band-to-band correlation, using MathWorks Matlab^®^ R2019a software. This technique discriminates the black colour of the amorphous organic matter (AOM) from the very dark brown hues of Fe- and Mn-oxides. The enhanced images were segmented by colour thresholding using Fiji-ImageJ software [109] in order to extract and quantify the areas corresponding to aggregates of amorphous organic matter.

#### Archaeological analysis

The lithic collections from Area 1 are housed at the Albany Museum (Makhanda, formerly Grahamstown, Eastern Cape, South Africa), which were measured and described to characterize typo-technological trends. All artefacts were measured using digital callipers (to the nearest 0.01 mm) and a digital scale for weight (to the nearest 0.01 g) and Kruskal-Wallis tests were used to statistically compare morphometric variables. Given the influence of water underlying the sedimentation at Area 1, it is important to investigate the influence of post-depositional processes on assemblage accumulation. For this purpose, we have used the approach of Schick [110], who examined artefact size curves via maximal length to understand winnowing effects that indicate primary versus secondary depositional contexts. Weathering patterns including fluvial abrasion, patination, chemical degradation, and the degree of rounding on both tool edges and flake scar ridges were also recorded to understand the degree of post-depositional disturbance. Patterns of raw material selection were investigated through inspection of the non-cortical surfaces with a hand lens (x10 magnification) to examine lithological properties of raw materials, including groundmass texture, grain sorting and the presence of inclusions [111, 112]. Cortical surfaces on cores and LCTs that provided information on their original blank shape were also assessed to investigate potential trends in the selection of raw material package morphologies.

Cores were characterized through a diacritical analysis, which examined the technical actions underlying core reduction, following methods outlined in Diez-Martín et al. [15] and Sánchez-Yustos et al. [113]. Cores were first categorized into reduction systems according to the number of knapped faces, including unifacial, bifacial and multifacial groups. These systems were further differentiated by knapping techniques, which assessed the orientation of flake scars and rotation patterns, as well as the angle of percussion. Flakes were analyzed in terms of their typology and distinguished according to their affiliation to small debitage and LCT production (*éclats de taille de biface*) chains. The Technological Flake Categories system developed by Toth [114], dorsal scar frequencies and directionality, as well as platform types were examined to investigate reduction sequences [115]. Further, the ratio of debitage elements was compared to average number of flake scars on small debitage cores to understand if the debitage series reflects a full range of knapping activities on site [116].

Large cutting tools were inspected through descriptive and metric variables similar to those recently reported in the WEAP method, including blank type (large flake or cobble), number of faces, cortex percentage and location, plan view and profile shape, total number of flake scars, number of primary shaping, and the number of thinning and retouch scars [117]. Handaxes from Area 1 were compared to examples from Rietputs 15 (n = 57; ~1.3 Ma) [29, 35] and Cave of Hearths (n = 64; <780 ka) [45, 118], representing earlier and later Acheulian assemblages, respectively [see 16]. Data were taken from Li et al.’s [16] published dataset of handaxe metrics to understand how the Amanzi Springs sample compares to these assemblages both morphometrically and in terms of reduction intensity. The L/W (elongation) and W/Th (refinement) indices described by Roe [119] were compared, as well as the scar density index outlined by Clarkson [120]. Li et al.’s [16] version of the SDI, using volume rather than surface area is used here to better account for the three-dimensional nature of these tools.

Utilized tools were classified according to tool types and usewear patterns recently reported by Goren-Inbar et al. [121], Alperson-Afil and Goren-Inbar [122] and Hardy et al. [123], which included core tools, massive scrapers, and flake tool categories. Retouched tools are a small component of the archaeological record from Amanzi Springs and were identified through microscopic inspection at x10 magnification. To describe retouch patterns the retouch type, location, invasiveness and edge delineation were recorded [124].

## Results

### Macrostratigraphy

Deacon did not infill the Cutting 1 and 10 excavations in Area 1 and they have collapsed significantly over the last 50 years. To expose Deacon’s [10] Cutting 1 West Wall witness section for geological sampling and stratigraphic analysis, we re-excavated Deacon’s Deep Sounding below the level of PHF that was exposed in the excavation wall (Fig 3). The deep sounding had infilled from the collapse of the west wall, particularly its northern section, which unfortunately also caused the loss of Deacon’s datum point. However, during our excavations we were able to relocate some of Deacon’s excavation nails still in situ, including the nails in Plate 3 of Deacon’s [10] (p140) monograph. This allowed us to reorient our work to Deacon’s levels. We further oriented our grid with a drone survey for the whole spring complex (Fig 2A). While sediments had collapsed from the West Wall, the stratigraphy identified by Deacon [10] was still easily observable (Fig 3). The excavation profile exposed on the West Wall of Cutting 1 and Deacon’s Deep Sounding puts into light several subhorizontal or gently dipping layers- and lens-shaped lithologic units, mostly consisting of sand to sandy loam sediments (Fig 3). The lowermost unit exposed by Deacon [10], i.e., the ‘White Sands’ of the Enqhura Member does not occur in the West Wall section as Deacon’s Deep Sounding only reached the very top of it in a small area at its very southern end. Above the Enqhura Member, the sequence includes the following units, from the bottom upwards within Deacon’s Members framework, defined by micromorphology (Figs 8–12) and macro scale stratigraphic analysis (Fig 3). The lowermost units of the Rietheuvel Member (“herbaceous sands”) were found to be archaeologically sterile by Deacon [10]. These herbaceous sands consist of very compact loam sand that can be differentiated into the following superimposed subunits, mostly following colour, occurrence of variously coloured mottles, and texture. The ‘Dark Mottled Herbaceous Sands’ (DMHS) are homogeneously blackish grey silty sands with minor darker variation, massive and compact, and crossed by long and thin subvertical brownish-yellowish mottles resembling stems, root traces and cracks. The colour grades northwards into lighter values (*sensu* Munsell©) and a defined contact could not be observed. The ‘Light Mottled Herbaceous Sands’ (LMHS) are light grey loamy sands with cm-size whitish grey rounded mottles. Also, in this case, the colour grades northwards into lighter values, and light brown to yellowish mottles (redoximorphic features) become frequent; the texture also becomes finer. Long/thin subvertical brownish-yellowish mottles are less common than in the DMHS. The contact is clear to gradual and dips gently northwards. The ‘Dark Brown to Black Herbaceous Sands’ (DBBCS) are in fact grey loamy sands with a slightly brownish hue and frequent whitish mottles, moderately coarser than the underlying ones. The colour lightens and redoximorphic features become extensive northwards; silt/clay also become more abundant. Long/thin subvertical brownish-yellowish mottles are relatively common. This unit is thicker to the north, tapering southwards until it disappears in the mid-southern part of the profile. The contact is clear to gradual and dips gently northwards, as for LMHS.

All these subunits dip northwards towards the margin of the basin. In fact, their clear to diffuse contacts and the common direction of colour/texture change suggest that they can be grouped in one lithologic unit whose characteristics grade radially within the basin, with darker values towards the centre. The sequence is punctuated by a series of what Deacon [10] called ‘Black Clay Bands’ (BCB) (Figs 3 and 4). The first of these, BCB1, is a homogeneous dark grey to blackish medium to fine sand layer, massive and compact, with few light mottles and long/thin redoximorphic features. As such, it is not actually dominated by clay but we have kept this terminology in the layer name so as to retain the correlation to Deacon’s [10] stratigraphy as much as possible. The contact of BCB1 with other units is sharp, horizontal and linear. The unit tapers abruptly southwards until it completely disappears. Sharp erosive contacts (Fig 4B) indicate that this layer represents a well-defined, independent lithologic unit, not a minor grain-size variation within a higher rank unit. Deacon [10] suggested a stratigraphic contact occurred at this level by means of a dashed line in his West Wall Section (Fig 3) but did not separate the upper Acheulian bearing deposits into a different member from these lower purportedly sterile units (DBBCS/LMHS/DMHS).

Above BCB1 are the Acheulian and wood bearing “Brown Herbaceous Sands” (BHS) facies (Fig 4A). Micromorphology shows that BHS consists of brownish-grey coarse to medium sands, slightly darker at the bottom, with common lighter mottles and some long/thin redoximorphic features (Figs 8 and 9). Several cm-size clods of dark grey sediment, very similar to BCB1, lie at the bottom of the unit (Fig 4B). The contact is sharp, subhorizontal and slightly undulating, and clearly erosive because it cuts the underlying BCB1 and contains rip-up clasts (Fig 4B). In the field, a clear erosional contact could be identified between the DBBCS and overlying BHS throughout the southern part of the deep sounding, as well as in the base of Cutting 1 Extension (Fig 4B). A thin “black clay band” (BCB2), 2-4cm thick, is interlayered within the BHS (Figs 3 and 4A). The limit is horizontal and sharp, suggestive of erosive processes prior to the deposition of this unit, which is an independent lithologic unit like BCB1, but occurring between two phases of BHS (Fig 4B). Two lens-like units occur within the overlying BHS unit in the southernmost part of the profile above BCB2. The lower one is light grey sand and the overlying one is light brownish coarse sand; both are diffusely mottled by redoximorphic features. Their lens-like profile is shaped by a concave-upwards erosion surface situated slightly northwards, which is poorly discernible because the whole profile is somewhat obscured here by strongly impregnated redoximorphic features. This erosion feature is filled up by fine and wavy laminae of alternating grey and white sand and silt, dipping parallel to the erosion surface.

Another black clay band, BCB3, which is in fact a layer of fine loamy sand, lies upon BHS (Figs 3 and 4A). It dips more steeply northwards than the other units. The contact is abrupt, and slightly undulating. This “band” should also be considered as an independent lithologic unit. BCB3 separates the wood and Acheulian bearing BHS from the Acheulian bearing ‘Greenish Clayey Sands’ (GCS). GCS only occurs in the very northern part of the Cutting 1 West Wall section because it was later heavily truncated by Deacon’s Disconformity (Fig 3). However, it can be traced along the rear wall of Cutting 10 where Deacon excavated Acheulian material from it. GCS is light greyish sandy clay loam, with medium developed blocky structure and extensive brownish redoximorphic mottling, mostly in the northern part. Its limits dips gently northwards and is sharp, apparently erosive also on BHS. GCS is overlain by a “Grey Black Silt” (GBS) bed that is a chaotic complex of strongly distorted and slumped layers of alternating dark and light grey layers and lenses (Fig 3). It lies unconformably upon GCS with a wavy erosional limit, which is partly masked by strongly impregnating redoximorphic features. Deacon [10] suggested the occurrence of typologically foreign intrusive archaeological elements into this unit.

After the deposition of GBS the sequence is interrupted by a major erosional feature (Deacon’s “Disconformity”) that cuts across several of the layers described above including BHS, the various clay bands, GCS and GBS (Fig 3). This marks Deacon’s [10] separation of his lower Rietheuvel and upper Balmoral Members. Deacon describes the lowest layer of the Balmoral Unit as the ‘Pothole Fill’ (PHF). The PHF is in fact a channel-like feature running roughly perpendicular to the western profile of Cutting 1 towards the west. The PHF is characterised by light greyish to brownish sandy loam, massive and compact, with diffuse redoximorphic patches. The contact with underlying units is sharp, concave and rather wavy. This unit contains a large Acheulian assemblage, but this has likely been eroded and deflated from the underlying Rietheuvel Member. The PHF is overlain by ‘Light Grey Sediments’ (LGS) which is a chaotic accumulation of unsorted (up to ~10cm) clods of different sediments, prevalently light or dark grey sands and silts, probably derived from the dismantling of GCS and/or GBS (Fig 3). The contact is sharp and slightly concave, but complex as if the clods were partly pressed into the underlying still uncompacted sediment. ‘Laminated Yellow Sediments’ (LYS) overlying this are medium to coarse yellowish sands, finely layered and locally cross-bedded. The contact is sharp and slightly concave-upwards. Above this are a series of iron-stained mottled grey facies that contain extensive ironstone blocks (Fig 3). All the Rietheuvel units, except the PHF, were said to be sterile of archaeology by Deacon [10].

### Radiocarbon

S1.5 Table in S1 File summaries the ABA AMS ^14^C ages for wood samples from the BHS in the centre of Area 1 (Square 2 extension). The ^14^C ages of wood samples AMZ1-259 (Wk 52004) and AMZ1-264 (Wk52005) are both indistinguishable from background and as such are considered older than ~53 ka based on the background standard measured in the same group of analyses.

### Palaeomagnetism

**Fig 5:**
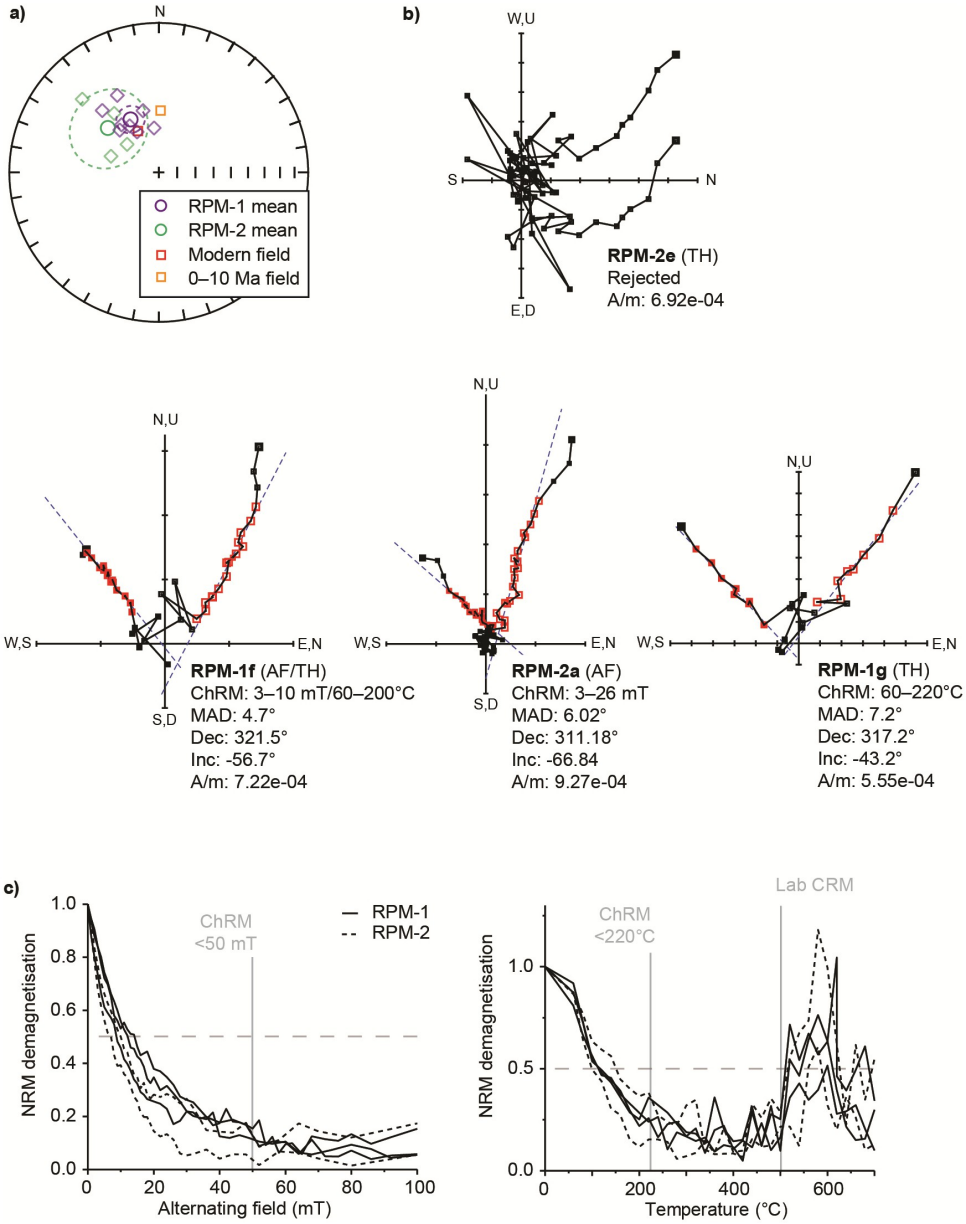
Palaeomagnetic data for Amanzi Springs Area 1. A) Stereographic projection showing Fisher (1953) mean directions for RPM-1 and RPM-2 and α95 confidence ellipses (dotted circles) with their respective subsamples (diamonds). Open symbols indicate a negative inclination. These are plotted against directions for the modern geomagnetic field at Amanzi Springs, and estimates for the time averaged dipole field here over 0–10 Ma. The latter was derived from an average of 0 Ma and 10 Ma reference data in [126] for the African Plate Apparent Polar Wander Path for Amanzi Springs in Paleomagnetism.org [127]. B) Representative vector AF, TH and AF/TH demagnetisation diagrams. Note that erratic data points have been removed in RPM-1g (>340°C) and RPM-1f (>280°C) for clarity, see rejected subsample RPM-2e for an example of this behaviour. C) AF (left) and TH (right) demagnetisation spectra (note that AF/TH spectra are not shown).

Final palaeomagnetic data (S1.4 Table in S1 File; Fig 5A) indicate that all samples exhibit normal magnetic polarity directions based off 12/15 accepted subsamples, consistent with an age younger than the Brunhes-Matuyama boundary (<~773 ka [125]). Natural remanent magnetisations (NRMs) ranged from 9.27–4.46 ×10^−4^ A/m, while χ_LF_ values were notably low at 0.057–0.023 ×10^−6^ m^3^/kg^-1^. Stable TH demagnetisation directions are noted up to ~220°C with the same directional components isolated via AFD up to 20–50 mT (Fig 5A). No major overprinting or multi-component NRM behaviour is observed via any of the demagnetisation methods with only minor directional biasing on initial demagnetisation steps (Fig 5B). The mineralogy of the samples is further explored in the S1 File and indicates a complex iron oxide-hydroxide and iron sulphide mineralogy. Despite the consistency of the palaeomagnetic data to other geochronological data indicating a <773 ka age, the complex iron sulphide mineralogy and uncertain remanence acquisition processes lead us to express caution in establishing that the normal polarity results are not more recent overprints.

### Luminescence dating

**Fig 6:**
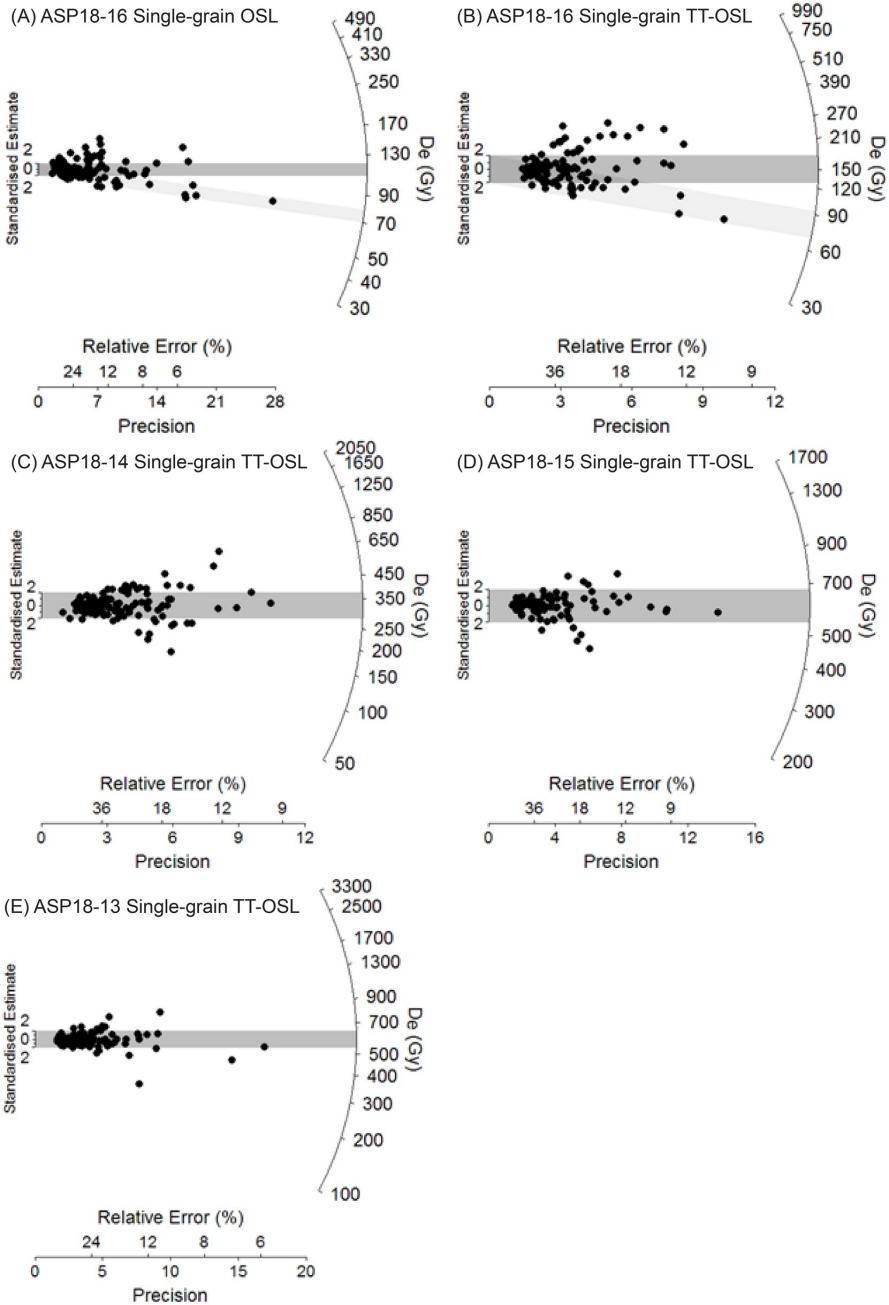
Single-grain OSL and TT-OSL D_e_ distributions for the Area 1 luminescence dating samples, shown as radial plots. The shaded bands are centred on the D_e_ values used for the age calculations, which were derived using either the 3-parameter minimum age model (light grey band sample ASP18-16) or the central age model (dark grey bands samples ASP18-14, ASP18-15, ASP18-13, ASP18-16) of Galbraith et al. [102].

**Fig 7:**
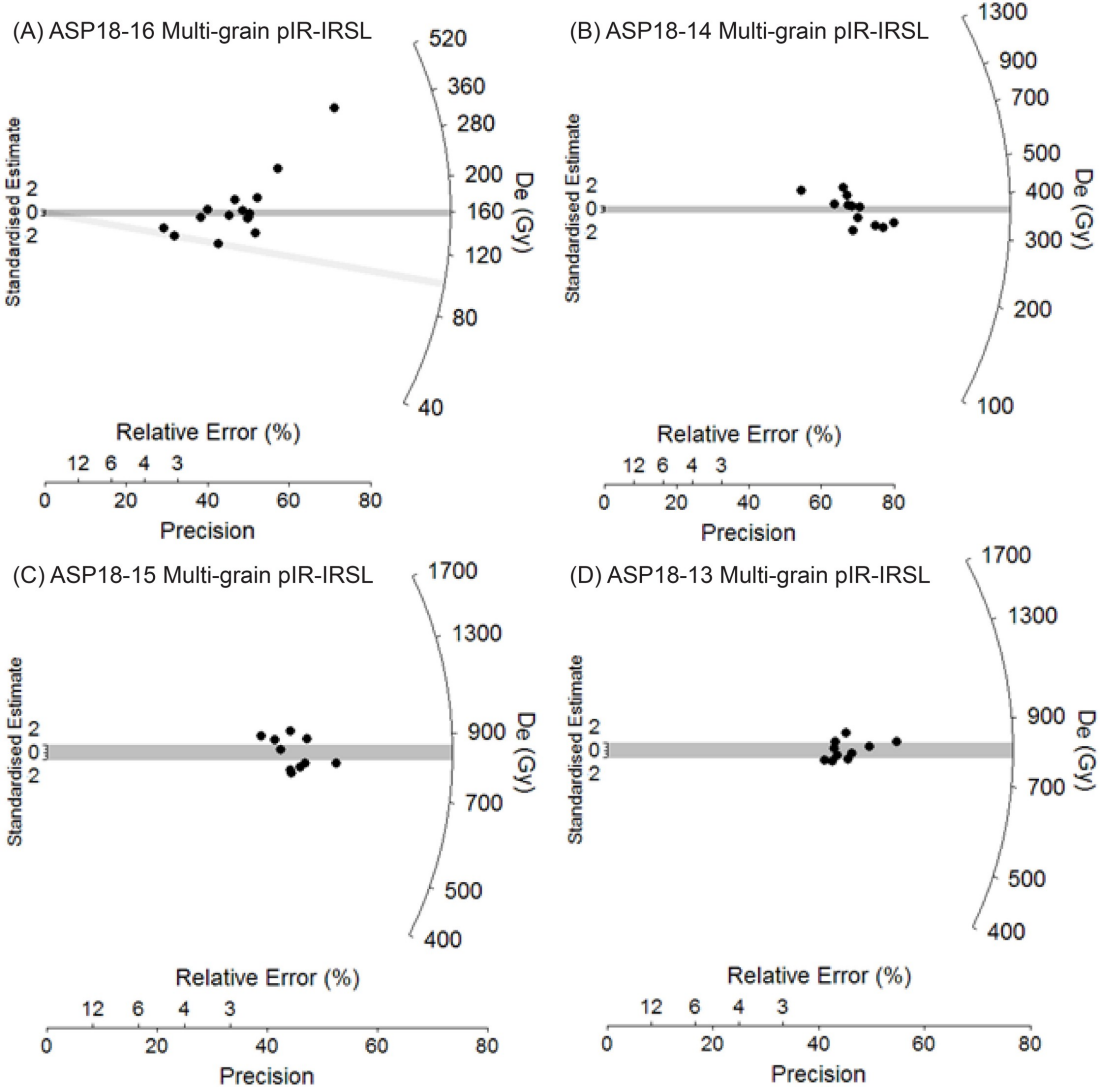
Multiple-grain pIR-IRSL D_e_ distributions for the Area 1 luminescence dating samples, shown as radial plots. The shaded bands are centred on the D_e_ values used for the age calculations, which were derived using either the 3-parameter minimum age model (light grey band sample ASP18-16) or the central age model (dark grey bands samples ASP18-14, ASP18-15, ASP18-13, ASP18-16) of Galbraith et al. [102].

Table 1 summarises the environmental dose rates, D_e_ values and final ages obtained for the four luminescence dating samples and additional data is provided in S1 File and S1.1-S1.3 Tables in S1 File. The single-grain TT-OSL, single-grain OSL and multiple-grain pIR-IRSL D_e_ distributions of each sample are also shown as radial plots in Figs 6 and 7 and abanico plots in S1.5 and S1.6 Figs in S1 File. The pIR-IRSL D_e_ datasets of samples ASP18-13, ASP18-14 and ASP18-15 have low overdispersion values ranging between 5 and 14% and are not considered to be significantly skewed according to the multi-grain version of the weighted skewness test outlined by Arnold et al. [128] and Arnold and Roberts [129]. The single-grain TT-OSL D_e_ distributions of these three samples are characterised by moderate dose dispersion, D_e_ scatter that is generally well-represented by the weighted mean value (as indicated by the large proportions of grains lying within the 2σ grey bands on Fig 6), and low to moderate overdispersion of 25–39%.

The overdispersion values for these samples are consistent with, or slightly higher than, those typically reported for ideal (well-bleached and unmixed) single-grain TT-OSL D_e_ datasets at 2σ (e.g., the average overdispersion of 21 ± 2% presented by Arnold et al., [106]). These single-grain TT-OSL D_e_ datasets also do not display prominent leading-edges of low D_e_ values or elongated tails of higher D_e_ values, and are not considered to be significantly positively skewed according to the weighted skewness test outlined by Bailey and Arnold [130] and Arnold and Roberts [129]. Taken together, these single-grain TT-OSL and pIR-IRSL D_e_ characteristics suggest that insufficient bleaching prior to burial [e.g., 128, 131–135], post-depositional sediment mixing [e.g., 76, 129, 135] or beta dose rate heterogeneity [e.g., 136–138] have not contributed excessively to the D_e_ scatter of samples ASP18-13, ASP18-14 and ASP18-15. The final burial doses for these three samples have therefore been derived using weighted mean D_e_ values, calculated using the central age model (CAM) of Galbraith et al. [102] (Table 1).

Sample ASP18-16, which was collected from LYS at the base of the Balmoral Member (Fig 4), exhibits heterogeneous TT-OSL, OSL and pIR-IRSL D_e_ distributions (Table 1, Figs 6 and 7) characterised by high dose dispersion, large proportions of individual D_e_ values lying outside of the weighted mean burial dose 2σ ranges, and noticeable leading-edges of low D_e_ values or tails of higher D_e_ values. The single-grain OSL and pIR-IRSL D_e_ datasets are additionally considered to be significantly positively skewed according to the single- and multi-grain weighted skewness tests outlined by Bailey and Arnold [130] and Arnold and Roberts [103, 129]. Overdispersion values of 51%, 63% and 35% were obtained for the single-grain OSL, single-grain TT-OSL and multiple-grain pIR-IRSL D_e_ datasets of sample ASP18-16, respectively (Table 1). These overdispersion values are systematically higher than those typically reported for ideal (well-bleached and unmixed) single-grain and multiple-grain D_e_ datasets at 2σ [e.g., 131, 134], and they are significantly higher than the values obtained for the single-grain OSL, single-grain TT-OSL and multiple-grain pIR-IRSL dose recovery tests undertaken on samples ASP18-13 and ASP18-16 (i.e., overdispersion values of 13 ± 2%, 26 ± 4% and 1 ± 2%, respectively). Collectively, these D_e_ distribution characteristics suggest that dose dispersion originating from extrinsic, field-related sources, and / or intrinsic experimental sources not captured by the dose recovery test, have exerted a significant influence on sample ASP18-16.

Post-depositional mixing or bioturbation are not thought to have contributed significantly to the heterogeneous D_e_ dataset of ASP18-16 given the preservation of clear stratigraphic layering in the LYS horizons. However, given the depositional context of Area 1 –which was intermittently dominated by localised alluvial, slopewash and fluvial processes, as well as low-energy ponds / swamps during spring reactivation events–it is feasible that the additional D_e_ scatter observed for ASP18-16 may be attributable to heterogeneous bleaching at the time of deposition. Additional insights into the bleaching adequacy of this sample can be gained by comparing the weighted mean D_e_ values of the three luminescence dating signals. Given that OSL, pIR-IRSL and TT-OSL signals bleach at different rates in natural daylight (e.g., [64, 131, 139–141])), parity in quartz and K-feldspar luminescence ages would suggest that the sample was exposed to sufficient daylight to reset its residual doses prior to burial. If this was not the case, the three luminescence dating signals would yield markedly different weighted mean ages on account of their distinctly different bleaching sensitivities. The CAM age for the single-grain OSL and pIR-IRSL datasets of ASP18-16 are consistent with each other at 1σ (86.0 ± 7.3 ka and 86.8 ± 9.2 ka, respectively; Table 1). The TT-OSL CAM age of ASP18-16 is ~25ka older than its OSL and pIR-IRSL counterparts, though all three ages are consistent at 2σ (Table 1). The broad similarity of these weighted mean ages provides support for adequate resetting of the luminescence signals (at least the OSL and pIR-IRSL signals), all things being equal. This would imply that the enhanced scatter observed across the three D_e_ datasets may primarily arise from intrinsic sources of D_e_ scatter [e.g., 142] and/or spatial variations in beta dose rates experienced by individual grains. The latter is conceivable for the LYS Layer given the quartz-rich nature of this deposit and the potential for radioactive ‘hotspots’ related to the presence of isolated minerals with high internal radioactivities (e.g. rutiles, zircons, feldspars, clay minerals; see Micromorphology section below). While differences in beta dose rates of individual grains could explain the enhanced D_e_ scatter of the single-grain OSL and TT-OSL datasets, it is unlikely to explain the D_e_ scatter of the pIR-IRSL dataset; multi-grain averaging effects and the higher internal beta dose rate contributions of the K-feldspar grains themselves would significantly diminish the relative effects of grain-to-grain variations in external beta dose rates for the pIR-IRSL D_e_ dataset.

Further work is required to fully resolve the origin of the D_e_ scatter affecting this sample, including the collection of modern analogues from active spring deposits to directly assess bleaching characteristics of individual signals, and undertaking single-grain K-feldspar measurements to examine possible multi-grain averaging effects. Pending these follow-on assessments, we have opted to use both the CAM and the minimum age model (MAM) to derive OSL, TT-OSL and pIR-IRSL burial dose estimates for ASP18-16 (Table 1), since these two age models collectively cover the full range of feasible D_e_ interpretative scenarios. It is worth emphasising that ASP18-16 is the least important sample for the archaeological interpretations of Area 1 as it is derived from the archaeologically sterile LYS layer, which overlies all *in situ* Acheulean-bearing layers at the site. It was primarily dated to compare to radiocarbon dating done in the 1960s. From this perspective, the decision of whether to use the CAM or the MAM has minimal impacts on our final archaeological findings, but still establishes the deposits as being beyond the age limit for radiocarbon.

Though we were unable to collect a suitable modern analogue sample from Area 1 in this study, a weighted mean pIR-IRSL residual dose of 22.6 ± 2.3 Gy was recorded for sample ASP18-13 following 8 h of daylight bleaching (as part of the dose recovery test; see S1 File). This residual D_e_ equates to 2.8% of the natural D_e_ value for sample ASP18-13 and yields a corresponding residual age of 11.4 ± 1.3 ka, which lies within the existing 1σ uncertainty of the final pIR-IRSL age estimate for this sample (Table 1). Given (i) the relatively small size of the empirical residual D_e_ values recorded in the dose recovery test, (ii) the insensitivity of the final age of ASP18-13 to this residual dose estimate, and (iii) the unknown bleaching durations experienced by each of the dating samples prior to deposition (i.e., natural bleaching durations may have significantly exceeded the 8 h experimental bleaching durations employed in the dose recovery experiments), we have not considered an additional residual dose subtraction in the final pIR-IRSL age estimates.

The long-term (athermal) stability assessments undertaken on the Area 1 K-feldspar fractions yielded weighted-mean *g*-values (normalised to 2 days) ranging between 1.1 ± 0.2%/decade and 1.5 ± 0.3%/decade per sample (S1.3 Table in S1 File), with a combined weighted average g-value of 1.4 ± 0.1%/decade for all measured aliquots (*n* = 12). These empirical fading rates are consistent with published *g*-values for higher temperature pIR-IRSL signals (e.g., pIR-IRSL_290_ signals involving pIR-IR stimulation at 290 °C following a preheat of 320 °C for 60 s; [see summary in 62]). Such low g-values (on the order of <1–2%/decade) have been interpreted to be potentially unreliable indicators of long-term fading rates and / or artefacts of laboratory procedures on the basis of comparisons made with independent age control, observations of natural signal saturation, and measurements of similarly sized g-values for quartz [e.g., 143–145]. Consequently, we do not consider the low g-values recorded in the present study to be indicative of the need for additional pIR-IRSL_250_ age corrections; and though fading corrected pIR-IRSL ages are also presented in S1 File (S1.3 Table in S1 File) for comparative purposes.

The replicate pIR-IRSL, TT-OSL and OSL ages shown in Table 1 are consistent with each other at either 1σ or 2σ for all four samples, which supports the validity of the luminescence chronologies obtained in this study. The consistency of the uncorrected pIR-IRSL age and the replicate single-grain OSL age for sample ASP18-16 also supports our contention that an empirical fading correction does not appear to be necessary for the pIR-IR_250_ signals of these samples.

### Micromorphology

#### Basic mineral components

**Fig 8:**
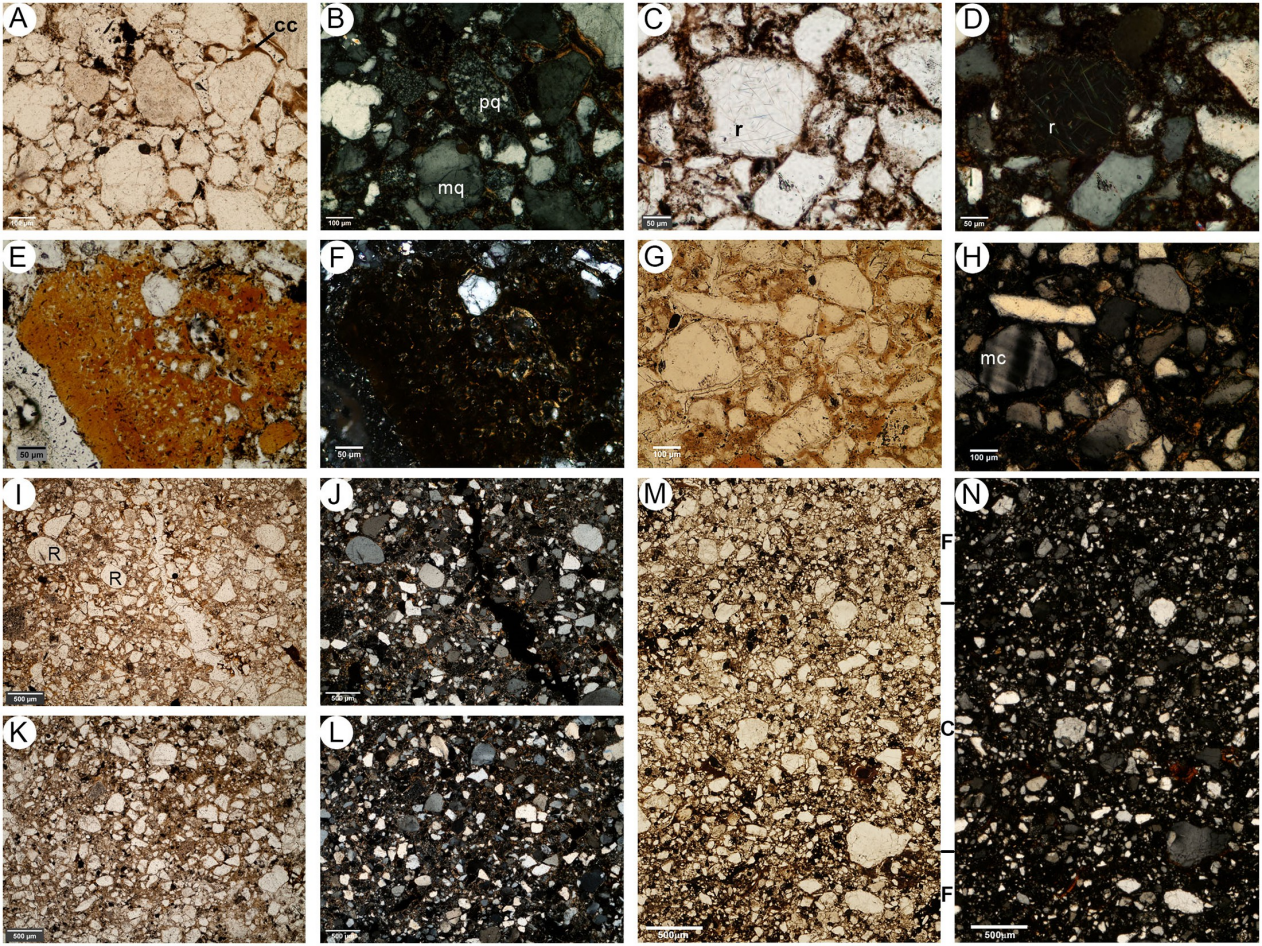
Microphotographs of mineral components and textures of the Amanzi Springs Area 1 sediments sequence. A) subangular to subrounded sand grains, brown illuvial clay coatings (cc) and black amorphous organic matter; parallel polarized light (PPL); DMHS (sample AMZ17/5/1). B) same as in A) under crossed polarized light (XPL), showing monocrystalline (mc) and polycrystalline (pc, probably chert or quartzite) quartz grains; note the medium continuous orientation b-fabric of the clay coatings. C) quartz grain with needle-like rutile inclusions, PPL; DMHS (sample AMZ17/5/1). D) same as in C), XPL. E) very coarse sand-size glassy granule, with perlitic alteration structures, PPL; DMHS (sample AMZ17/5/1). F) same as in E), XPL; note high-birefringence devitrification rims around perlite features. G) relatively abundant reddish-brown clay organised in enaulic and chitonic related distribution pattern among sand grains, PPL; BHS (sample AMZ17/4/3-4-5, top horizon). H) same as in G), XPL; quartz and microcline (mc) sand grains and low-birefringence stipple-speckled b-fabric of clay. I) relatively common rounded (R) sand grains, PPL; LMHS (sample AMZ17/2/3-4, lower part). J) same as in I), XPL. K) sand texture dominated by angular particles; fine fraction more abundant than in I); BHS (sample AMZ17/2/3-4, upper part). L) same as in K), XPL. M) crude layering of fine (F) and coarse (C) horizons, the fine ones including more amorphous organic matter, PPL; DMHS (sample AMZ17/5/1). N) as in M, XPL.

Mineral composition is remarkably homogeneous throughout the Amanzi Area 1 sequence, with moderate variation related uniquely to the relative abundance of clay in different lithologic units. Quartz is largely dominant within the mineral assemblage, mostly as monocrystalline grains; polycrystalline quartz grains–micro- or even cryptocrystalline, sometimes including variable amounts of white mica are also present (Fig 8A and 8B). Rutile needle-like crystals are sometimes included in quartz (Fig 8C and 8D). Feldspar grains, as plagioclase, some altered K-feldspar and perthite, are less common and generally smaller than the quartz grains (Fig 8H). A few larger (up to 1.5 mm), rounded particles composed of reddish altered glass and scoria, sometimes with perlitic structures, also occur within all the samples (Fig 8E and 8F). Greenish minerals, probably pyroxene, occur occasionally together with some grains with upper-order interference colours, probably high-stability minerals like zircon and titanium oxides, which are too small to be identified reliably.

Sand is the most common grain-size class within the coarse fraction (c/f [coarse/fine] limit at about 10 μm) for all samples (Fig 8I–8N), even in Deacon’s [10] ‘Black Clay Bands’. It is usually poorly sorted, with maximum size around 400–500 μm, with the smallest fractions merging into the silt spectrum. Angular and subangular particles are dominant, with some rounded or sub-rounded elements. The grain-size tends to be uniform within the massive and homogeneous units, even if with moderate local variation in irregularly distributed areas, whereas distinct grain-size classes characterise the laminae of finely layered units (Fig 8M and 8N). For almost all samples, the <10 μxm fine fraction includes dusty fine silt and clay; the latter is usually not very abundant and is often concentrated in pedofeatures like infillings or coatings (Fig 8A), or in a chitonic/enaulic related distribution pattern with stipple-speckled b-fabric (Fig 8G and 8H). The fine fraction and b-fabric are often masked by widespread amorphous organic matter in dark organic-rich layers. Clay is comparatively abundant in BHS and in the northern facies that represent deposits on the spring eye margin, and in the ‘Black Clay Bands’ (BCB1, sample AMZ17/2/3-4). Here, the silt grain-size class is scarce and a large part of the spaces among sand grains are filled with clay, whose dominant related distribution pattern is chitonic with granostriated b-fabric. Enaulic and porphyric patterns are also frequent, displaying stipple- or mosaic-speckled b-fabric (Fig 8G and 8H). The aggregation state of the sediments is always massive, and the microstructure is massive as well, with frequent packing voids. Voids originating from roots are relatively common.

#### Organic matter and sediment colour

**Fig 9:**
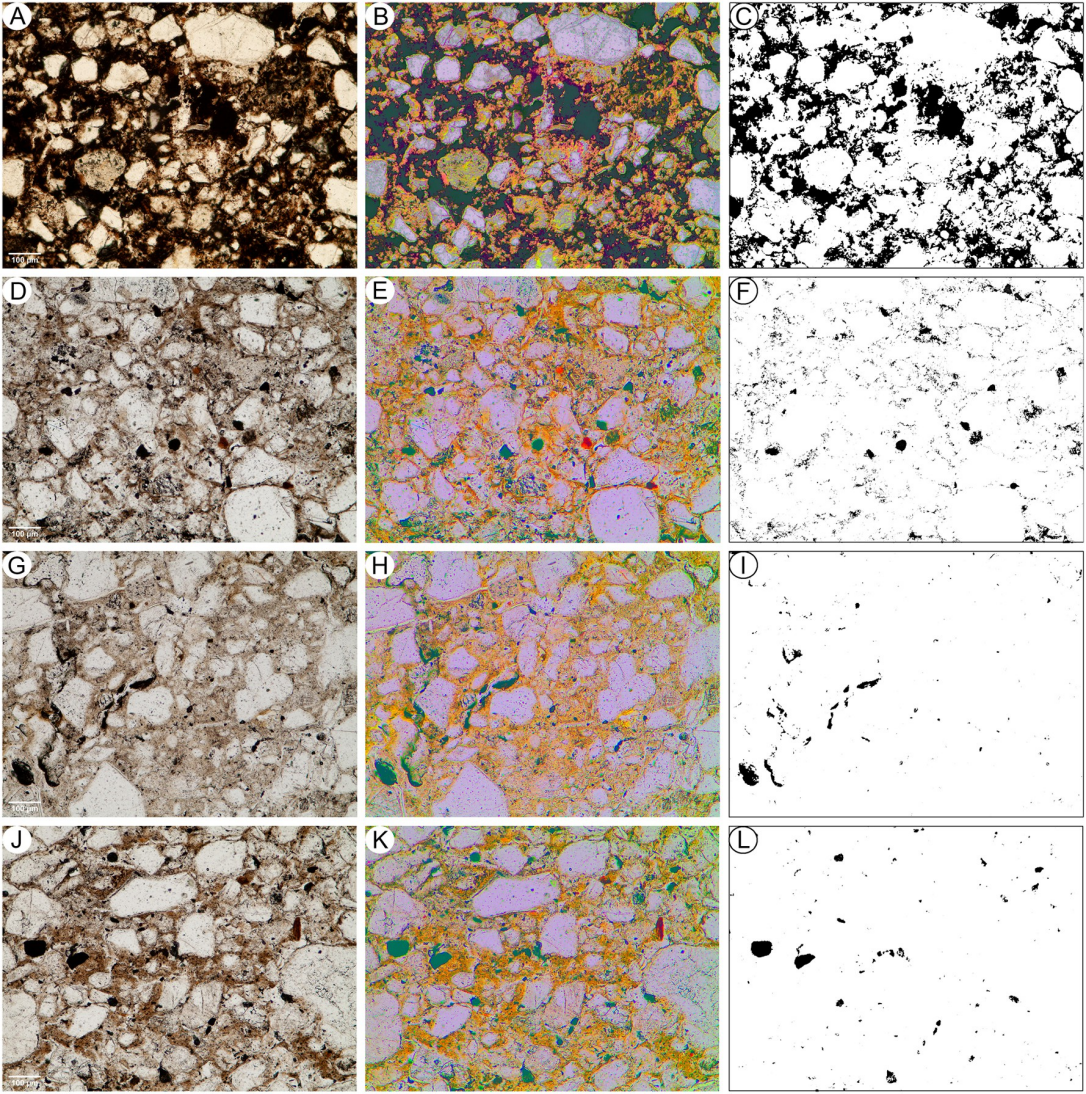
Distribution and size of amorphous organic matter aggregates (AOM) in the Amanzi Springs Area 1 sediments. Left column: thin sections microphotographs (PPL). Central column: results of microphotograph decorrelation stretching (stretching = 0.01); green: AOM; yellow to orange-red: increasingly impregnated Fe-oxide pedofeatures and micromass; blue: Mn oxides; some colour change near the margins is due to uneven illumination (not visible in normal conditions). Right column: AOM aggregates after extraction by ImageJ colour thresholding. A-C: DMHS (sample AMZ17/5/1), with dominant large AOM aggregates. D-F: LMHS (sample AMZ17/1/3-5, lower part), with isolated medium/small-size aggregates and very fine AOM punctuation around mineral grains. G-I: northern facies of LMHS (LMHS-N, sample AMZ17/2/3-4), with few relatively large AOM aggregates and abundant fine silt/clay. J-L: BHS (sample AMZ17/1/3-5 upper part), with unsorted few to common AOM aggregates in locally Fe-impregnated clay-rich micromass.

**Fig 10:**
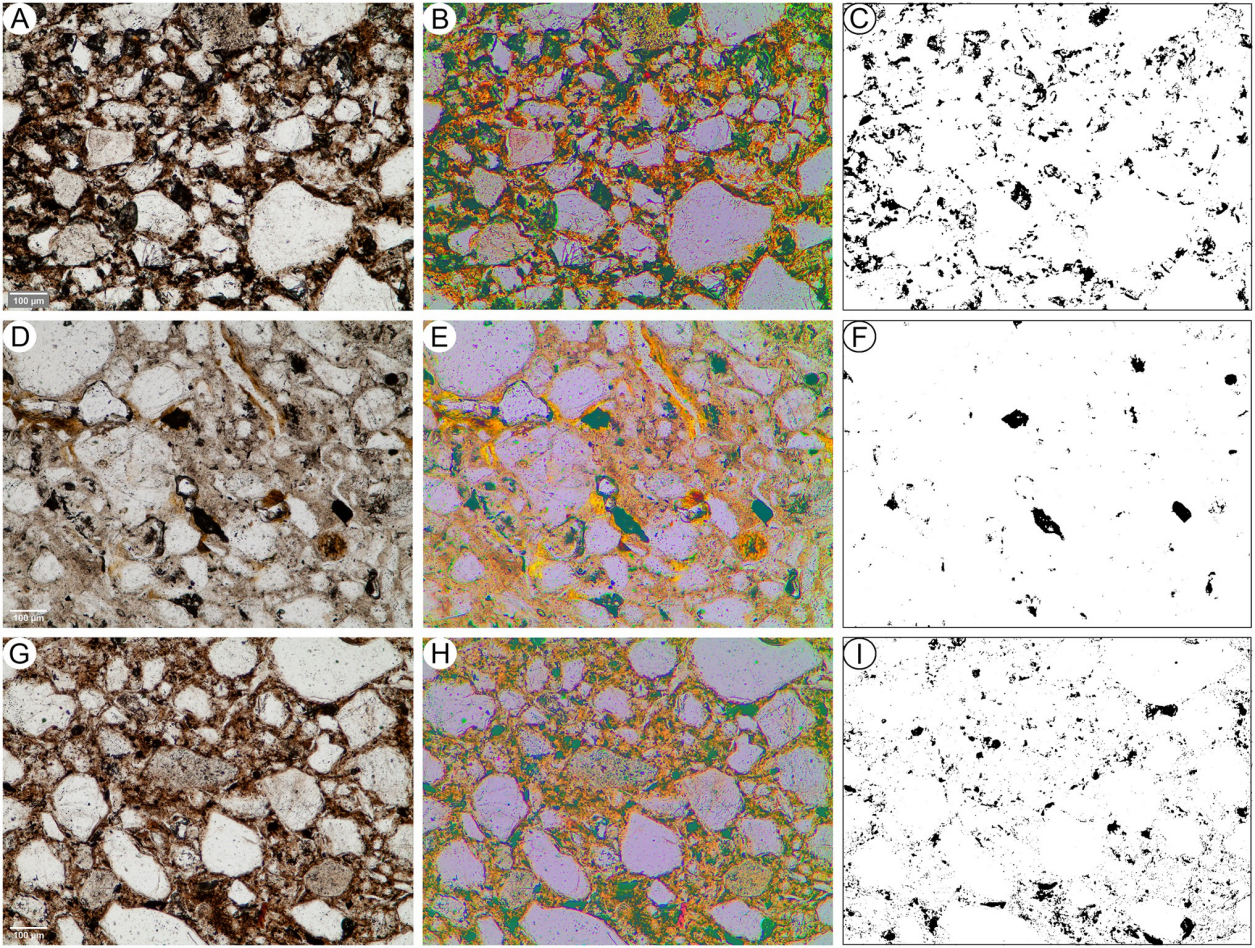
Micromorphology of the black clay banks. A-C: “black clay band” BCB1, (sample AMZ17/4/3-4-5), with dominant unsorted AOM aggregates and frequent Fe-oxide impregnations of the micromass. D-F: northern aspect of BCB1 (BCB1-N, sample AMZ17/2/3-4, upper part), with isolated large AOM aggregates and sparse punctuation and strong Fe-oxide impregnation of the micromass. G-I: clod of BCB1 in BHS (sample AMZ17/1/3-5, upper part), with frequent to dominant unsorted AOM aggregates.

**Fig 11:**
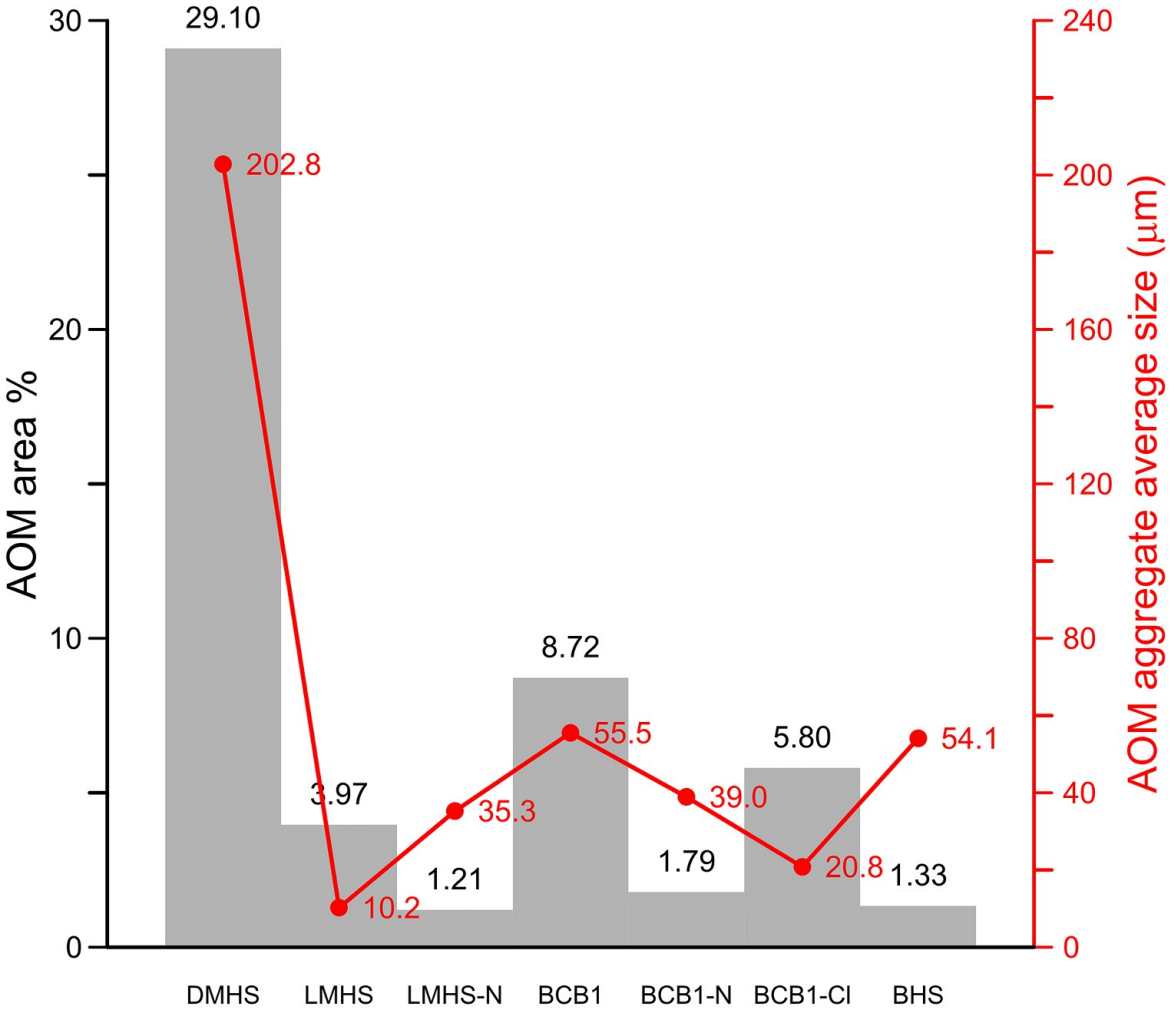
Amorphous organic matter content. Amorphous organic matter (AOM) content in DMHS, LMHS, BCB1, BHS, in the northern facies of LMHS (LMHS-N) and BCB1 (BCB1-N), as well as in a rip-up clast of BCB1 (BCB1-Cl) included in BHS. Quantity expressed as percentage of the whole microphotograph area (grey histogram) and aggregate size (red line), after extraction by decorrelation stretching using MathWorks MatLab R2019a, colour thresholding and particle measurement using ImageJ [109].

The dark grey or blackish colour of the various facies of herbaceous sands (DMHS, LMHS, DBBCS) and of the intercalated black clay bands (BCB1 and BCB3, and very likely the unsampled BCB2) is due to organic matter, which is widespread throughout the sediments. Organic matter is amorphous black and completely opaque in thin section (Figs 9A–9L and 10). Only in some cases, it exhibits a brownish-reddish hue due to impregnation by iron oxides. Amorphous Organic Matter (AOM) occurs in irregular (Fig 9A–9C and 9G–9I) to subrounded aggregates (Fig 9D–9F and 9J–9L), organised in an enaulic related distribution pattern among mineral grains; Fig 9 shows their quantity and size within distinct units. These aggregates, ubiquitous in the dark units, are often grouped in tiny lens-like features (Fig 8M and 8N), not more than 2-3mm thick and up to 1-2cm long, which mark otherwise undetectable depositional surfaces. The sand grains included within these lenses are often finer than in the adjacent amorphous organic matter-poor laminae. Aggregates of amorphous organic matter decrease dramatically in quantity and become particularly fine (Fig 11) in the lighter, more mottled northern subfacies associated within the same units (Fig 9D–9F).

The various units grouped under herbaceous sands differ mostly because of variation in organic matter content, whereas the mineral assemblage is largely constant, suggesting that they represent internal grading within the same lithologic unit. DMHS are intensely black and particularly rich in amorphous organic matter (Fig 9A–9C), which is much more abundant here than in any other layer of the sequence. The size of the aggregates (Fig 11 is also by far larger here than in any other unit. No (or extremely rare) traces or residues of vegetal cells, tissues or organs can be detected throughout the observed thin sections. DMHS and LMHS differ only because AOM aggregates are fewer and smaller in LMHS, whereas diffuse redoximorphic features give the northern part of DBBCS its brown hue. BHS differs from the lower herbaceous sands (DMHS, LMHS, DBBCS) because of its lighter value due to fewer but relatively large AOM aggregates, which are also somewhat compact and subrounded, whereas its brownish hue derives from a higher amount of clay stained by Fe oxides.

Amorphous organic matter is less abundant in BCB1 than in DMHS, and the aggregates are also smaller (Fig 10A–10C) despite its colour being only moderately lighter. Unlike in DMHS, large and identifiable fragments of vegetal tissue occur rather frequently in the dark (southern) part of this unit. These are mostly parts of leaves and stems, usually lying along depositional surfaces and strongly compressed by diagenetic pressure. All these units become lighter to the northern side of the profile, where AOM is fewer though organised in larger aggregates (Figs 9G–9I, 10D–10F and 11).

#### Black blay bands and erosional features

**Fig 12:**
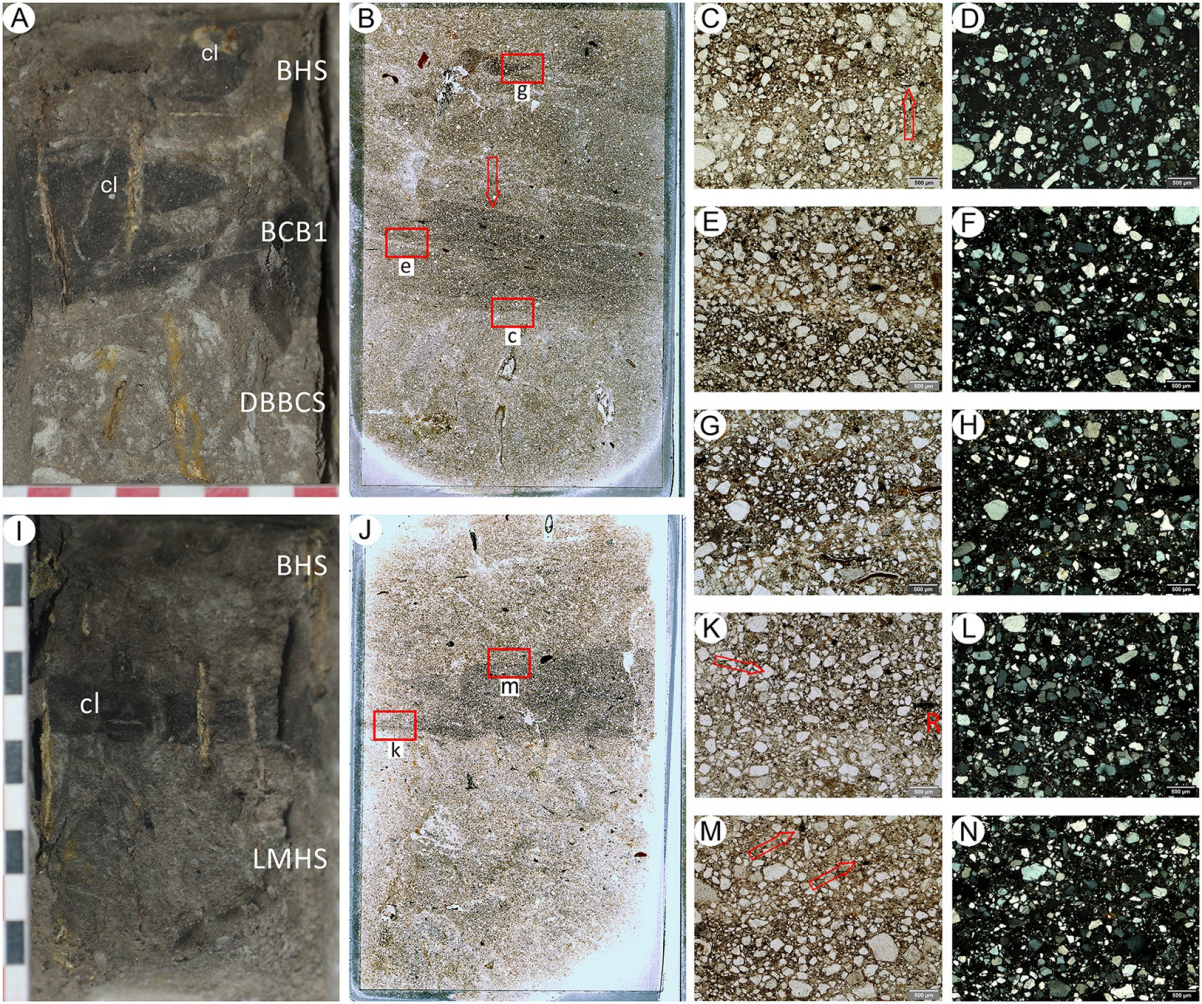
Contacts between lithologic units at the boundary between the archaeological layers of the upper part and sterile layers of the lower part of Deacon’s [11] Rietheuvel Member. A: Sequence of Light Mottled Herbaceous Sand (DBBCS) overlain by Black Clay Band (BCB1) and Brown Herbaceous Sands (BHS), with erosive contact between BCB1 and BHS. Clods (cl) of BCB1 are embedded within BHS, sometimes lying on BCB1 top (detachment niche of sample AMZ17/4/3-4-5). B) High-resolution scan of thin section from the same sample, showing relatively sharp, probably non erosive boundary between LMHS and BCB (c), dark AOM-rich and light AOM-poor fine laminae (e), clod of BCB in BHS (g); note that the thin section scan is turned rightside left because of thin section preparation. C) microphotograph as in (c): dark BCB1 layer overlying moderately sharp boundary with DBBCS (PPL). D) as in B), XPL; quartz grains in DBBCS are slightly more rounded than in BCB1. E) as in (e): finely laminated BCB1 with coarse AOM aggregates and AOM-poor lamina at the centre (PPL). F) as in E), PPL. G) as in (g): dark, AOM-rich clod embedded in BHS, showing strict resemblance with BCB1 (PPL). H) as in G), XPL. I) Sequence of LMHS overlain by BHS, with erosive contact between LMHS and BHS, much sharper than in B and C. Clods (cl) of BCB1 are reworked into BHS, lying on LMHS top (sample AMZ17/1/3-5). J) High-resolution scan of thin section from sample AMZ17/4/3-5, showing very sharp erosive boundary between LMHS and a clod of BCB embedded in BHS (k). The top boundary (m) of the clod is diffuse, probably because of BCB1 fine material diffusely embedded in BHS; note that the thin section scan is turned rightside left because of thin section preparation. K) as in (k), sharp boundary between LMHS and BCB1 clod (red arrow); a 0.5 to 1mm-thick layer (R) of reworked BCB1 material, or in situ residue of BCB1, lies on LMHS (PPL). L) as in K, XPL. M) diffuse limit between BCB1 clod and embedding BHS, probably with finely dispersed BCB1 sediment reworked into BHS (arrows) (PPL). N) as in M, XPL.

Unit BCB1 separates the lower archaeologically sterile parts of the Rietheuvel Member from the Acheulian layers in the top half of the Member, at ~3m depth [11; Fig 3]. Field observations suggest its bottom contact may be erosional because it is an abrupt horizontal surface. Conversely, the upper contact is much sharper and clearly erosional, abruptly cutting BCB1 with a concave surface that completely removes it in the southern part of the profile. In the lower part of BHS (BHSL), clods of blackish sediment resembling BCB1 occur as rip up clasts close to the contact with the underlying BCB1 and -to the south- with DBBCS (Figs 4 and 12), also suggesting erosion. Micromorphological monoliths were collected in two loci representative of this context. Sample AMZ17/4/3-4-5, encompasses the top of DBBCS, BCB1 and the bottom of BHS; sample AMZ17/4/3-5 was collected slightly southwards where erosional contact between LMHS and BHSL occurs with no intervening BCB1, which has been entirely removed by erosion (Figs 3 and 4), and where a rip up clast lies on the boundary.

The microscopic-scale aspect of the contact between the in situ BCB1 and the underlying DBBCS (sample AMZ17/4/3-4-5) is relatively sharp (Fig 12B–12D), but not as clear-cut as the evident erosional contact between BHS and LMHS (Fig 12J–12L), suggesting that BCB1 may have accumulated directly upon DBBCS, without intervening erosive processes. The upper contact of BCB1, in contact with BHSL, is not as sharp as between BHSL and DBBCS, which is in contrast with its apparent erosive nature. However, fine laminations in BCB1 (Fig 12B, 12E and 12F), suggest that erosion and short-range transport in low energy flow regimes reworked and mixed together the top of BCB1 and BHSL, and consequently created a less distinct boundary. The micromorphological characteristics of the blackish clods embedded at the bottom of BHS (Fig 4B) closely resemble those of BCB1, showing they originated from this unit. AOM is less abundant in the clods than in the southern part of BCB1, though its related distribution pattern is the same (Fig 10A–10C and 10G–10I), suggesting that the clods were eroded from the northern part of BCB1, where AOM is less abundant, as in all other units.

A sample (AMZ17/3/9) encompassing the ‘Disconformity’ reported by Deacon [10 Fig 3] at the bottom of PHF marks the contact between the Rietheuvel and Balmoral Members. The base of the sequence observed at microscopic scale corresponds to upper layers of BHS (BHSU) and consists of a grey layer whose characteristics do not differ substantially from what is observed in BHSL, with the difference that dusty clay infilling within pores indicate translocation of fine/colloidal components by percolating water. The irregular wavy laminae in the overlying laminated sandy silt range from whitish to brownish to dark greyish and follow a scheme of cyclical fining-up sequences ending with clay layers. These laminae testify to phases of energetic transport followed by pooling in a depression, prior to the formation of the ‘Disconformity’. Deacon’s [11] third Black Clay Band’ (BCB3) consists of dark grey sandy silt loam and overlies this unit with a sharp boundary moderately dipping northwards. Some lenses situated at the top of BCB3 include several very well-preserved remains of vegetal organs, mostly leaves. BCB3 is overlain by whitish silty sand with reddish mottles, which partly corresponds to the eroded southern end of GCS, and partly to PHF sediment derived from reworking of GCS.

A striking characteristic of the Amanzi Springs Area 1 sequence is the total absence of carbonates within the sediments, which consequently also do not include mollusc shells or other mineral remains of carbonate-producing animal or vegetal taxa. Besides the absence of any animal life, the sequence is also devoid of remains relating to non-carbonate producing taxa, e.g. diatoms. The absence of carbonates also contrasts with the composition of the local bedrock, which is calcareous mudstone, and with common depositional processes (geochemical or biogenic calcite, tufa, etc.) that would be expected in marshy environments. Butzer [56] found extremely low pH values (around 3.0–3.5) for the sediments he collected. Carbonate removal from bedrock sediments, as well as null preservation of phytoplankton biogenic carbonates, may be due to the hydrochemistry of the thermal springs (25–37°C [122]) that were active until a few years ago in the site area. Though pH data are apparently not available in literature, hydrochemistry data indicate soft waters (Mg^2+^ 10 mg/l, Ca^2+^ 8 mg/l, SO_4_^2-^ 15mg/l, alkaline ions 14 mg/l, total ions 105 mg/l [146] and Fe^2+^ 48.5 mg/l [147, 148]) with low pH, probably acidified by the sulfate ions. Modern iron concretions in the irrigation channels and ferricretes cropping out around the site also point to acidic water reaction, which may have been one of the causes for carbonates removal, shrinkage of vegetal and animal populations, and accumulation of vegetal detritus in shore areas [149].

Pedofeatures other than the redoximorphic ones are not very common in the Amanzi Area 1 sequence. Coatings of brownish Fe-stained clay with moderate continuous orientation occur sparsely within all samples. These coatings may be located within vertical channels dug by roots or within (sub)horizontal lens-like cracks. Fe-oxides occur commonly in all samples as impregnative redoximorphic pedofeatures, usually staining the clay of the coatings. Thick redoximorphic coatings and hypocoatings of amorphous Fe-oxides (reddish-yellowish at eye-scale) occur in the bottom DBBHS along elongated vertical voids. Passage pedofeatures represented by local disruption of the layering or by chaotic rearrangement of the particles are relatively common and apparently represent the only traces of animal life, but are somewhat difficult to identify and delimit because of subsequent compaction of the sediments. Redoximorphic features are common throughout the sequence, though they are not very strongly developed in the lower units despite the marshy nature of the site. The reason for their scarce occurrence may relate to the very low amounts of Fe-bearing minerals in the Amanzi Springs sediments, which are mostly composed of quartz and feldspars. Impregnation by Fe-oxides in the upper part of the sequence is conversely rather strong and may be due to subsequent development and weathering of the ferricretes that cover part of the surrounding area.

### Archaeological analysis

#### Assemblage composition and integrity

**Fig 13:**
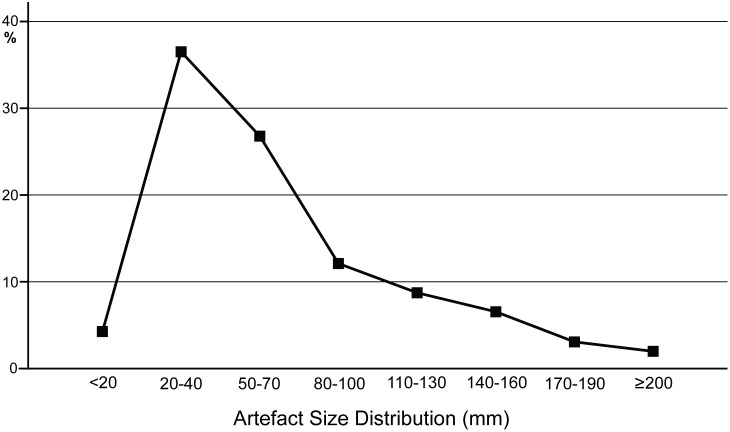
Artefact size curve. Artefact size curve indicating a lack of materials <20mm.

The Area 1 lithic assemblage that was analysed comes exclusively from the BHS and GCS units and its metric data is presented in Table 2 and S2.1-S2.10 Tables in S1 Data. The lithic assemblage demonstrates both small debitage (production of flakes that were retouched and/or used as blanks) and LCT production chains, which typifies the Acheulian industry (Table 2) [116, 117, 150]. While cores and small to medium-sized flakes (20–111 mm) demonstrate small debitage production occurred on site, roughouts and *éclats de taille de biface* flakes document the production of LCTs at Amanzi Springs. A massive scraper made on an *éclat entamé* and utilized flakes struck from giant cores also corroborate such activities. In terms of assemblage integrity, the artefact size curve is skewed towards the 20 to 40 mm range, while materials under 20 mm (3.9%) are under-represented within the assemblage (Fig 13). Thirty-five percent of the assemblage is in a fresh condition and 26% are slightly weathered. Heavily weathered artefacts (14%) are lowest in proportion, although 26% appear ‘rolled’ with high polish and well-rounded edges.

**Table 2:** Typology and Raw material of stone artefacts.

Typology	Quartzite	Hornfels	Igneous	Quartz	Sandstone	Silcrete	N	%
**Debitage**							**263**	**24%**
*Complete*	88		1			1	90	8.2%
*Incomplete*	30						30	2.7%
*Split*	15						15	1.4%
*Éclats de Taille de Biface*	35						35	3.2%
*Core Maintenance*	8						8	0.7%
*Bipolar*	2						2	0.2%
*Preferential*	4						4	0.4%
*Fragment*	79						79	7.2%
**Cores**							**244**	**22%**
*Unifacial*	87			1			88	7.9%
*Bifacial*	71	1			2		74	6.7%
*Discoidal*	42						42	3.8%
*Multifacial*	11					1	12	1.1%
*Prepared*	15						15	1.4%
*Core-on-Flake*	2						2	0.2%
*Fragment*	11						11	1.0%
**LCTs**							**145**	**13%**
*Handaxes*	78				4		82	7.4%
*Cleavers*	28						28	2.5%
*Picks*	2						2	0.2%
*Roughouts*	21						21	1.9%
*Fragment*	12						12	1.1%
**Heavy Duty Tools**							**28**	**2.5%**
*Core Tools*	3						3	0.3%
*Utilized Flakes*	21						21	1.9%
*Massive Scrapers*	3						3	0.3%
*Knives*	1						1	0.2%
**Retouched**							**4**	**0.4%**
*Scrapers*	3						3	0.3%
*Denticulate*	1						1	0.1%
**Debris**							**419**	**38%**
*<2cm*	43						43	3.9%
*>2cm*	374					2	376	34.1%
**Total**	**1090**	**1**	**1**	**1**	**6**	**4**	**1103**	**100%**
**%**	**98.8%**	**0.1%**	**0.1%**	**0.1%**	**0.5%**	**0.4%**	**100%**	

#### Raw materials

Raw material use at Area 1 was highly conservative with 98.6% of tools made on pre-Cretaceous quartzites clasts derived from the Peninsula Formation Sandstone Formation (aka Table Mountain Sandstone), while silcrete makes up less than 1% of the assemblage (Table 2). Lithological properties observed on 545 of the tools reveal an abundance of poorly-sorted, course textures (67.3%), while 32.9% of artefacts were classified as well-sorted, fine-grained in texture, and 14% of artefacts showed medium-grained textures. The cortical surfaces on cores and LCTs point towards selection for raw material package morphologies. A predominance of ellipsoidal, subangular package shapes were noted amongst the cores chosen for reduction, while LCTs reflected the selection of elongated, tabular package shapes. A survey of Amanzi Hill and the local Coega river bedload revealed that these package shapes were abundant and thus the availability of raw materials appropriate for small debitage and LCT operational chains may have been a driving factor for quartzite selection.

#### Assemblage components

**Fig 14:**
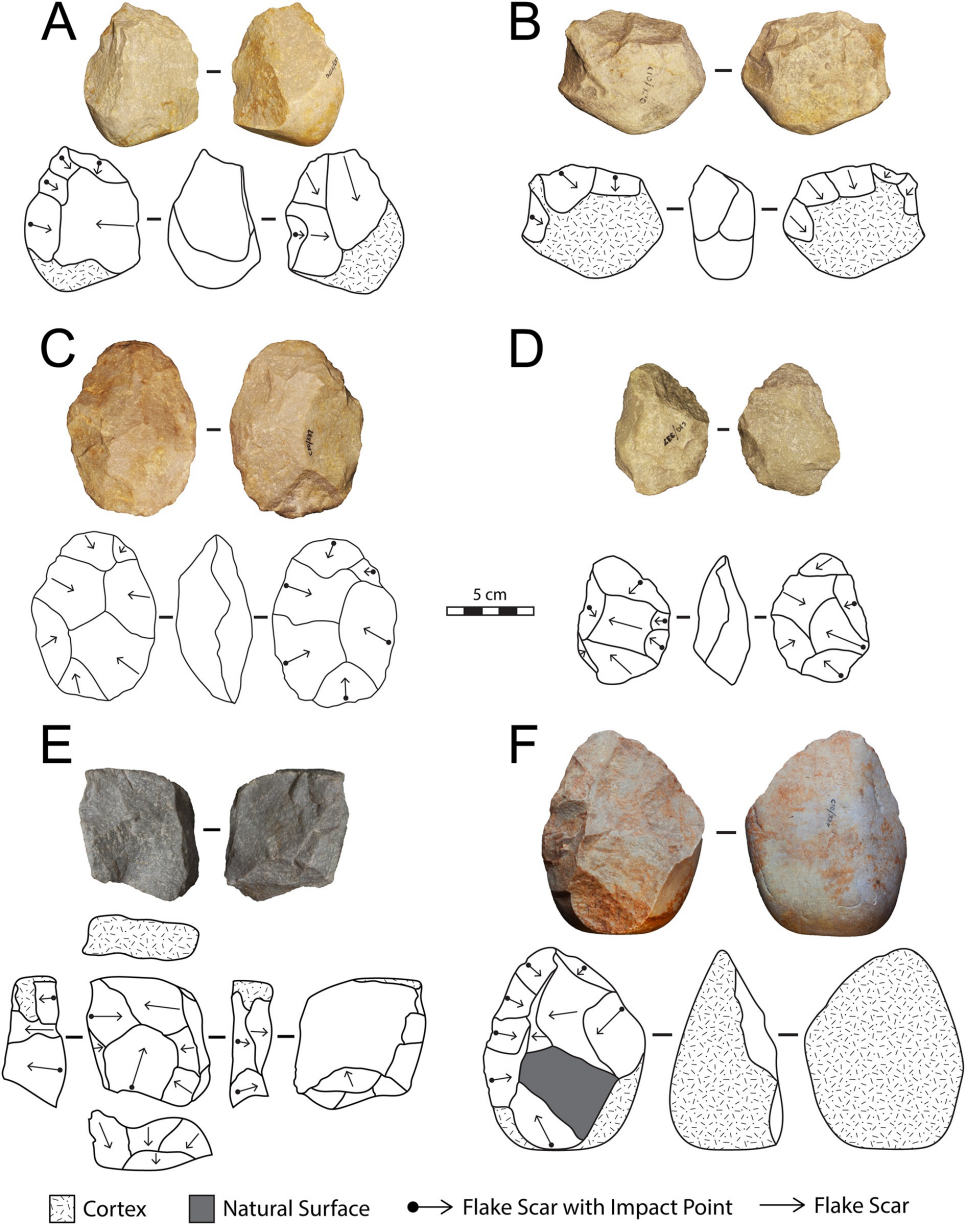
Examples of small debitage core from Area 1. A) Bifacial Alternate; B) Bifacial Continuous; C) Discoidal Continuous; D) Discoidal Alternate; E) Prepared; F) Unifacial.

*Cores*. Within the core assemblage (n = 244), five major reduction systems including unifacial (35.2%), bifacial (30.7%), discoidal (17.2%), multifacial (4.9%) and prepared (6.1%) categories were identified, as well as two cores-on-flakes (Fig 14; Table 2). S2.1 and S2.6 Tables in S1 Data present metric variables recorded for the Area 1 cores. The unifacial system (n = 88) predominates core reduction strategies, showing parallel (n = 41; 47%) and isolated (n = 39; 44%) and centripetal (n = 8; 9%) flaking patterns, mostly carried out through perpendicular angles of percussion. Cortex percentages of unifacial cores are highest, peaking between 51–90% and average flake scar count is only 3.3.

The bifacial system (n = 74) was comprised of alternate (n = 36; 49%), continuous (n = 28; 38%), alternating (n = 8; 11%) and hierarchical (n = 2; 3%) rotation schemes. Bifacial alternate cores are characterized by series of isolated and parallel flake scars carried out through secant angels of percussion. Bifacial alternating schemes predominantly showed isolated flake scars, which used scar negatives as platforms for subsequent removals after the core was rotated 180°, and secant angles of percussion. Bifacial continuous cores showed a mix of parallel, orthogonal and sub-radial removal patters on core faces before rotation and mostly secant angles of percussion. Bifacial cores average 11–30% cortical surfaces and 7.08 flake scars. The discoidal systems (n = 42) were predominantly comprised of alternate (n = 23; 55%), followed by alternating (n = 14; 33%) and continuous (n = 5; 12%) rotation schemes, all via secant angles of percussion. Cortical surfaces were mostly removed completely and an average of 12.7 flake scars were found on discoidal cores. Prepared cores (n = 15) showed signs of managing convexities on flaking surfaces, clear patterns of hierarchical treatment of core surfaces and preferential removals. Prepared cores averaged 11–30% cortex and 9 flake scars and are relatively large in size (averaging 94.45 mm in length and 373.65 in mass) (S2.1 and S2.6 Tables in S1 Data). The multifacial system (n = 12) is predominated by isolated flake removal patterns (n = 8; 67%) that aimed to exploit the longest surfaces possible, with no signs of organization reduction that define polyhedral cores. Cortex percentages for multifacial cores are evenly distributed between 0–50% and an average of five flake scars was recorded.

**Fig 15:**
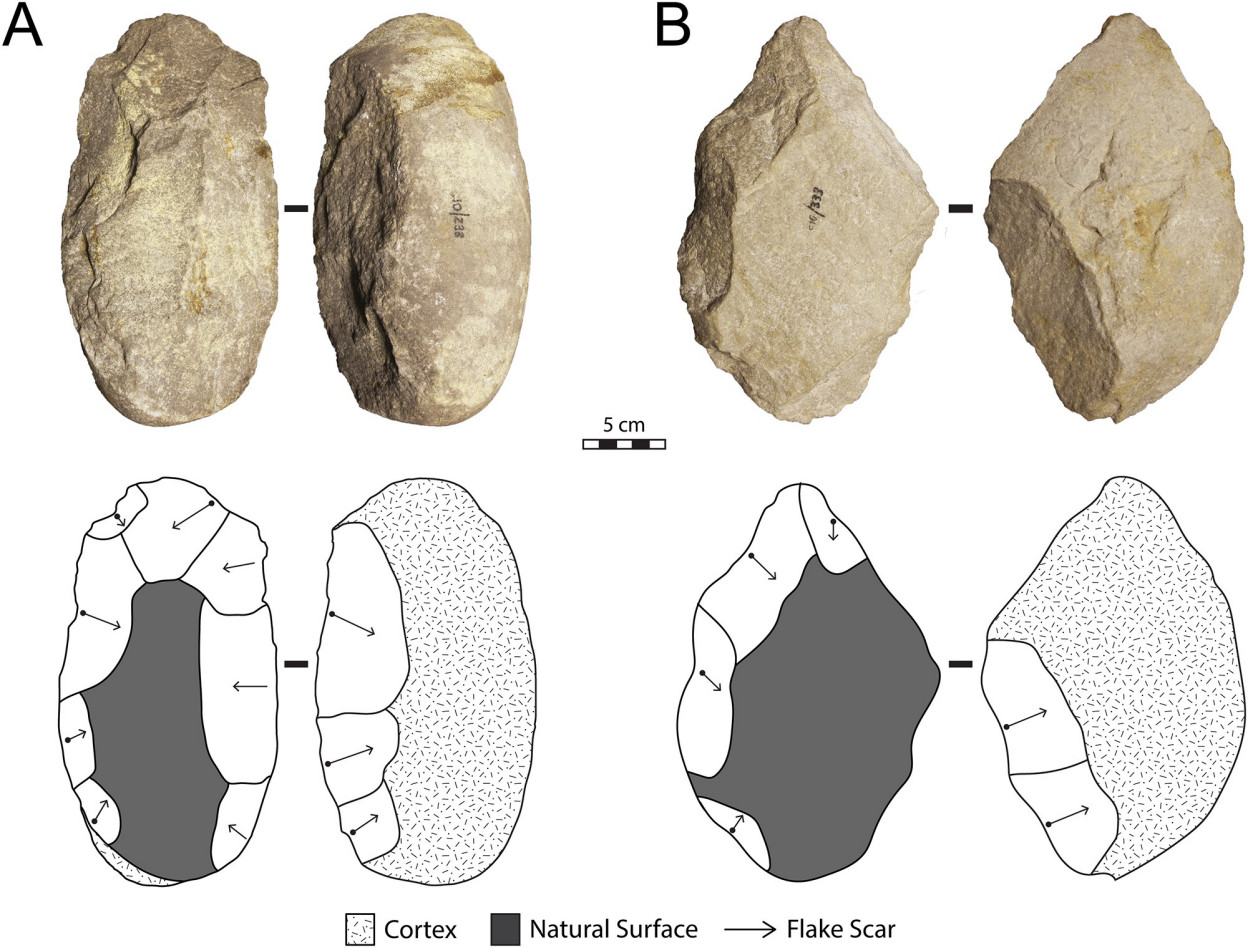
Two giant cores form Area 1 demonstrating the early phases of large cutting tool production. A) Naturally halved cobble exhibiting bifacial reduction related to LCT production; B) Spall exhibiting potential tip shaping.

It should be noted that three giant cores were identified, which range one to two standard deviations above the small debitage cores in all metric dimensions (Fig 15). While small debitage cores average a combined mass of 444.4g, the three giant cores weigh 2232g, 2214.5g and 3000g, respectively. They bare flaking patterns that attempt to shape one end into a tip or rounded end, which seemingly reflects the early phases of LCT shaping procedures.

**Fig 16:**
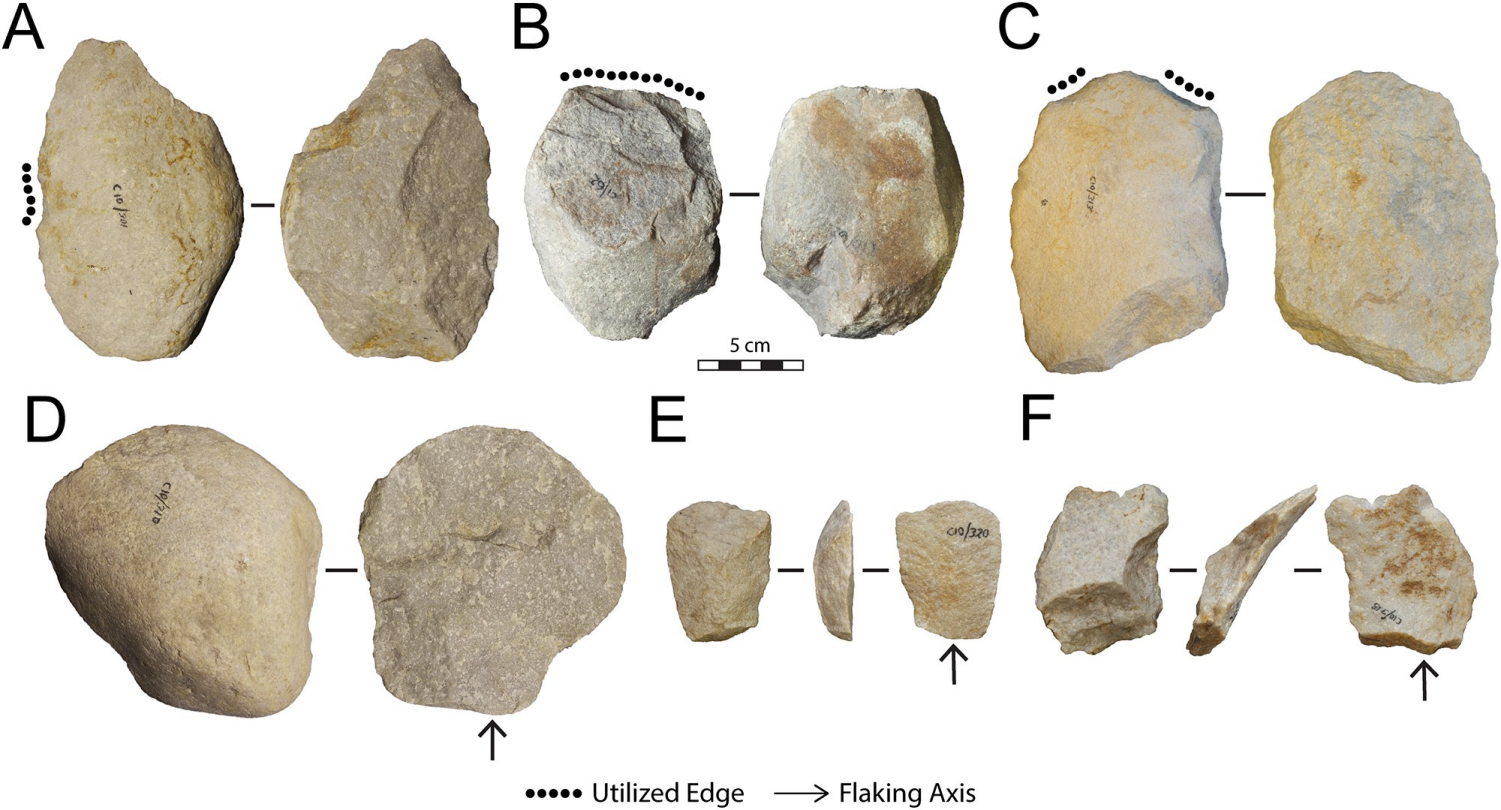
Utilized tool and flakes from Area 1. A) Massive scraper made from an éclat entame struck from a giant core; B) Core tool; C) Utilized Flake (éclat entame struck from a giant core); D) Éclat entame struck from a giant core; E) Complete flake; F) Thinning flake.

**Fig 17:**
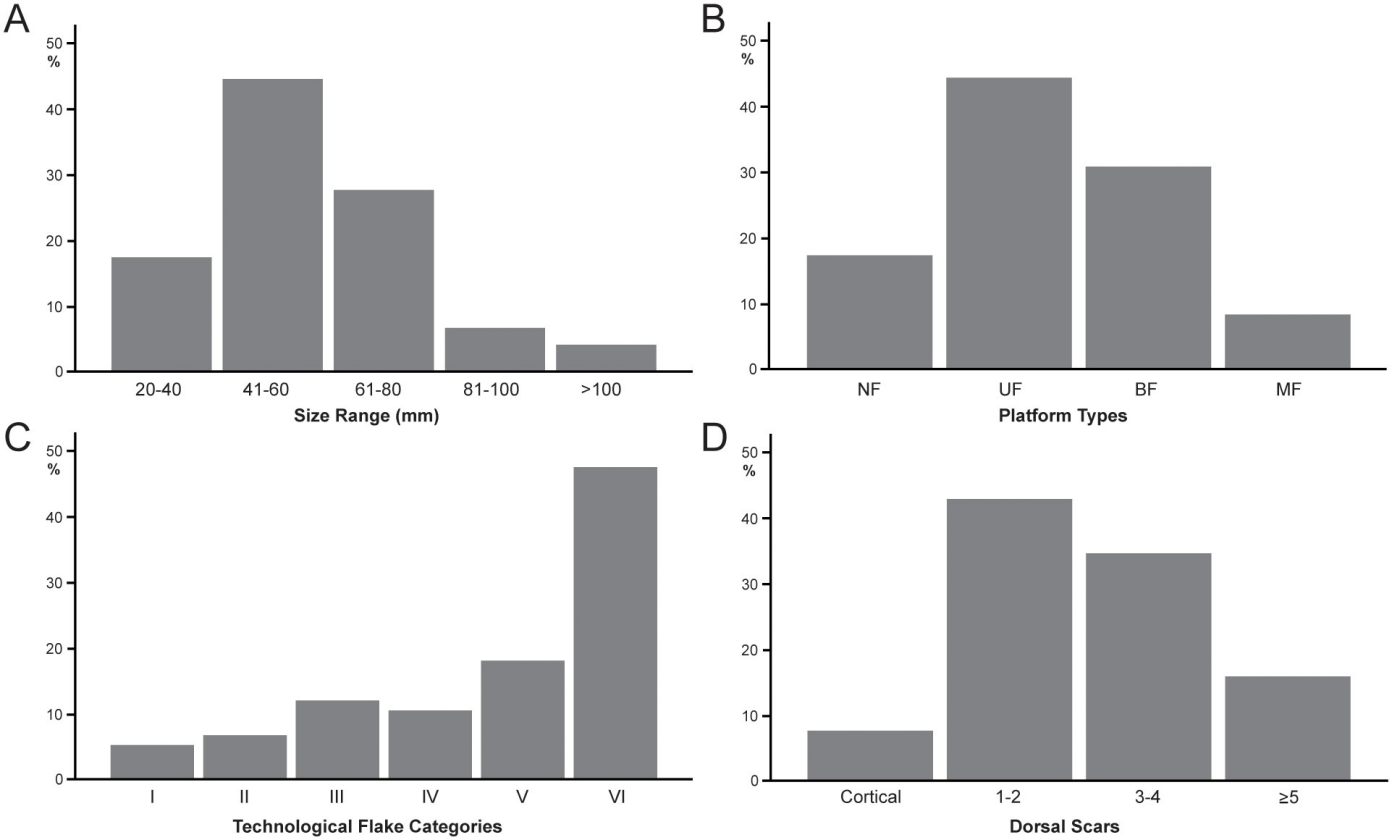
Bar graphs of flake attributes. A) Flake size ranges; B) Platform types; C) Technological flake categories; D) Number of dorsal scars.

*Débitage*. Débitage comprises 24% of the Area 1 assemblage and complete flakes average 65 mm in maximum dimension (S2.2 and S2.5 Tables in S1 Data). In terms of reduction elements, 17 shaping and 18 thinning flakes relating to LCT production, and five core edge maintenance, two core rejuvenation flakes and one core tablet were identified (Fig 16). Fig 17 displays debitage attributes showing that flakes range between 41–80 mm in length. Plain and dihedral platforms are most prevalent suggesting low amounts of preparation and rotation underlying core reduction. Technological Flake Categories are skewed towards types V and VI, which lack cortical surfaces on platforms and most of their dorsal surfaces (Toth, 1985). Lastly, dorsal scars on flakes were relatively low, also corroborating short reduction sequences underlying small debitage production. The ratio of total number of debitage pieces (n = 236) to total number of flake scars on cores (n = 1427) equates to 1:17, which suggests that there are components of the flake assemblage under-represented at Area 1. When considering the lack of cortical flakes, these patterns may point towards fragmentation in the small debitage production chain [151]. Cortex removal phases in core reduction potentially happened elsewhere, or cortical flakes may have been transported, which requires further investigation. Lastly, one large *éclat entame* struck from a giant core was identified, signifying the early phases of LCT reduction (Fig 16).

*Retouched and heavy-duty tools*. Possible signs of use-wear (n = 23) were identified in a small component of the retouched tools (n = 4) and flakes (S2.8 Table in S1 Data). Three scrapers and one denticulate were identified as exhibiting abrupt retouch inclinations. Utilized tools were largely composed of small, unretouched flakes (<10cm) with localized usewear patterns along their edges. Three large flake blanks struck from giant cores also exhibited signs of usewear and three massive scrapers were identified based a combination of retouch along their edges (Fig 16). Usewear patterns were uniform across tools types, comprising large notches with stacked stepped fractures. While these usewear patterns require further study before they can be definitively linked to specific activities, they are indicative of heavy-duty activities, such as chopping and/or scraping [123].

**Fig 18:**
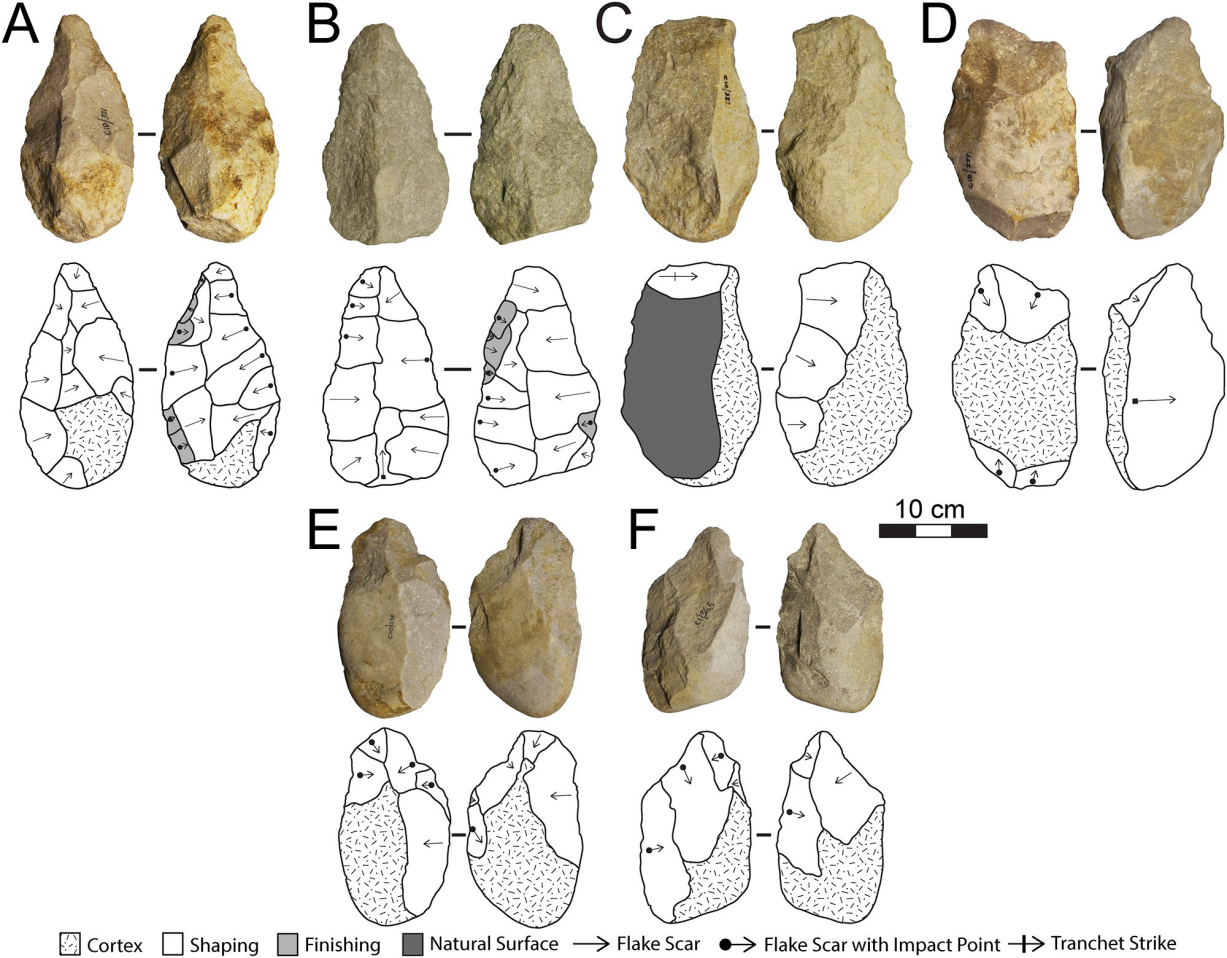
Large cutting tool from Area 1. A&B) Handaxes; C&D) Cleavers; E&F) Roughouts.

*Large cutting tools*. The LCTs from Area 1 comprises 13% of the lithic assemblage, with relatively high proportions of handaxes (n = 82) and cleavers (n = 28) (S2.3 and S2.7 Tables in S1 Data). Picks (n = 2) are infrequent, while other elements including roughouts (n = 21) and knives (n = 2) are present (Fig 18). Cleavers from Area 1 are relatively large and produced through a variety of shaping methods. S2.3 Table in S1 Data shows that the metric dimensions of these tools from Area 1 exhibit the highest amounts of variation when compared to handaxes and roughouts. In terms of production, cleavers were shaped bifacially from large cobbles and also through a variety of large-flaking techniques (S2.4 Table in S1 Data). Side and end struck flakes were identified, suggesting that cleavers were shaped on large cores and subsequently detached. *Éclat entame* and slab-slice core reduction methods were associated with cleaver production [37, 152]. Overall, cleavers are relatively ‘unstandardized’ and vary considerably in terms of their morphology when compared to handaxes within the Area 1 assemblage. Cortex percentages on cleavers ranged between 11–40%, mostly located towards their bases and averaged 14.8 flake scars.

Handaxes show the lowest ranges of variation in terms of metric measurements compared to other LCT forms, which likely reflects more extensive reduction sequences (S2.3 Table in S1 Data). In terms of production, bifacial reduction was the most prominent shaping technique for handaxes, although side- and corner-struck flakes document the use of large flake blanks. (S2.4 Table in S1 Data) [37, 152]. Despite the later Acheulian designation of this assemblage, the handaxes lack signs of extensive thinning and retouch along their edges, and their maximal width and thickness measurements tend to have larger ranges than other Acheulian assemblages [see 14, 153]. Cortex percentages on handaxes ranged between 0–30%, concentrated toward their midlines and bases, with an average of 15.5 flake scars. Furthermore, we reclassify Deacon’s [11] ‘other biface’ and ‘unstandardized’ categories as LCT roughouts (n = 21), which are characterised by their large dimensions (>100 mm) and bifacial shaping, yet are relatively ambiguous as either handaxes or cleavers. While ‘other biface’ categories are recognized across eastern and South African Acheulian assemblages [154, 155], their bifacial shaping patterns suggest they were connected to LCT production chains rather than end products. We interpret these artefacts as LCT preforms that represent the early stages of manufacturing processes. Three roughouts have definitive signs of pointed tip-shaping first identified by Deacon [11] at the site, which undoubtedly situates some of these artefacts within a handaxe reduction continuum. In terms of size, roughouts are on average larger than handaxes in all dimensions and cleavers in width and mass (S2.3 Table in S1 Data). Cortex percentages ranged from 11–50%, concentrated on the midsection and base ends, and an average of 12 flake scars were noted on roughouts.

## Discussion

### The stratigraphy and chronology of Area 1

Deacon [10] and Butzer [56] termed the sequences occurring across the Area 1 and 2 springs as the Amanzi Springs Formation. This was based on the idea that similar looking deposits in both springs were contemporary. The Council of Geoscience Map 3325DA (ADDO) has continued to use the term ‘Amanzi Formation’ for an area around the springs (Qa) that is described as ‘Sand, Silt and Clay’. The maps also list this Formation as Holocene in age. This is problematic for several reasons. The area showing Qa on the maps is not in the right location, the site is not Holocene in age, and currently there is no evidence that similar looking deposits in Area 1 and 2 are contemporary. For this reason, and due to the added complexity of the sequence as described above, we propose that the Member system described by Deacon [10] and Butzer [56] for Amanzi Springs is no longer appropriate. Instead, we refer to individual facies based on the sedimentological descriptions of Deacon [10] where appropriate, or new descriptions when describing newly recognised facies.

The Area 1 sequence represents a series of alternating environmental conditions related to increasing and decreasing water flow from the spring eye, punctuated by periods of erosion, some of which relate to expansion of the spring pool, while others relate to significant phases of erosion and deflation when the spring was not active (i.e Deacon’s ‘Disconformity’). Analysis of the exposed stratigraphy in Deacon’s [10] West Wall of Cutting 1 and its deep sounding identify issues with the way Deacon [10] and Butzer [56] reconstructed the stratigraphy of Area 1. Deacon [10] only identifies the ‘Disconformity’ as the main erosional event in the upper part of this sequence, separating the Rietheuvel and Balmoral Members. However, it is clear from broad scale stratigraphic studies, micromorphology, and luminescence dating that the whole sequence consists of punctuated periods of deposition covering a period of at least ~300–350 ka.

The luminescence dating study indicates that the Acheulian-bearing BHS and GCS both formed within MIS 11. BHS formed at ~404 ka (weighted mean of TT-OSL and pIR-IR; 403.9 ± 23.4 ka), whereas the stratigraphically younger GCS is perhaps only ~14 ka younger at ~390 ka (weighted mean of TT-OSL and pIR-IR; 389.6 ± 24.5 ka), however both ages overlap within error and suggest they could either be formed extremely close in time to each other or up to 60 ka apart, but we consider the latter case to be less likely. Work by Roberts et al. [156] at Klein Brak River, Dana Bay and Hartenbos River near Mossel Bay some ~360 km to the west of Amanzi Springs, indicates that the sea level was 13–14 m higher at 391 ± 16 ka, within error of the age for the BHS/GCS sequence using similar methods. Roberts et al. [156] interpret this as a period when sea level was falling after the earlier sea level high stand. With a +13-14m sea level rise along this section of the southern Cape coast, a Ria would have formed along the Swartkops Valley (Swartkops Palaeoria) and would have come to within ~7 km of Amanzi Springs. It is possible that a similar ria could have formed along the Coega River, making the site even closer to the coast, but the estuary it is too anthropogenically altered today to model this. Based on MIS 11 ages for BHS it can be inferred that the episode responsible for the truncation of the underlying sterile archaeological units BCB1 and DBBCS (lower Rietheuvel as per Deacon [10]) may have been related to the onset or peak of MIS 11 at ~420 ka. Future dating of these units will hopefully resolve this uncertainty. During our 2019 season we finished the re-excavation of Deacon’s deep sounding and cut back the sections for sampling. In doing so we established that these lower units, while not as rich as the BHS/GCS layers, do contain archaeology, both within the very basal units that Deacon classified as the Rietheuvel Member and at the contact with the underlying ‘White Sands’ of the Enqhura Member. This indicates that Area 1 may yield even older Acheulian artefact assemblages in the future.

A significant time gap exists between the deposition of the BHS/GCS Acheulian layers GBS, which appears to have accumulated at the transition from MIS 7 to 6 at ~190 ka (weighted mean of TT-OSL and pIR-IR; 189.7 ± 2.8 ka). Inskeep [47] observed “intrusive materials”, including two silcrete micro-cores, within his Cutting 1 test pit. Deacon [10] found that typologically foreign (i.e non-Acheulian) intrusive artefacts were restricted to pothole fills within the Balmoral Member and some suggested intrusive elements within GBS. These consisted of “small silcrete elements, one triangular point and several fragments of snapped triangular or long quadrilateral flakes” [10 p110]. Deacon [10] suggested that their occurrence suggested that GBS provided a poor seal to the Acheulian bearing units prior to humification and compaction of organic material and GBS’ erosion by the unconformity. Deacon’s view of these as intrusive was also likely driven by the fact that Acheulian artefacts occurred in the Pothole Fill that followed the truncation of GBS. However, given the ~190 ka ages for the GBS, the GBS artefacts are probably not intrusive as suggested by Deacon [10] but simply represent MSA material in primary context. The Acheulian in the Pothole Fill (capped by a deposit dated to ~55 ka), where MSA elements also occur, are considered to have been deflated from the truncated underlying units. Similar intrusive MSA elements consisting of a number of Levallois flakes and snapped points occur in similar deposits in Area 2 [10 plate 52]. While apparently of low density, the MSA from GBS in Area 1 would be the oldest dated MSA along the southern Cape coast, slightly predating the oldest MSA deposits from Pinnacle Point Cave 13B between 174 and 153 ka [157, 158]. The dating of the stratified yellow sediments overlying the Pothole Fill to either 59–51 ka (weighted mean of OSL, TT-OSL and pIR-IR MAM-3 ages) or 93–79 ka (weighted mean of OSL and pIR-IR CAM ages) indicates that there was a significant period of erosion and lack of active deposition in the spring eye between the MIS 7 to 6 transition and either late MIS 5 or MIS 3. This age again indicates that the deposits are beyond the ^14^C age range and that the ^14^C age of ~32,900 BP obtained in the 1960s [10] is unreliable.

Micromorphological analysis indicates that the dark grey to black colour of some of the sediments is due to diffuse aggregates of amorphous organic matter occurring in all layers, though in very small quantity within the light grey layers. These are probably of vegetal origin, even if plant organs/tissues are rarely identifiable at the microscope scale, suggesting that the amorphous organic matter may derive from partial decomposition of phytoplankton. Relatively large and clearly identifiable vegetal remains are more common only in the Black Clay Bands. This aspect suggests that the initial stages of vegetal organic matter decay started elsewhere, probably in the more vegetated pool shore area, and that partly mineralised organic components were transported into more central parts of the basin. Relatively large vegetal remains are apparently more common in the BCB facies, which may represent a phase of swamp vegetation expansion, possibly connected to a decrease in water level and implying the migration of the marginal facies towards the centre of the pool. The texture of the observed sediments suggests that most sedimentary units were deposited by low-energy flow with moderate tractive currents, which occasionally produced discontinuously laminated sequences at the bottom of the basin. Short episodes of higher-energy flow are indicated by erosional features–occurring mostly during the sedimentation of light colour units–that reworked clods of the previously deposited black sediment, which can be found in some cases at the bottom of the light grey units. The contrast between the clods and the surrounding sediment, and the affinity with their source, are evident at the microscope scale, mostly reflected in different concentration of organic matter and sediment fabric. These reworked clods indicate that long-range (horizontal, as well as vertical) reworking did not occur, at least within the limited catchment of the excavation area. Low-energy flow or still water also fostered the establishing of anoxic conditions, preventing oxidisation of organic matter with consequent formation of black layers.

The pre-MIS 11 lower part (BCB1 and below; DHS and DBBCS, GGCS) of Deacon’s [10] Rietheuvel Member is archaeologically low density compared to the upper MIS 11 part (BHS, GCS) and MIS 7/6 (GBS) parts. This is due to the fact that there is a significant truncation of the lower units prior to the deposition of the Acheulian bearing BHS and GCS. While Deacon [10] suggests that there is lateral grading from more herbaceous sands in the southern part of Cutting 1 to more silts and clay rich deposits in the northern part, this is not actually the case in the upper part of the sequence. The lower units have in fact been truncated by the formation of a pool in the centre of the spring eye that was then filled with BHS. It appears from Deacon’s drawings that he identified such a feature between ~2.4 (8 ft) to ~3.0m (10ft) deep in the front of Cutting 1, and that this was associated with a dense occurrence of wood across Cutting 1 Extension and the southern part of Cutting 1. Deacon [10] considers this area to have been where ‘driftwood’ fragments came to rest at the edge of the spring pool. This wood clustering can clearly be identified as relating to the BHS.

**Fig 19:**
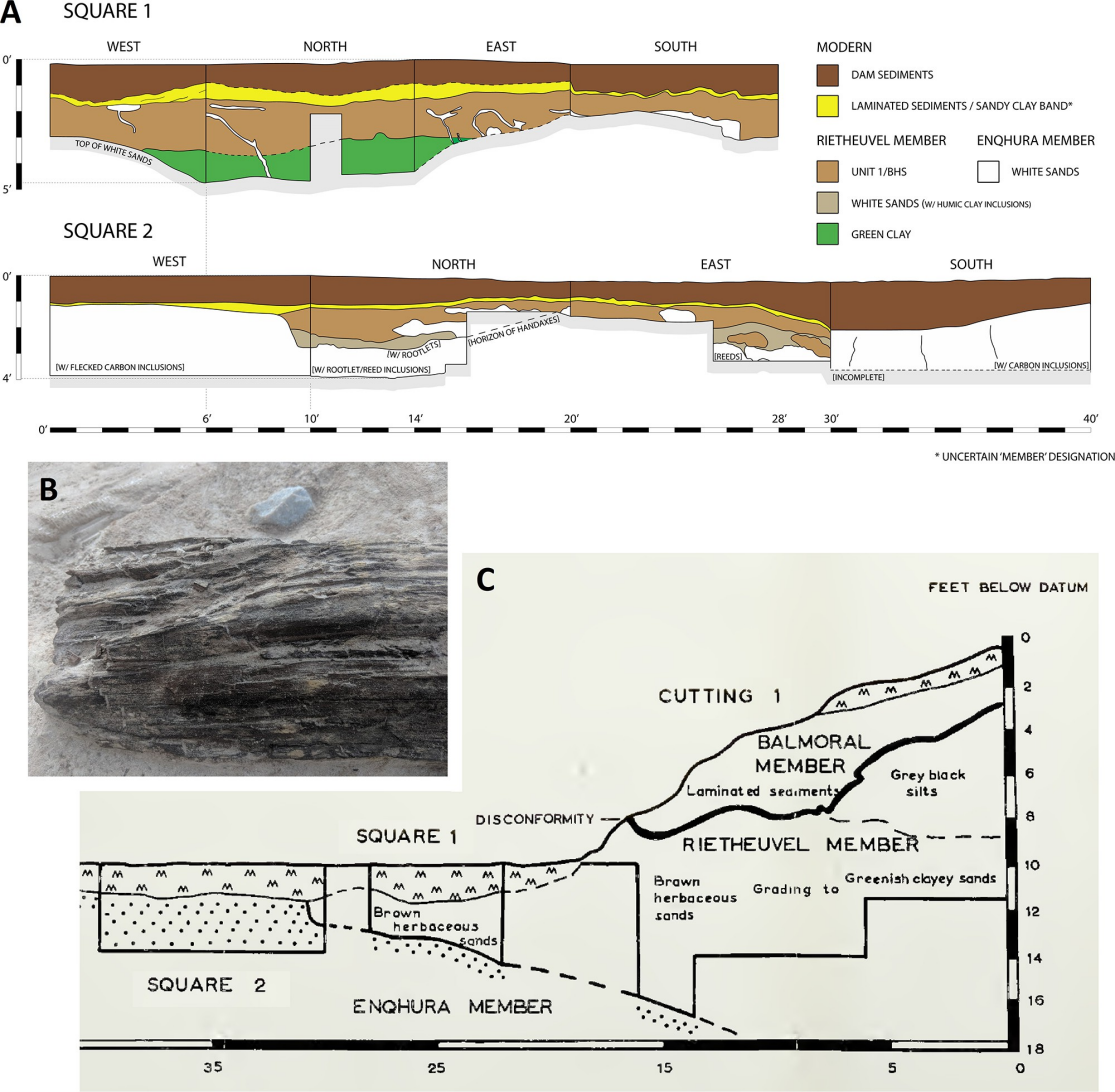
Stratigraphy of Area 1 Square 1 and 2 based on (A) Deacon’s redrawn excavation notes; (B) Wood and associated Acheulian artefacts from our 2019 excavations adjacent to Square 2 (C) Deacon’s [10] stratigraphic sections of Square 1 and 2.

The wood- and plant-rich BHS layers occur in a restricted area towards the western and central portion of the Area 1 spring (Cutting 1 extension and Cutting 1) and represent a period when the spring basin retained water but with marshy conditions and significant plant growth in its centre. Ground penetrating radar analysis confirms that the deepest deposits of Area 1 are in this westerly area of the spring and that the deposits become shallower to the east (see S1 File). This corresponds to the stratigraphic sequence becoming increasingly compressed, with some layers like BHS not represented, moving from west to east across the Cutting 1 and 10 excavation area. Deacon’s [10] western section drawing through Area 1 (Fig 19C) indicate that BHS extends significantly to the south of Cutting 1 and 10, here directly overlying the White Sands of the Enqhura Member and covered by recent sedimentation. Section drawings in Deacon’s unpublished notes (redrawn in Fig 19A) show that BHS is much more extensive, and that the stratigraphy is much more extensive in Square 1 and 2 than shown by sections shown in Deacon [10; Fig 19C]. Some layers such as the Green Clay of Square 1 have not been shown in the 1970 monograph. Test excavations into the centre of the spring eye in 2019, adjacent to Deacon’s Square 2, confirm the stratigraphy as shown in Deacon’s notes (Fig 19A). This test excavation yielded unworn Acheulian artefacts including extensive small flaking debris from BHS in direct association with wood (Fig 19B). The amount of small flaking debris in the BHS layers of this test excavation compared to the very small amount of <20mm artefacts in Deacon’s excavations (Fig 13) may suggest that either Deacon did not identify this size grade of stone artefacts in his excavations, or that the smaller material may have been winnowed further into the centre of the spring, leaving larger artefacts at the edge of the pool. The new ^14^C ages from BHS in our Cutting 1 Extension reveal that BHS wood samples from this area are older than 53 ka, indicating that the finite ^14^C age of 39,210–33,200 cal BP obtained in the 1970’s likely reflects insufficient removal of organic contaminants rather than the genuine occurrence of young, intrusive wood elements within the MIS 11 (~404 ka) BHS deposits. This context, as well as the luminescence and ^14^C dating indicate that the wood can be confidently associated with the Acheulian archaeology. Three prickle bases from the Deacon’s excavations of BHS in the Cutting 1 Extension are indistinguishable from *Erythrina* that are found growing in Eastern Cape today, although not in the area around Amanzi Springs or the immediate bushveld community of the area [57]. *E*. *caffra* is a constituent of the dry coastal forest into which the bushveld grades and may reflect a more seaward location of the springs during MIS 11, with the site being located within 7 km of the Swartkops Palaeoria (Fig 1; see below).

### The archaeology of Area 1

Regarding the integrity of the Area 1 lithic assemblage, the lack of knapping debris under 20 mm could be consistent with post-depositional winnowing, although recent test excavations into BHS in the central area of the spring have yielded fresh stone tools ranging from LCTs to small flakes and debris under 1cm. Therefore, size biasing in the Area 1 legacy collection may relate to a grading of smaller artefacts towards the centre of the pool. Alternatively, Deacon did not sieve the sediments and hired untrained excavators to aid his work, who may have not recognized diagnostic small flaking debris, which has confounded other archaeological assemblages [150]. However, while small flaking debris (<20 mm) represents only 4.2% of the total assemblage (Table 2), we note that larger materials (>20 mm) follow a distribution pattern similar to that reflected by on-site knapping [110].

Further, weathering patterns across the assemblage show that most artefacts are fresh (unabraded) or only slightly weathered. Inskeep [47] also recorded that 68% of his assemblage was fresh in condition, albeit his sample size was small (N = 128) by comparison. In contrast, 62% of the PHF artefacts are heavily abraded, further supporting the deflation scenario for their accumulation. It is worth pointing out that the spring sediments are highly abrasive because of their textural and lithologic characteristics. This may consequently have considerably affected the artefacts even under moderate transport, despite the hard raw material. Non-reworked artefacts may have also been abraded simply by particle-rich water flow with energies lower than artefact transport threshold. Amalgamating the evidence presented above, size profiles and fresh-to-slightly abraded weathering patterns, along with the micromorphological data, provide support that post-depositional disturbance was minimal within the BHS and GCS facies of Area 1. Therefore, we suggest that the Acheulian materials occur in a sealed, primary to near-primary context.

The assemblage itself reflects the production of both small flake tools and LCTs, albeit core sequences underlying small debitage manufacturing are seeming short. Unifacial and bifacial cores predominate, which show relatively low flake scar counts. Discoidal cores are disparate in terms of their rotation patterns and do not reflect high degrees of organized reduction patterns. Overall, core reduction was expedient in nature and focused predominantly on exploiting the longest flaking surfaces, rather than more systematic means of managing core volumes. This is reflected in the small number of prepared cores (n = 15), which are typically more abundant towards the end of the Acheulian [44, 159–161].

**Fig 20:**
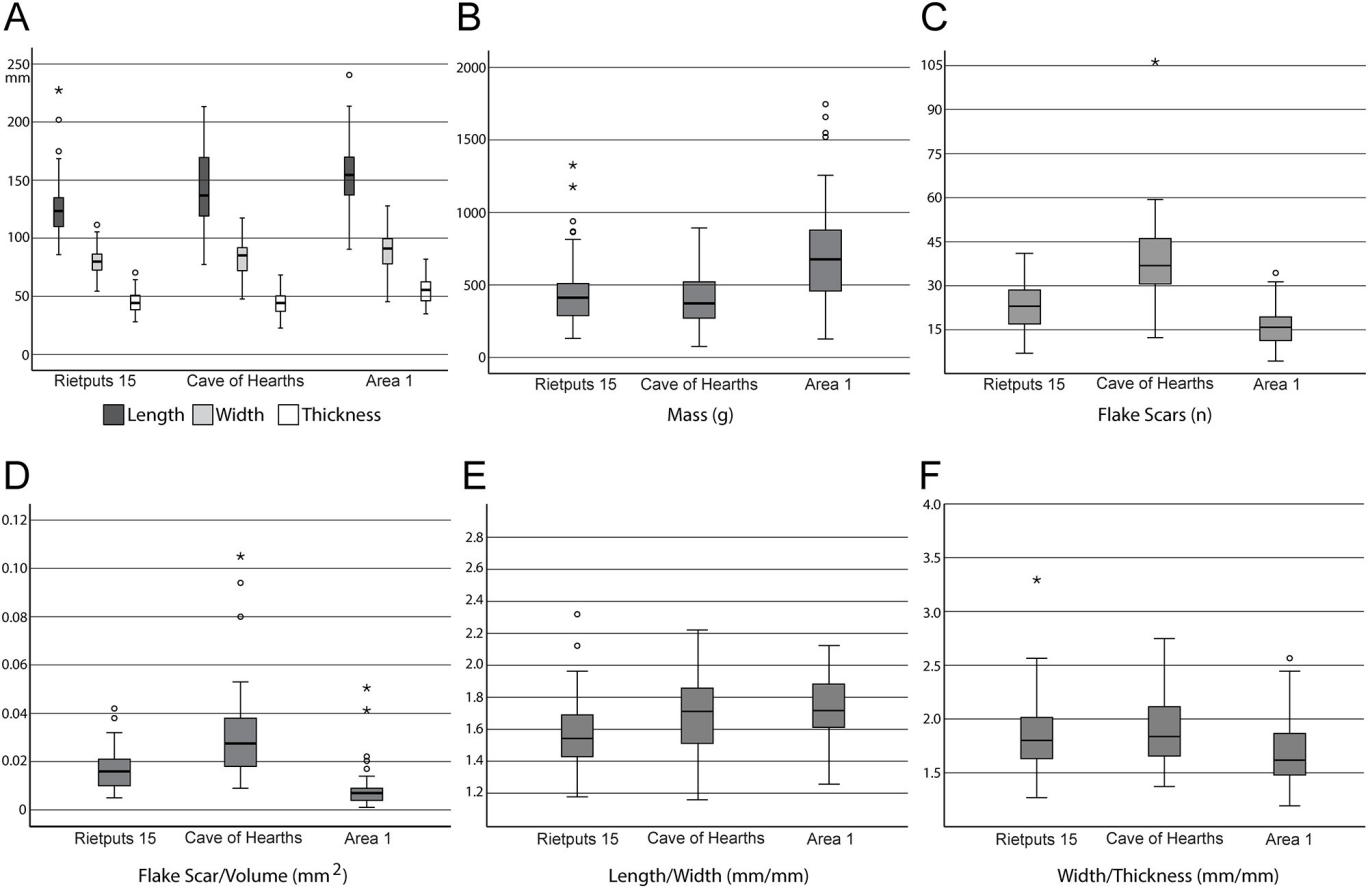
Comparison of Area 1 handaxes metrics with Rietputs 15 and Cave of Hearths. A) Boxplot of length, width and thickness variables; B) Boxplot of mass by grams; C) Boxplot of flake scar counts; D) Boxplot of scar density index by volume; E) Boxplot of elongation index; F) Box plot of refinement index.

Morphometric comparisons with Rietputs 15 and Cave of Hearths clarify the unique size and shape of the Area 1 handaxes. Fig 20 demonstrates that thickness and mass proportions are larger in the Amanzi Springs handaxes, which was confirmed by a Kruskal-Wallis test (thickness: x2 = 29.7, p< 0.001; mass: x2 = 34.3, p< 0.001), while flake scar counts are lowest (x2 = 95.1, p< 0.001). The SDI by volume comparisons showed that Amanzi Springs are significantly lower (x2 = 89.5, p< 0.001). Finally, the L/W (elongation) index showed differences between Amanzi Springs and Rietputs 15 (x2 = 17.9, p< 0.001), while W/Th (refinement) was lowest in the Area 1 sample (x2 = 17.0, p< 0.001).

In general, the Area 1 lithic materials do not match characteristic technological trends of the later Acheulian. With respect to the small debitage component, Area 1 lacks any MSA-like tools including blades and points, and contains a small proportion of prepared cores, which are common elements amongst other Late Acheulian and so-called Fauresmith localities [160, 162]. Instead Area 1 is typified by unifacial and bifacial cores and a small collection of retouched tools. Moreover, the LCTs demonstrate significant morphological variability when compared to other Acheulian assemblages (Fig 19) [13, 14, 153]. Deacon [10; p98] recognized such variation in describing the lithics from Amanzi Springs as “heavy and unstandardized”, as well as by including an ‘other biface’ category in his typological analysis. He noted morphological differences between the Amanzi Springs LCTs and other later Acheulian assemblages in South Africa, and suspected they could signal the beginning of a transition from the later Acheulian to the Sangoan industry, the latter characterized by the production of ‘crude’ LCTs forms and core axes [10; p117].

However, recent research has suggested that the overall size of LCTs from Amanzi Springs may actually relate to the function of the site as a workshop [13, 14, 153]. Sharon [153] found that the thickness and mass of handaxes and cleavers from Amanzi Springs were significantly larger when compared to 25 other Acheulian sites across western Asia and Africa. Caruana and Herries [13] further confirmed this trend in comparing handaxes from Amanzi Springs with earlier and later Acheulian assemblages from South Africa. Sharon [153] suggested the thickness of Amanzi Springs LCTs indicated a lack of shaping (*façonnage*) and resembled handaxes and cleavers from known workshop sites, including Isimila K19 (Tanzania) and STIC (Morrocco). He hypothesized that LCTs from these sites were akin to preforms that had not been extensively thinned or retouched.

The typological composition and technological features of the Area 1 assemblage also reflect on-site production of LCTs and further support a workshop hypothesis. The three giant cores reflect the early stages of LCT manufacturing at Area 1, presumably focused on defining handaxe tips. The *éclats de taille de biface* also document various stages of production. This includes three *éclats entame* (one used to shape a massive scraper) and other large flake blanks (used in heavy-duty activities) that were struck from giant cores, which further corroborate early stages of roughing out blanks or preparing a large flake removal (Fig 16) [121]. Shaping and thinning flakes, along with the 21 roughouts, represent preceding phases of *façonnage*. Together, these artefacts signify the by-products of manufacturing processes corresponding to Newcomer’s [163] ‘roughout’ and ‘thinning and shaping’ stages, or Callahan’s [164] ‘obtaining the blank’, ‘initial edging’ and ‘primary thinning’ stages. This provides valuable context for interpreting the unique proportions of handaxes and cleavers from Area 1 as ‘tools in production’ that were discarded before they were extensively thinned and retouched [13].

Therefore, we hypothesize that hominins visited Amanzi Springs to exploit the unique floral and faunal and resources sustained by the spring eyes, which included immediate access to useable toolstone. Acheulian toolmakers acquired raw material in the form of deflated Enon Formation quartzite cobbles and boulders that were preferable for both small debitage and LCT production, respectively. Handaxes and cleavers were shaped through the reduction of large cobbles and boulders or large flake blanks produced though core preparation methods including *entame* and slab-slice techniques [37, 152]. Another important aspect of site occupation is the thermal springs themselves, which recorded water temperatures of ~32° Celsius in the early 1900’s [10, 56]. It has recently been suggested that thermal waters at Oldupai Gorge ~1.7 million years ago may have been used by hominins to cook edible plants [165]. However, the iron-rich mineral content of the Amanzi Springs water is very different from those at Oldupai.

## Conclusion

The ~404–390 ka ages obtained for the BHS/GCS layers mean that Amanzi Springs Area 1 represents one of the few radiometrically dated Acheulian sites in South Africa. Moreover, it is the only known site across the region to contain Acheulian in direct association with significant wood remains. This, combined with the near primary context of the artefacts, means the site offers excellent insight to the technological practices of later Acheulian tool makers during a period when this industry was beginning to be augmented by tool forms that are characteristic of the MSA techno-complex in some areas of Africa [2, 22, 160, 166]. While the suspected later Acheulian is well-represented in South Africa at sites including Cape Hangklip, Canteen Kopje, Cave of Hearths, Duinefontein, Geelhoutboom, Kathu Pan, Montagu Cave, Muirton, Rooidam, Wonderboom; Wonderwerk Cave and the Zeekoe Valley [2, 22, 25, 27, 167, 168], the majority lack either definitive chronologies and/or in-depth artefact descriptions [although see 43]. There are thus no other sites confirmed to be the same age as Amanzi Springs Area 1 at ~404–390 ka and those that may be penecontemporaneous are largely confined to the interior highveld region. As such, characterising the technological milieu of the later and terminal phases of the Acheulian industry in South Africa remains somewhat problematic, albeit prevailing information suggests increased frequencies of prepared core technologies, retouched flakes, points, blades, as well as refined LCTs during the Middle Pleistocene [21]. Significantly, Amanzi Springs preserves a technological record that is quite different from these trends with large, unstandardized LCTs and low frequencies of prepared cores, retouched pieces and predetermined flake blanks.

The Area 1 BHS/GCS assemblage is dated to between the ‘Fauresmith’ layers (511–417 ka) and MSA (336–254 ka) layers of Kathu Pan [2, 22] and is younger than the later Acheulian (545–509 ka) with blades from the Kapthurin Formation [169]. It is also slightly older than MSA layers from excavation 2 at Wonderwerk Cave (~240–150 ka), and perhaps contemporary with some suggested Fauresmith Layers from excavation 6 at Wonderwerk Cave, although the age for these layers are uncertain [170]. The lack of any MSA elements in the Area 1 BHS/GCS assemblage may therefore indicate that there was variation in the adoption of these technologies across South Africa during the late Middle Pleistocene, with a potentially later occurrence of MSA technology along the coastal zone below the Great Escarpment. It maybe that these hominins preferred to make such MSA style small stone tool elements using finer-grained raw materials, and that due to Amanzi Springs being primarily a raw material source for quartzite that such tools are not represented at the site. At the new Area 7 site at Amanzi Springs there is a higher use of silcrete in the MSA, although the majority of the MSA at Amanzi is still made on quartzite. This suggests that if there were such MSA style elements in the Area 1 sequence they would likely be present. Together with the rich coastal MSA record (<~163 ka) of the southern Cape Coast, and older (>~650 ka) Acheulian record of the Sundays River [28], Amanzi Springs fills a gap between the last occurrence of the Acheulian and first occurrence of the MSA along the southern Cape Coastal zone. Further excavations of GBS, along with newly discovered MSA deposits at another spring (Area 7) at Amanzi Springs will help elucidate the oldest currently known occurrence of the MSA along the southern Cape coast, just prior to Pinnacle Point 13B at 163 ka, while the discovery of older Acheulian bearing deposits in deeper layers at Area 1 will push the timeline of the Acheulian at the site back further and create a regional sequence when compared with the late Early to early Middle Pleistocene sites along the Sundays River [28]. Work is ongoing to establish any behavioural relationship between the Acheulian technology and directly associated wood, which varies from large metre long in situ tree roots and large to small fragments.

## Supporting information

S1 File (DOCX)

S1 Data Supporting lithic analysis tables and data. (XLSX)
