# Supplementary material for: A marine isotope stage 11 coastal Acheulian workshop with associated wood at Amanzi Springs Area 1, South Africa
Source: PLoS One. 2022 Oct 20;17(10):e0273714. doi: 10.1371/journal.pone.0273714 (PMC9584507; doi:10.1371/journal.pone.0273714)
Supplement: S1 File — (DOCX) [file pone.0273714.s001.docx]

**S1. Supporting Information**

A marine isotope stage 11 coastal Acheulian workshop with associated wood at Amanzi Springs Area 1, South Africa

Andy I.R. Herries, Lee J. Arnold, Giovanni Boschian, Alex F. Blackwood, Coen Wilson, Tom Mallett, Brian Armstrong, Martina Demur, Fiona Petchey, Matthew Meredith-Williams, Paul Penzo-Kajewski, Matthew V. Caruana

# **Luminescence Dating Methods and Analyses**

**Equivalent dose (D_e_) determination**

K-feldspar pIR-IRSL and quartz OSL measurements were made on a Risø TL/OSL-DA-20 reader equipped with a ^90^Sr/^90^Y β radiation source and an Electron Tubes PDM 9107B photomultiplier tube. K-feldspar signals were stimulated using IR diodes (850 nm, maximum power of 340 mW/cm^2^) at 60% power and blue emissions were detected with a 2 mm-thick Schott BG39 and 3 mm-thick Schott BG3 filter pack. Single-grain quartz OSL signals were stimulated with a 10 mW Nd:YVO4 single-grain laser attachment emitting at 532 nm (maximum power of ~50 W cm^2^). Additional multiple-grain quartz OSL measurements (i.e., the OSL wash steps in **S1.1 Table**) were made using blue LEDs (470 nm, maximum power 102 mW/cm^2^) at 90% power. Quartz OSL emissions were detected in the ultraviolet region using a 7.5 mm-thick U340 filter. The mounted ^90^Sr/^90^Y beta source on the Risø TL/OSL-DA-20 reader had been calibrated to administer known doses to multiple-grain aliquots and single-grain discs. For single-grain measurements, spatial variations in beta dose rates across the disc plane were taken into account by undertaking hole-specific calibrations using gamma-irradiated quartz (Hansen et al., 2015).

Individual D_e_ values were only included in the final age calculation if they satisfied a series of quality-assurance criteria, as detailed in Demuro et al., (2015), Arnold et al., (2016) and Méndez-Quintas et al. (2018) (**S1.2 Table**). Single-grain OSL and TT-OSL D_e_ estimates were rejected from further consideration if they exhibited one or more of the following properties: (i) weak OSL signals (i.e., the net intensity of the natural test-dose signal, T_n_, was less than three times the standard deviation of the late-light background signal); (ii) poor recycling ratios (i.e., the ratios of sensitivity-corrected luminescence response (L_x_/T_x_) for two identical regenerative doses were not consistent with unity at 2σ); (iii) high levels of signal recuperation (i.e., the sensitivity-corrected luminescence response of the 0 Gy regenerative-dose point amounted to more than 5% of the sensitivity-corrected natural signal response (L_n_/T_n_) at 2σ); (iv) anomalous dose-response curves (i.e., those displaying a zero or negative response with increasing dose) or dose-response curves displaying very scattered L_x_/T_x_ values (i.e., those that could not be successfully fitted with the Monte Carlo procedure and, hence, did not yield finite D_e_ values and uncertainty ranges); (v) saturated or non-intersecting natural OSL signals (i.e., L_n_/T_n_ values equal to, or greater than, the *I_max_* saturation limit of the dose-response curve at 2σ); (vi) contamination by feldspar grains or inclusions (i.e., the ratio of the L_x_/T_x_ values obtained from two identical regenerative doses measured with and without prior IR stimulation (OSL IR depletion ratio; Duller, 2003) was less than unity at 2σ). Multiple-grain aliquot pIR-IRSL measurements were rejected from further D_e_ analysis if they failed the same quality assurance criteria, with the exception of criterion (vi).

Additionally, during analysis of the single-grain TT-OSL datasets it was found that a number of initially accepted grains displayed non-negligible, slow-decaying TT-OSL signals (i.e., their T_x_ signals did not reach background after 2 s of laser stimulation) (e.g., **Figure S1d**). Grains displaying such slow-dominated signals may not fulfil basic SAR suitability requirements (Wintle and Murray, 2006), and have been shown to be associated with thermally unstable signals, experimentally sensitised components or unreliable TT-OSL D_e_ estimates in several samples (e.g., Tsukamoto et al., 2008; Brown and Forman, 2012; Arnold and Demuro, 2015; Demuro et al., 2015; Bartz et al., 2019). Further examination showed that the signals of these grains did not originate from genuine thermal transfer of charge into the fast OSL trap, but rather they corresponded to interfering (non-transferred) slow OSL components from the previous OSL stimulation, which had not reached background levels prior to commencing the TT-OSL measurements. Following the findings of Bartz et al. (2019), we have used an additional Fast Ratio (FR) (Durcan and Duller, 2011) quality assurance criteria to ensure that potentially unsuitable grains displaying very slowly bleaching, non-transferred (interfering) OSL signals were not included in our TT-OSL age assessments. The FR has been calculated by comparing the counts in the initial channel (0.017 s) of the TT-OSL decay curve (L_1_) with those in the middle part of the decay (average counts over 1.0–1.2 s; L_2_) after subtracting a late light background count from the last 0.15 s (L_3_), according to the equation (L_1_-L_3_)/(L_2_-L_3_). The FR was calculated using the highest regenerative dose TT-OSL signal for each grain, in order to maximise signal-to-noise ratios and capture any progressive sensitisation or build-up of interfering, slowly bleaching OSL signals through the SAR procedure.

Sensitivity tests involving the application of increasingly stringent FR thresholds to the accepted TT-OSL D_e_ datasets (e.g., Bartz et al., 2019) reveal a noticeable influence on weighted mean D_e_ and overdispersion for the Area 1 samples (e.g., **Figure S2a-b**). In general, these samples show a ~20% increase in weighted mean D_e_ and a 30-40% decrease in overdispersion when applying incrementally higher FR acceptance thresholds between 0 and 20. Use of more stringent FR acceptance ratios >20 has no further discernible effect on D_e_ or overdispersion, other than causing a significant reduction in the number of accepted grains (**Figure S2a-b**). These trends are also reflected in the D_e_ distribution characteristics shown in **Figure S3**. Application of a FR acceptance threshold of ≥20 results in the elimination of several low, and often outlying, D_e_ values (particularly for samples ASP18-13 and ASP18-15), confirming they originate from grains with slowly decaying OSL signals that are poorly suited to being measured with the TT-OSL SAR protocol. From these sensitivity tests it appears that a FR acceptance threshold of ≥20 is suitable for eliminating any potentially biasing effects associated with slow decaying, interfering OSL signals for the Area 1 samples. As such, grains displaying a FR <20 were not included in the final D_e_ estimation, and they have been assigned to an additional TT-OSL rejection category.

The single-grain OSL and TT-OSL grain classification statistics obtained for each sample after applying these SAR quality assurance criteria are summarised in **Table S2**. 21% of the measured grains were considered suitable for OSL dating purposes, which reflects the relatively low proportion of non-OSL-producing grains for sample ASP18-16. All of the measured K-feldspar aliquots passed the SAR quality assurance criteria outlined above. 10-15% of the measured quartz grains were considered suitable for TT-OSL D_e_ evaluation, with 3-4% of grains being eliminated from the accepted D_e_ datasets because they exhibited very slow signal decay rates and FR<20.

**Figures S1a-c** show representative pIR-IRSL_250_, single-grain TT-OSL, and single-grain OSL decay and dose response curves for aliquots / grains that passed the SAR quality assurance criteria and were used for dating. The pIR-IRSL_250_ decay curves typically decrease by ~90% within the first 30 s of stimulation and are optimally fitted with a single saturating exponential plus linear function. All the D_e_ values were obtained from the region of the dose-response that was not in saturation when using this type of fitting function. The majority of accepted quartz grains display rapidly decaying TT-OSL and OSL curves (reaching background levels within 0.5 s), which are characteristic of quartz signals dominated by the most readily bleachable (so-called ‘fast’) OSL component **(Figure S1b** – compare OSL decay curve shape for a fast-dominated Risø calibration quartz grain; Hansen et al., 2015). The single-grain OSL dose-response curves are generally well-represented by either a single saturating exponential function or a saturating exponential plus linear function, as has been widely reported for single-grain quartz OSL signals (e.g., Yoshida et al., 2000; Jacobs et al., 2008; Arnold et al., 2011, 2016). The single-grain TT-OSL dose-response curves are all well-represented by a single saturating exponential function.

Individual D_e_ estimates are presented throughout this study with their 1σ error ranges, which are derived from three sources of uncertainty: (i) a random uncertainty term arising from photon counting statistics for each OSL measurement, calculated using Eq. 3 of Galbraith (2002); (ii) an empirically determined instrument reproducibility uncertainty of 0.6% for each multiple-grain aliquot measurement and 1.5% for each single-grain measurement (calculated for the specific Risø reader used for each sample according to the approach outlined in Jacobs et al., 2006a); and (iii) a dose-response curve fitting uncertainty determined using 1000 iterations of the Monte Carlo method described by Duller (2007) and implemented in Analyst.

**SAR D_e_ validation tests**

The pIR-IRSL_250_ SAR protocol shown in **Table S1** was initially chosen for the Area 1 samples on the basis of dose recovery tests performed on a sample from a closely related spring deposit (Area 7), which included examination of a range of preheat and pIR-IR stimulation temperatures. As part of the present study, we cross-checked the broader suitability of this pIR-IRSL_250_ SAR protocol for the Area 1 samples using a repeated dose recovery test on ASP18-13. For this purpose, six 400-grain K-feldspar aliquots were prepared and placed under direct sunlight for 8 hrs to bleach their naturally accumulated pIR-IRSL signals. These bleached aliquots were then split into two batches (*n* = 3 each), with the first batch being left un-dosed to determine the residual (unbleached) D_e_ remaining after daylight bleaching, and the second batch being given a laboratory dose of 650 Gy. The two batches of aliquots were then measured with the pIR-IRSL_250_ SAR protocol to determine their D_e_ values. Dose recovery (measured-to-given dose) ratios were calculated after subtracting the residual (unbleached) D_e_ of the un-dosed aliquots from the mean D_e_ obtained from the dosed aliquots. The residual-subtracted weighted mean measured-to-given dose ratio for the pIR-IRSL_250_ protocol overlaps with unity at 1σ (1.02 ± 0.02) and supports the suitability of the K-feldspar D_e_ determination procedure adopted in this study for the Area 1 samples. The weighted mean pIR-IRSL_250_ residual dose recorded for the three un-dosed aliquots of ASP18-13 after 8 h of daylight bleaching was 22.6 ± 2.3 Gy. Taking into consideration the limited bleaching time used in the dose recovery test, this relatively low residual D_e_ value provides reasonable support for the bleachability of the pIR-IRSL_250_ signals, and suggest that any thermal transfer of charge during preheating (e.g., Buylaert et al.*,* 2011; Qin and Zhou, 2012) or photo-transfer of electrons to low temperature TL traps during prior IR stimulation (e.g., Wang and Wintle, 2013) is not likely to contribute significantly to the natural D_e_ values of these samples.

Single-grain OSL and TT-OSL dose recovery tests were undertaken on samples ASP18-16 and ASP18-13, respectively, to assess the suitability of the chosen SAR protocols shown in **Table S1**. The single-grain TT-OSL SAR protocol adopted in this study follows that reliably employed at several independently or semi-independently dated Middle Pleistocene archaeological sequences recently (e.g., Arnold et al., 2014; Demuro et al., 2015, 2019; Ollé et al., 2016; Bartz et al., 2019). Single-grain TT-OSL dose recovery tests were performed on ASP18-13 by adding a dose of 359 Gy on top of the naturally accumulated dose for a subset of 800 grains. This approach was adopted owing to the long durations of light exposure needed to bleach natural TT-OSL signals down to low residual levels (e.g., Demuro et al., 2015; Arnold et al., 2019). The recovered dose ratio was then calculated by subtracting the weighted mean natural TT-OSL D_e_ of sample ASP18-13 (i.e., 587 ± 26 Gy, as shown in **Table 1** and determined from 1000 grains) from the weighted mean D_e_ of the unbleached and dosed grains (i.e., 939 ± 41 Gy; **Figure S4b**).

The single-grain TT-OSL dose recovery test results obtained for ASP18-13 support the use of an additional FR acceptance threshold of ≥20 for final age determination. The plot of FR acceptance threshold versus weighted mean D_e_ / overdispersion for the unbleached and dosed grains mirrors that obtained for the natural D_e_ dataset of ASP18-13 (**Figure S4c**). Individual grains exhibiting FR ratios <20 also appear to form a distinct clustering of low D_e_ values on the radial plot for the unbleached and dosed grains (**Figure S4b**). A net (i.e., natural-subtracted) weighted mean TT-OSL measured-to-given dose ratio of 0.98 ± 0.06 (overlapping with unity at 1σ) was obtained for this sample when applying a FR acceptance threshold of ≥20.

Single-grain OSL dose recovery test measurements were made after administering a dose of 100 Gy to grains that had previously been bleached with two 50°C blue LED exposures of 1000 s, separated by a 10,000 s pause. Regenerative dose and test dose preheat treatments of 240°C for 10 s and 200°C for 10 s, respectively, were used in the single-grain OSL SAR protocol, as these conditions were determined to be optimal for samples from adjacent spring deposits. The single-grain OSL dose recovery test yielded a mean measured-to-given dose ratio of 1.00 ± 0.02 and an overdispersion value of 13 ± 2%, confirming the suitability of the chosen preheat combination for the Area 1 deposits (**Figure S4a**).

It is worth noting that it is not possible to accurately recover a known laboratory dose using these optimum single-grain OSL measurement conditions unless we apply the SAR quality assurance criteria described earlier. This issue was examined by reanalysing the single-grain OSL dose recovery dataset after omitting as many of the SAR quality assurance criteria as practically possible; i.e. eliminating quality assurance criteria (ii), (iii), (iv) and (vi), and accepting all measured grains that have statistically significant OSL signals (T_n_ signal > 3 s.d. background) and that yield monotonic dose-response curves permitting finite D_e_ interpolation. In comparison with the original dose recovery results (i.e. those obtained using the full suite of SAR quality assurance criteria), the resultant weighted mean measured-to-given dose ratio decreases by 9% and does not overlap with unity at 2σ (weighted mean measured-to-given dose ratio = 0.91 ± 0.02), while the overdispersion increases from 13 ± 2% to 19 ± 2%. Similar statistically significant D_e_ and overdispersion offsets are apparent for the natural D_e_ dataset of ASP18-16 when considering all measured grains without applying any quality assurance criteria; the weighted mean natural D_e_ value of ASP18-16 decreases from 114 ± 6 Gy (Table 1) to 84 ± 6 Gy, while the overdispersion increases from 51 ± 4% to 90 ± 5%.

The results of these sensitivity tests confirm that inclusion of SAR quality assurance criteria is necessary for reliable OSL D_e_ determination at this site, as well as for minimising intrinsic D_e_ scatter arising from unsuitable grain types that do not uphold the fundamental principles of the SAR procedure (i.e. those affected by inadequate sensitivity correction, significant charge transfer between SAR cycles, contamination by non-quartz luminescence signals, significant contributions from non-fast OSL signals, and non-reproducible / non-monotonic dose-response characteristics that are not well-represented by single-saturating exponential or exponential plus linear fitting functions). Indirectly, these results also highlight the importance of undertaking single-grain rather than multi-grain OSL measurements at this site. While application of the SAR rejection criteria enables these non-trivial populations of unreliable grains to be individually removed from the single-grain OSL D_e_ dataset, it would not be possible to eliminate these unsuitable grain types from any multi-grain OSL analysis performed on ASP18-16. These aberrant grain populations would therefore contribute to the average light sums of the resultant multi-grain D_e_ measurements and could potentially give rise to the types of systematic multi-grain OSL biasing effects that have been documented for some quartz samples (e.g., Jacobs et al., 2006a; Demuro et al., 2008, 2013; Arnold et al., 2012b, 2013; Stone and Bailey, 2012; Russell and Armitage, 2012). Indeed, comparison of the ‘synthetic aliquot’ results confirm that this is the case for ASP18-16, with the summed OSL signals of all grains contained on each single-grain disc yielding statistically significant D_e_ offsets for the dose recovery dataset of this sample (weighted mean measured-to-given dose ratio = 0.92 ± 0.02; *n*=3 synthetic aliquots).

**Environmental dose rate estimation**

**Table 1** summarises the dose rate estimates for the Area 1 luminescence dating samples, which were made using a combination of *in situ* gamma spectrometry measurements and low-level beta counting. Field gamma spectrometry measurements were performed at each luminescence dating sample position immediately after removal of the PVC tubes. Elemental concentrations of K, U and Th were determined from the field gamma-ray spectra using the ‘energy windows’ method described in Arnold et al. (2012a) and Duval and Arnold (2013). These elemental concentrations were then used to calculate the gamma dose rates, enabling us to capture any spatial heterogeneity in the surrounding (~30 cm diameter) gamma radiation field of each sample. External beta dose rates were determined from measurements made using a Risø GM-25-5 beta counter (Bøtter-Jensen and Mejdahl, 1988) on dried and homogenised, bulk sediments collected directly from the luminescence dating sampling positions. This approach was used to ensure that beta dose rates were derived from sample sizes that closely approximate the very short (~2-3 mm) beta particle radiation fields affecting these samples. Background-subtracted count rates were measured for three aliquots of each sample and compared with net count rates obtained simultaneously for a loess sediment standard with known U, Th and K concentrations (Potts et al., 2003). Cosmic-ray dose rates were calculated using the approach described in Prescott and Hutton (1994). A small, assumed internal (alpha plus beta) dose rate of 0.03 ± 0.01 Gy / ka has been included in the final dose rate calculations for the quartz fractions of these samples, based on published ^238^U and ^232^Th measurements for etched quartz grains from a range of locations (e.g., Mejdahl, 1987; Bowler et al., 2003; Jacobs et al.*,* 2006b; Pawley et al., 2008; Lewis et al 2020) and an alpha efficiency factor (*a*-value) of 0.04 ± 0.01 (Rees-Jones, 1995; Rees-Jones and Tite, 1997). Internal alpha and beta dose rate contributions for K-feldspar grains have also been calculated using previously published values for intrinsic ^238^U, ^232^Th, ^40^K and ^87^Rb contents. Internal ^40^K and ^87^Rb concentrations were assumed to be 12.5 ± 0.5% (Huntley and Baril, 1997) and 400 ± 100 ppm (Huntley and Hancock, 2001), respectively. Internal ^238^U and ^232^Th concentrations were assumed to be 0.15 ± 0.03 ppm and 0.35 ± 0.07 ppm, respectively, based on modal values obtained by Mejdahl (1987) and similar values obtained by Huntley and Clague (1996), Huntley and Lian (1999), and Alappat et al. (2010). An *a*-value of 0.09 ± 0.03 was used to estimate the internal alpha dose rate contributions from these ^238^U and ^232^Th concentrations, based on published estimates obtained for a range of K-feldspar samples (e.g., Rees-Jones, 1995; Lang and Wagner, 1997; Banerjee et al.*,* 2001; Lang et al.*,* 2003; Berger et al.*,* 2008; Feathers et al.*,* 2012).

Radionuclide concentrations and specific activities have been converted to dose rates using the conversion factors given in Readhead (2002) and Guérin et al. (2011) (see Table 1 footnotes for details), making allowance for beta-dose attenuation (Mejdahl, 1979; Brennan, 2003) and long-term sediment water contents (Aitken, 1985; Readhead, 1987). The present-day sediment water contents measured for the Area 1 samples, which ranged between 5 and 21% of dry sediment weight, are not considered to be entirely representative of those prevailing throughout the long-term burial periods because: (i) some of the excavation pits and sediment exposures had partially dried out prior to sampling, (ii) the luminescence dating samples were collected during the dry-season, and (iii) the present-day water table level at Amanzi Springs is artificially lower than the long-term average level owing to agricultural drainage and groundwater exploitation activities. To determine more suitable long-term sediment moisture contents, we examined the range of present-day ‘proportional saturated water content’ values (i.e., present-day water contents / saturated water contents x 100; Aitken, 1998) obtained for the four luminescence samples. The highest proportional saturated water content for these samples (55% of saturated water content = sample ASP18-15) was obtained from a freshly dug excavation trench in the central area of Cutting 10, and was used to establish a more reliable estimate of present-day water content (as a proportion of sediment saturation capacity) in the absence of significant desiccation effects prior to sampling. Based on these results, and factoring in the potential for higher long-term groundwater levels, as well as intermittent surface flooding and reactivation of the spring eye during past periods of higher sea level (as indicated by the micromorphology analysis), we have adopted conservative long-term sediment moisture contents of 70% (instead of 55%) present-day saturated water contents for each luminescence dating sample. Using this approach, the long-term sediment moisture contents for the Area 1 luminescence dating samples range between 18 and 27 % of dry sediment weight (**Table 1**), and have been assigned a fixed 1σ uncertainty of ±5 % (±10% at 2σ) to accommodate any variations in hydrologic conditions during burial. These assigned 2σ uncertainty ranges ensure that the long-term sediment moisture contents span all reasonable possible hydrological scenarios between the minimum and maximum end-member limits (i.e., between the measured present-day water content and the measured saturated water content for each sample, respectively).

Whilst 70% of the saturated water content has been chosen as the best estimate of long-term water content for these samples, it is worth noting that use of a mean value closer to the measured present-day water content of ASP18-15 (i.e., the sample with the most secure proportional saturated water content estimate) would not alter the final age estimates beyond the existing uncertainty ranges. For example, use of long-term sediment moisture contents of 60% present-day saturated values (i.e., closer to the 55% of saturated water content measured for ASP18-15) would cause the final OSL and TT-OSL ages of these four samples to decrease by 1.5–13.3 kyr, with the corresponding pIR-IRSL ages decreasing by 1.0–10.6 kyr. In all case, the resultant ages are not statistically different at 2σ from those shown for each sample in **Table 1**, and therefore our luminescence chronologies and interpretations are considered relatively insensitive to the preferred choice of long-term water content.

| **Step** | **pIR-IRSL_250_ SAR** | **Signal** | **Step** | **Single-grain TT-OSL SAR** | **Signal** | **Step** | **Single-grain OSL SAR** | **Signal** |  |
| --- | --- | --- | --- | --- | --- | --- | --- | --- | --- |
| 1 | Dose (natural or laboratory) |  | 1 | Dose (natural or laboratory) |  | 1 | Dose (natural or laboratory) |  |  |
| 2 | Preheat 1 (280ºC for 60 s) |  | 2 | Preheat 1 (260ºC for 10 s) |  | 2^a^ | IR stimulation (50ºC for 60 s) |  |  |
| 3 | IR stimulation (50ºC for 200 s) |  | 3 | SG OSL stimulation (green laser; 125ºC for 2 s) |  | 3 | Preheat 1 (240ºC for 10 s) |  |  |
| 4 | pIR-IR stimulation (250ºC for 200 s) | L_x_ or L_n_ | 4 | Preheat 2 (260ºC for 10 s) |  | 4 | SG OSL stimulation (green laser; 125ºC for 2 s) | L_x_ or L_n_ |  |
| 5 | Test dose (200 Gy) |  | 5 | SG OSL stimulation (green laser; 125ºC for 3 s) | L_x_ or L_n_ | 5 | Test dose (15 Gy) |  |  |
| 6 | Preheat 2 (280ºC for 60 s) |  | 6 | OSL wash (blue LEDS; 280ºC for 400 s) |  | 6 | Preheat 2 (200ºC for 10 s) |  |  |
| 7 | IR stimulation (50ºC for 200 s) |  | 7 | Test dose (200 Gy) |  | 7 | SG OSL stimulation (green laser; 125ºC for 2 s) | T_n_ or T_x_ |  |
| 8 | pIR-IR stimulation (250ºC for 200 s) | T_n_ or T_x_ | 8 | Preheat 3 (260ºC for 10 s) |  | 8 | Repeat measurement cycle for different sized |  |  |
| 9 | IR wash (at 290ºC for 100 s) |  | 9 | SG OSL stimulation (green laser; 125ºC for 2 s) |  |  | regenerative doses |  |  |
| 10 | Repeat measurement cycle for |  | 10 | Preheat 4 (260ºC for 10 s) |  |  |  |  |  |
|  | different sized regenerative doses |  | 11 | SG OSL stimulation (green laser; 125ºC for 3 s) | T_n_ or T_x_ |  |  |  |  |
|  |  |  | 12 | OSL wash (blue LEDS; 290ºC for 400 s) |  |  |  |  |  |
|  |  |  | 13 | Repeat measurement cycle for different sized |  |  |  |  |  |
|  |  |  |  | regenerative doses |  |  |  |  |  |

^a^ Step 2 is only included in the single-grain OSL SAR procedure when measuring the OSL IR depletion ratio (Duller, 2003).

**S1.1 Table.** Single-aliquot regenerative-dose (SAR) protocols used to measure multiple-grain K-feldspar pIR-IRSL_250_, single-grain TT-OSL, and single-grain OSL D_e_ values. Each SAR measurement cycle was repeated for the natural dose, three to five different sized regenerative doses, a 0 Gy regenerative-dose (to measure signal recuperation) and a replicate of the first regenerative-dose cycle (to assess the suitability of the test-dose sensitivity correction). In the case of the single-grain OSL SAR procedure, both the smallest and largest non-zero Gy regenerative dose cycles were repeated at the end of the SAR procedure to assess the suitability of the test dose sensitivity correction over different dose ranges. The smallest regenerative-dose cycle was then repeated a second time with the inclusion of step 2 to check for the presence of feldspar contaminants using the OSL IR depletion ratio of Duller (2003). L_x_ = regenerative dose signal response; L_n_ = natural dose signal response; T_x_ = test dose signal response for a laboratory dose cycle; T_n_ = test dose signal response for the natural dose cycle.

| **Sample name** | **ASP18-16** | **ASP18-16** | **ASP18-16** | **ASP18-14** | **ASP18-15** | **ASP18-13** | **ASP18-13** |
| --- | --- | --- | --- | --- | --- | --- | --- |
| **SAR measurement type** | TT-OSL D_e_ | OSL D_e_ | OSL  Dose-recovery | TT-OSL D_e_ | TT-OSL D_e_ | TT-OSL D_e_ | TT-OSL  Dose-recovery |
| **Total measured grains (*n*)** | 1000 | 500 | 300 | 900 | 1000 | 1000 | 800 |
| **Grains rejected for failing SAR quality assurance criteria (%)** |  |  |  |  |  |  |  |
| T_n_ <3*σ* background | 60 | 27 | 26 | 44 | 66 | 66 | 64 |
| Low-dose recycling ratio ≠ 1 at ±2*σ* | 4 | 10 | 8 | 7 | 5 | 4 | 4 |
| High-dose recycling ratio ≠ 1 at ±2*σ* | - | 7 | 10 | - | - | - | - |
| OSL-IR depletion ratio <1 at ±2σ | 0 | 3 | 4 | 0 | 0 | 0 | 0 |
| 0 Gy L_x_/T_x_ >5% L_n_/T_n_ | 0 | 5 | 1 | 0 | 0 | <1 | 0 |
| Non-intersecting grains (L_n_/T_n_ > dose response curve saturation) | 0 | 1 | 0 | <1 | <1 | <1 | 0 |
| Saturated grains (L_n_/T_n_ ≥ dose response curve *I_max_* at ±2σ) | 0 | 0 | <1 | 0 | 0 | 0 | 0 |
| Anomalous dose response / unable to perform Monte Carlo fit | 23 | 26 | 21 | 30 | 16 | 17 | 20 |
| Slowly decaying signals ( L_x_ Fast Ratio <20) | 3 | - | - | 3 | 4 | 3 | 3 |
| **Sum of rejected grains (%)** | 90 | 79 | 70 | 85 | 91 | 90 | 91 |
| **Sum of accepted grains (%)** | 10 | 21 | 30 | 15 | 10 | 10 | 9 |

**S1.2 Table** Single-grain TT-OSL and OSL classification statistics for the dose recovery and natural D_e_ measurements of the Area 1 luminescence samples. The proportion of grains that were rejected from final D_e_ estimation after applying the various SAR quality assurance criteria are shown in rows 5-13. These criteria were applied to each single-grain measurement in the order listed. T_n_ = natural test dose signal response; L_n_/T_n_ = sensitivity-corrected natural signal response; L_x_/T_x_ = sensitivity-corrected regenerative-dose signal response; *I_max_* = saturation OSL intensity of the fitted dose response curve.

| **Sample** | **Fading rate (*g_2days_*)**  **(% / decade) ^a^** | **Uncorrected age**  **(ka) ^b, c^** | **Fading-corrected**  **age (ka) ^b, c^** |
| --- | --- | --- | --- |
| ASP18-16 | 1.14 ± 0.22 | 54.3 ± 4.8 (MAM-3) | 60.3 ± 5.5 (MAM-3) |
|  |  | 86.8 ± 9.2 (CAM) | 96.5 ± 10.5 (CAM) |
| ASP18-14 | 1.52 ± 0.04 | 174.9 ± 12.0 (CAM) | 202.4 ± 13.9 (CAM) |
| ASP18-15 | 1.54 ± 0.29 | 389.6 ± 24.5 (CAM) | 452.6 ± 31.8 (CAM) |
| ASP18-13 | 1.47 ± 0.11 | 403.9 ± 23.4 (CAM) | 464.3 ± 27.4 (CAM) |

^a^ Laboratory fading rates were measured following the procedure suggested by Auclair et al. (2003). The *g*-values were determined from repeated L_x_/T_x_ measurements made after different storage times (ranging from 0.2 h to 30 h) using Eq. 4 of Huntley and Lamothe (2001), and have been normalised to a measurement delay time of two days (*g_2days_*) to enable direct comparisons with published values.

^b^ The final pIR-IRSL_250_ ages have been calculated without applying an additional empirical fading correction owing to the low g-values recorded for these samples (see main text for details). Fading corrected pIR-IRSL_250_ ages are presented in the final column for comparative purposes only. CAM = central age model; MAM-3 = three-parameter minimum age model.

^c^ Mean ± total uncertainty (68% confidence interval), calculated as the quadratic sum of the random and systematic uncertainties. Total uncertainty includes a systematic component of ± 2% associated with laboratory beta-source calibration.

**S1.3 Table** K-feldspar pIR-IRSL_250_ empirical fading rates (g-values), uncorrected ages and fading-corrected ages for the Area 1 luminescence samples.

**
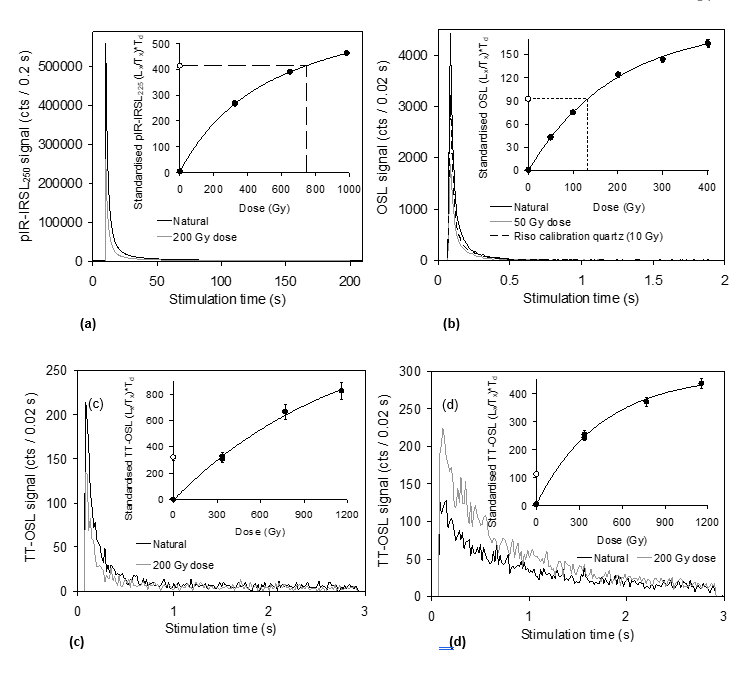
**

_250_

**S1.1 Fig** **Representative K-feldspar pIR-IRSL_250_, single-grain OSL and single-grain TT-OSL decay / dose-response curves for the Area 1 luminescence dating samples**. In the insets, the open circle denotes the sensitivity-corrected natural signal, and filled circles denote the sensitivity-corrected regenerative dose signals. (a) ~400-grain K-feldspar aliquot of sample ASP18-13. (b) Individual quartz grain from ASP18-16 with relatively bright OSL signal, typical decay shape and saturating exponential dose-response curve function. The OSL decay curve of a fast-component dominated calibration quartz grain is shown in this plot for comparison (Risø calibration quartz from Rømø, batch #98; Hansen et al., 2015). (c) Individual quartz grain from sample ASP18-14 with a moderately bright TT-OSL signal, typical decay shape, saturating exponential dose-response curve function, and a moderate Fast Ratio of 54. (d) Individual quartz grain from sample ASP18-15 with a moderately bright TT-OSL signal, very slow decay shape, saturating exponential dose-response curve, and a low Fast Ratio of 6.

**
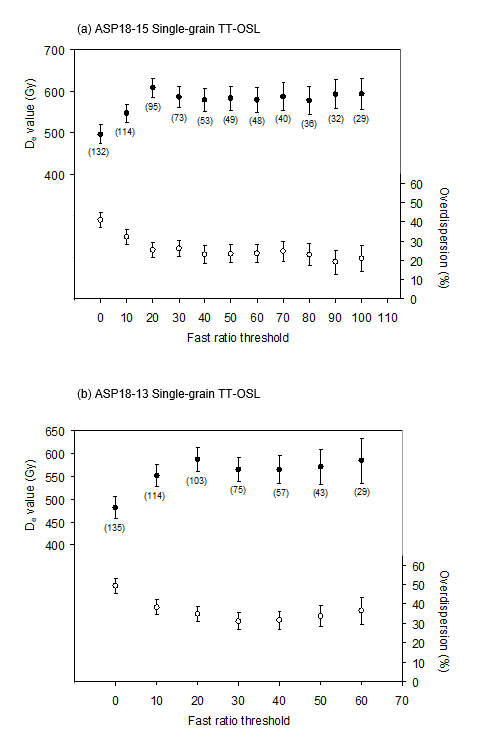
**

**S.1.2 Fig** **Plots showing the weighted mean (CAM) D_e_ and overdispersion values obtained for sample ASP18-13 and ASP18-15 when applying different Fast Ratio (FR) thresholds.** In these plots, progressively higher FR thresholds have been applied to the D_e_ datasets, starting with a FR threshold of 0 and increasing the FR threshold in increments of 10 until the culled dataset contained fewer than 30 individual D_e_ values (i.e., the sample size became too limited to ensure precise single-grain D_e_ determination). In each instance, grains were only accepted for further D_e_ analysis if their individual FR value equalled or exceeded the corresponding threshold shown on the x-axis. The values shown in brackets represent the number of grains remaining in the D_e_ dataset after applying each Fast Ratio threshold criterion.

**
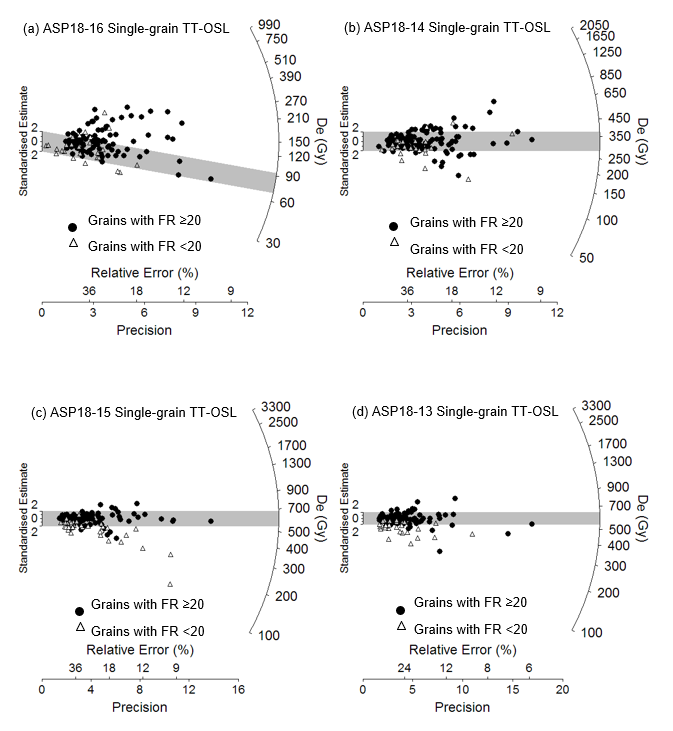
**

**S1.3** **Fig Comparison of single-grain TT-OSL D_e_ datasets derived using accepted grains with Fast Ratios (FR) <20 and accepted grains with FR ≥20 for each of the four Area 1 samples**. The grey bands are centred on the MAM-3 (ASP18-16) or CAM (ASP18-13, ASP18-14, ASP18-15) D_e_ values obtained using accepted grains with FR ≥20.

**
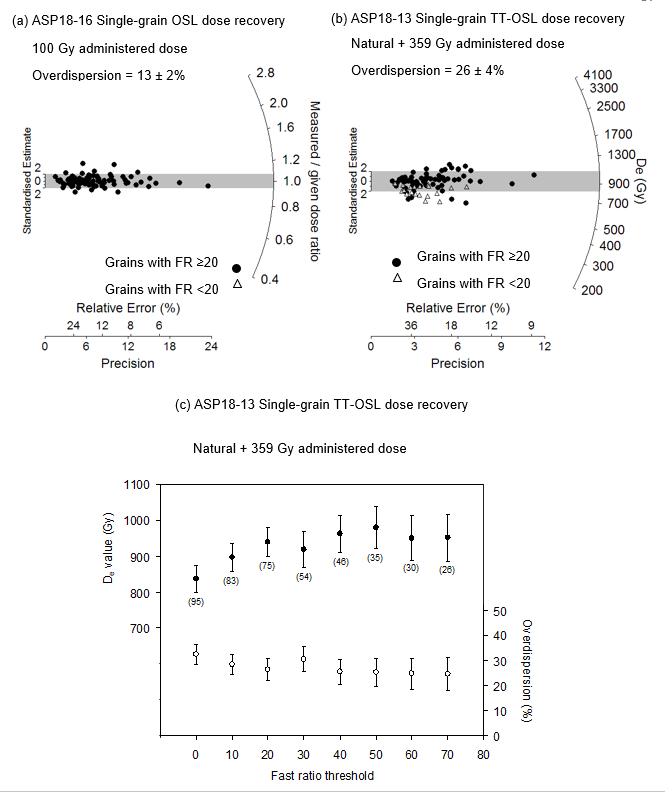
**

**S1.4 Fig Single-grain OSL and single-grain TT-OSL dose-recovery test results.** (a) Radial plot showing the measured-to-given dose OSL ratios for individual quartz grains of sample ASP18-16 using a regenerative dose preheat of 240 ^o^C for 10 s and a test dose preheat of 200 ^o^C for 10 s. The grey shaded region on the radial plot is centred on the administered dose for each grain (sample average ~100 Gy). Individual D_e_ values that fall within the shaded region are consistent with the administered dose at 2σ. (b) Radial plot showing the dose-recovery test (natural + dosed) TT-OSL D_e_ values obtained for sample ASP18-13 after applying the routine SAR quality assurance criteria (filled circles and open triangles together) and after applying an additional Fast Ratio threshold criterion (determined using the results shown in **Figure S2**) (filled circles only). The grey band is centred on the CAM D_e_ value obtained using accepted grains with FR ≥20. (c) Plot showing the weighted mean (CAM) D_e_ and overdispersion values obtained for the ASP18-13 TT-OSL dose-recovery dataset when applying different Fast Ratio thresholds. See **Figure S2** caption for further details.


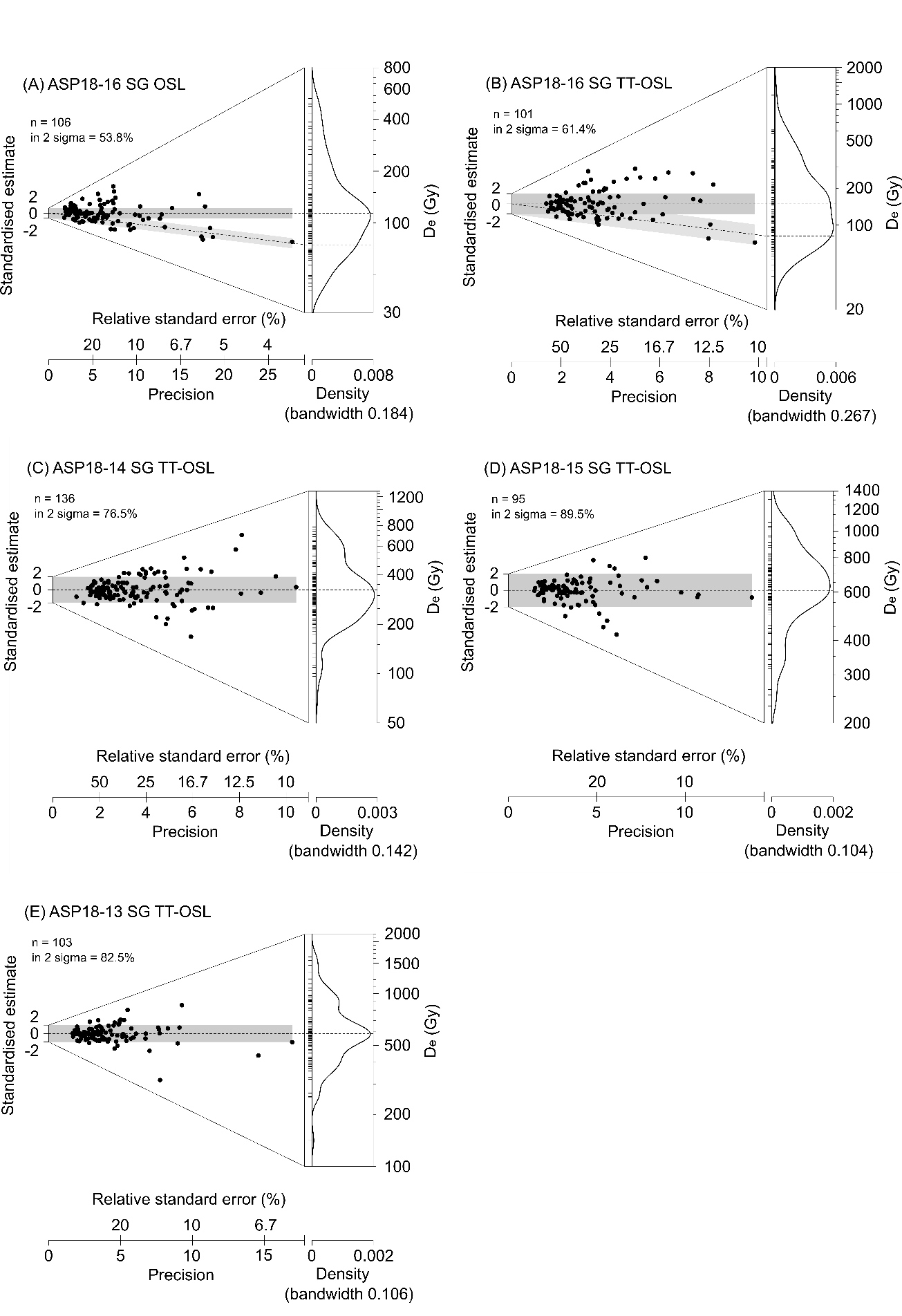


**S1.5 Fig Single-grain OSL and TT-OSL D_e_ distributions for the Area 1 luminescence dating samples**, shown as abanico plots. The shaded bands are centred on the D_e_ values used for the age calculations, which were derived using either the 3-parameter minimum age model (light grey band sample ASP18-16) or the central age model (dark grey bands samples ASP18-14, ASP18-15, ASP18-13, ASP18-16) of Galbraith et al. (1999).

**
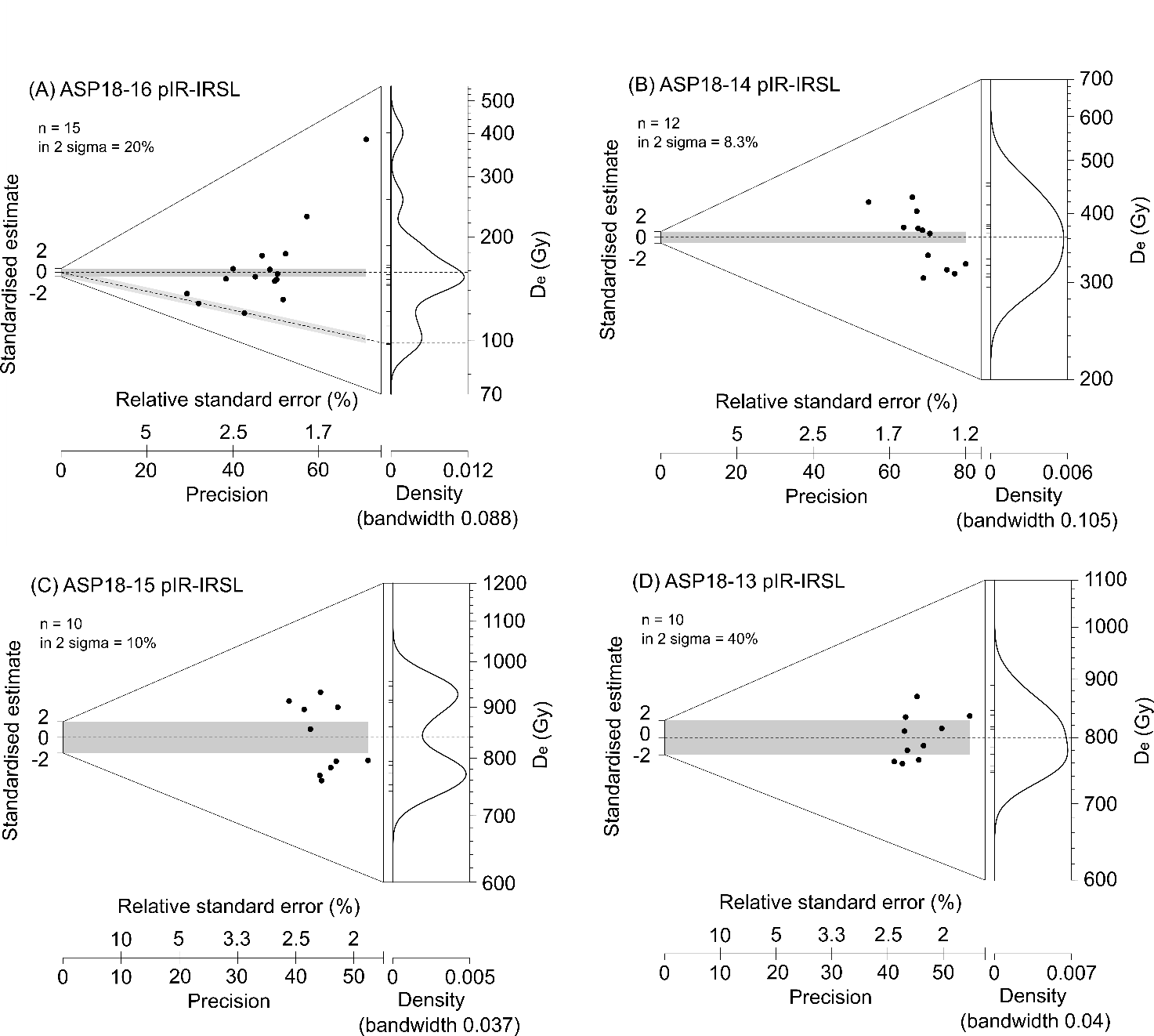
**

**S1.6 Fig Multi-grain pIR-IRSL D_e_ distributions for the Area 1 luminescence dating samples**, shown as abanico plots. The shaded bands are centred on the D_e_ values used for the age calculations, which were derived using either the 3-parameter minimum age model (light grey band sample ASP18-16) or the central age model (dark grey bands samples ASP18-14, ASP18-15, ASP18-13, ASP18-16) of Galbraith et al. (1999).

# **Ground Penetrating Radar**

Ground Penetrating Radar (GPR) survey was conducted at Area 1 to test the applicability of the technique to identify the depth of spring deposits and any subsurface anomalies, prior to our renewed excavations. A MALÅ ProEx GPR unit was used with a 50MHz unshielded and a 250MHz shielded antenna. A total of eleven survey runs were conducted across Area 1, three with the 50MHz antenna (Runs 0288-0290) and eight with the 250MHz (Runs 0291-0299) (Figure S5A). At the time of fieldwork, dense vegetation across the spring prevented the use of a systematic parallel survey grid and limited the survey to areas with suitable ground coverage. The start and end locations of the survey runs were recorded by a differential global positioning system (dGPS) to sub centimetre accuracy.

Processing of the radargrams was conducted on Mala’s Object Mapper (Ver 2.0.1), using processing routines including time-zero correction, AGC gain, and hyperbola fitting for velocity analysis. Bandpass filtering was conducted at a later stage to remove vertical banding in the radargram outputs. Radargram 0291 is presented in Figure S5B, showing a traverse southwest to northeast transect across Area 1 with two main features of interest. Firstly, vertical banding can be seen throughout the radargram, interpreted here as noise caused by the high level of iron and other minerals present throughout the deposit. The second major feature is the high amplitude feature located between 20m and 35m northeast of the initial survey start point, interpreted as possibly representing the now buried spring eye. This feature can also be seen on all the remaining radargrams that traverse this area.

Features observed in the 50MHz survey can be cross correlated within the 250MHz data, however the level of detail of the imagery from this antenna was lower resolution and no additional observations could be made. In addition, the depth to which the 50MHz antenna was able to penetrate was not significantly greater in these conditions. Although hyperbola fitting was conducted on the data generated from the surveys to determine the time depth of the radargrams, there are limitations on the reliability of the depth estimates. The variable nature of the spring deposits, along with hard iron panning overlying softer sediment, may have a significant impact on calculating the relative dielectric properties of the sediments and the correct velocity and vertical depth. Future improvements to increase the accuracy of the vertical resolution in these conditions could be to use the “rebar” test (see Conyers and Goodman 1997, Conyers and Lucius 1996), by driving a long piece of rebar into the deposit from a horizontal section at a set depth. The GPR is then run over this and the data is used to calibrate the velocities for that area. In addition, further work at the site will be conducted to ground-truth the main anomaly located in the radargrams.


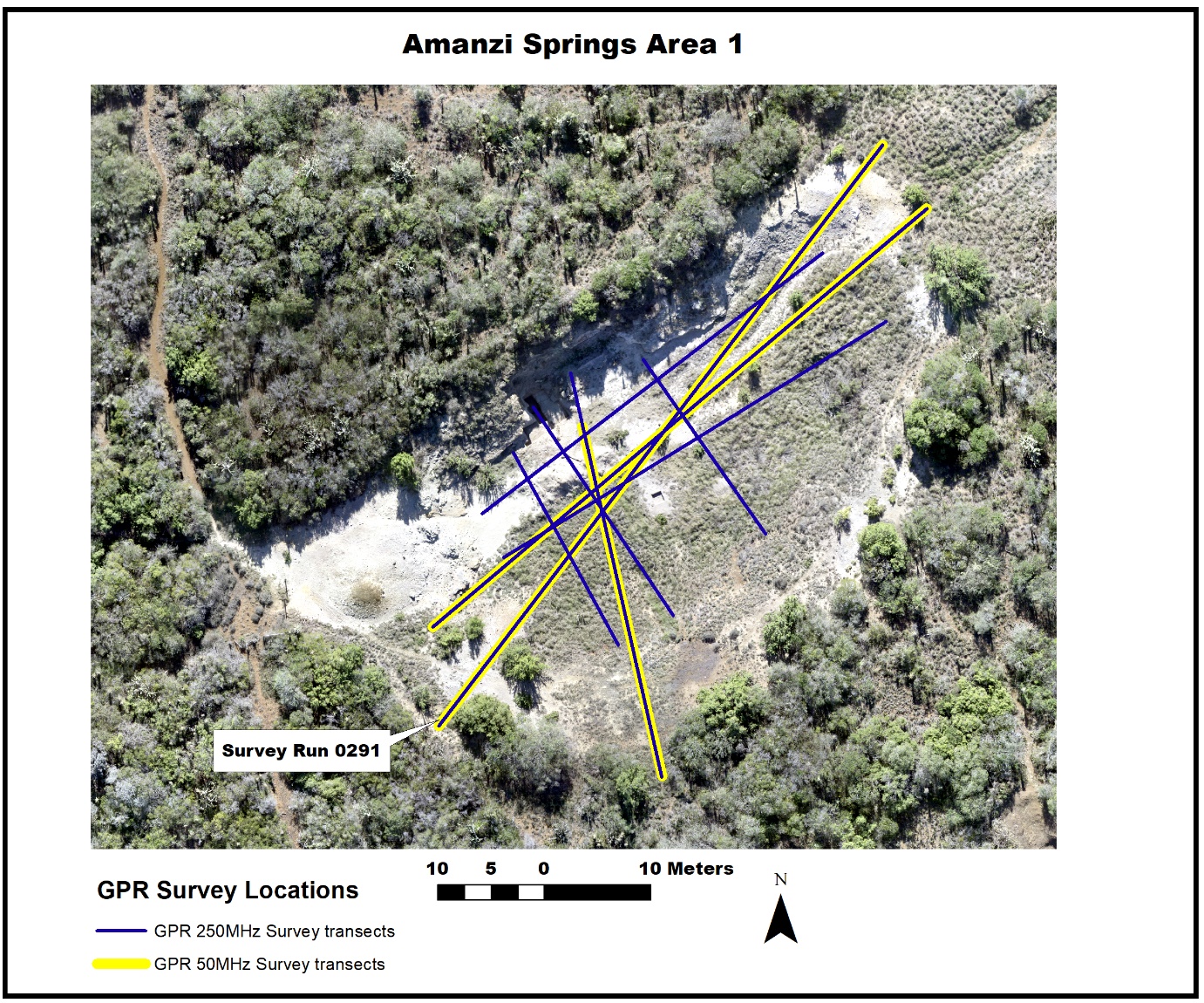


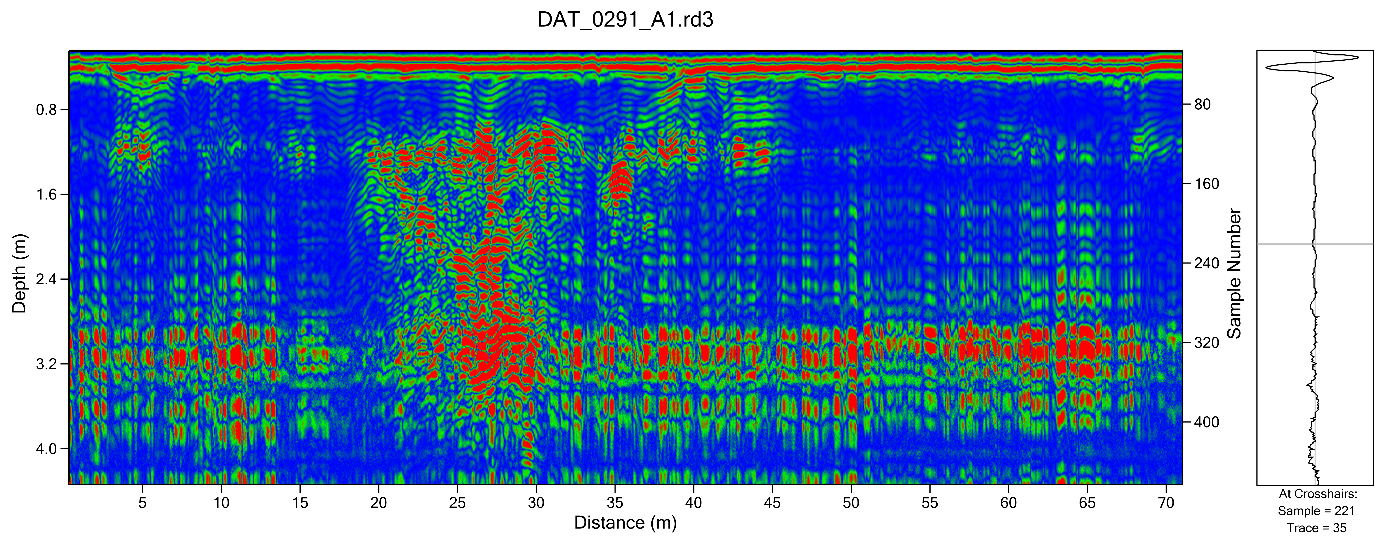


**S1.7 Fig** **Ground Penetrating Radar analysis.** **A**. Location of GPR Survey transects Amanzi Springs Area 1. Drone imagery taken in June 2017 after the dense vegetation covering the spring had been removed. **B**. Radargram 0291 that runs through the Area 1 Spring eye from south-west to north-east, note the vertical banding throughout and the high amplitude feature located between 20-35m.

**Analysis of iron mineralogy for palaeomagnetism**

**X-ray absorption near edge structure (XANES) methods**

Bulk sediment samples were homogenised with a mortar and pestle prior to being diluted in cellulose ([C_6_H_10_O_5_]_n_) at a 1:10 ratio for fluorescence mode x-ray absorption spectroscopy (XAS). The powder mixtures were then packed tightly into 2 mm thick perspex sample platens and sealed with kapton tape. Reference mineral standards were not diluted prior to their measurement and were loaded in either perspex or aluminium platens for transmission mode XAS.

XANES spectra were collected at the Fe *K*-edge (7122 eV) on the wiggler XAS 12-ID beamline (Glover et al., 2006) at the 3 GeV Australian Synchrotron (200 mA ring storage), Melbourne, Australia. The incident x-ray energy was controlled by a Si(311) double-crystal monochromator operated at the peak of the rocking curve in a fully tuned state. Higher harmonics were rejected with a Si-coated collimating mirror and a Rh-coated toroidal focusing mirror. The samples were scanned in a He-purged sample chamber at room temperature, with fluorescence mode spectra collected using a CANBERRA 100 element HP-Ge fluorescence detector. Transmission mode spectra were collected using OKEN ion chambers. An iron foil placed between the second and third ion chambers was measured simultaneously as a reference and for energy calibration. Radiation hardness tests were conducted at the beginning of the experiment.

For fluorescence scans, the spectra were pre-treated in Sakura (Kappen et al., 2015), with the remaining analysis undertaken in XANES dactyloscope 6.00 (Klementiev, 2012). The pre-edge background was subtracted by polynomial interpolation and the spectra were normalised to unity by the average post-edge. The maximum in the first derivative of the Fe reference foil was used for spectral energy calibration.

**Rock magnetism methods**

Rock magnetic experiments were undertaken at The Australian Archaeomagnetism Laboratory (TAAL), La Trobe University, Australia, with additional experiments conducted at the Institute for Rock Magnetism, University of Minnesota, USA. Low (χ_LF_; 0.465 kHz) and high (χ_HF_; 4.65 kHz) field magnetic susceptibility readings were taken using a Bartington MS2 magnetic susceptibility meter as a proxy for magnetic grain size and concentration. Isothermal remanent magnetisation (IRM) acquisition curves were measured using a MMPM10 Pulse Magnetiser. Hysteresis loops were measured at room temperature on a Princeton Measurements Vibrating Sample Magnetometer. Curie temperatures (*T*_c_) were estimated using the first derivative minima of heating and cooling curves derived from high temperature magnetic susceptibility (χ*T*) measurement sweeps taken from room temperature to 700°C using a Geofyzika KLY-2 KappaBridge AC Susceptibility Bridge. Other diagnostic mineralogical proxies were sought by observing changes in remanence at low temperature using a Quantum Designs Magnetic Properties Measurement System XL. Samples were subject to a ‘sweep-cool-warm’ experiment, whereby a room temperature (300 K) saturation (S) IRM of 2.5 T is imparted and remanence measurements are made while cooling to 20 K. At 20 K another SIRM of 2.5 T is given and the remanence is observed upon heating back to 300 K. Hysteresis, χ*T* and low temperature data were analysed using IRM-DB software, produced in-house at Institute for Rock Magnetism, and IRM curves were deconvoluted into separate coercivity components using MAX UnMix (Maxbauer et al., 2016).

**XANES results**

XANES spectra for both Area 1 samples are similar and indicative of Fe^3+^ iron oxyhydroxide phases (Fig. S8). Of the measured reference compounds (FeOOH, FeS, FeSO_4_, ferrihydrite, goethite, hemaetite, lepidocrocite, maghaemite and magnetite), the spring deposits best reflect lepidocrocite (Fig. S8a and 8b), which is paramagnetic at room temperature and cannot carry a palaeomagnetic signal. Lepidocrocite is best supported in the pre-edge region over other oxyhydroxides (goethite, ferrihydrite and FeOOH) by the low intensity and multi-component peak of the sample spectra (Fig. S8c). We thus interpret lepidocrocite to dominate the overall Fe mineral content, perhaps with other iron oxyhydroxides, alongside other minor Fe constitutes including those that are remanence-bearing. A linear combination fit was not attempted with other reference compounds given a lack of certainty in what other phases may be present. Of the potential minor Fe constitutes, the typical iron oxide remanence carriers magnetite and maghaemite could only exist in trace quantities as the pronounced energy of their pre-edge peaks would dominate this region (Fig. S8c). The iron sulphide remanence carriers pyrrhotite (Fe_[₁₋ₓ]_S) and greigite (Fe_3_S_4_) could only exist in even smaller concentrations based on the distinct lower energy XANES features of iron sulphides, with examples here from FeS and FeSO_4_ (Fig. S8a).

**Rock magnetism results**

χ_LF_ values were low at 0.057–0.023 ×10-6 m^3^/kg^-1^ suggesting only minor quantities of strong ferrimagnetic minerals, or a predominance of phases that are low in χ. There is limited evidence for the presence of magnetite in RPM-1 via a subtle inflection at 119–224 K close to the magnetite Verwey transition observed during low temperature 2.5T SIRM cooling (Fig. S9d). This feature is not observed in RPM-2, indicating either that magnetite is not present in this sample, or that the Verwey transition has been suppressed or eliminated by grain impurities (Moskowitz et al., 1998; Özdemir et al., 1993a; Özdemir & Dunlop, 2010). The removal of a stable remanence at ~220°C could relate to a reduced unblocking temperature in titanomagnetite, although this is considered less likely as there is no corresponding *T*_c_ registered on χ*T* curves (Fig. S9c). The presence of magnetite or maghaemite could not be corroborated by their *T*_c_ (~580–620°C) as new impure magnetite (*T*_c_ of 550–566°C) was produced on heating above ~400°C in air (Fig. S9c). This alteration was also apparent on TH demagnetisation with a chemical remanent magnetisation (CRM) formed above 400–500°C (Fig. S7c). This transformation likely occurred from a non-remanence bearing paramagnetic phase, several of which transform to magnetite at temperature. Strong paramagnetic signatures are seen in hysteresis loops (Fig. S9b), which could be caused by high concentrations of lepidocrocite.

Regarding the low temperature remanence removal during TH demagnetisation (Fig. S7c), this was also considered as potentially caused by unstable magnetite grain sizes (i.e. superparamagnetic or multi-domain). However, the dominant intermediate coercivities (74–82 mT) estimated by IRM unmixing (Fig. S9a), which reflect the ChRM isolated by AF demagnetisation (Fig. S7c), do not support this. Rather, these coercivities are consistent with what is commonly reported for pyrrhotite or greigite (Weaver et al., 2002). These iron sulphides are much lower in χ (Peters & Dekkers, 2003) and unblock at temperatures of ~230–350°C (Dekkers, 1989; Roberts et al., 2011) like that represented in the TH demagnetisation spectra (Fig. S7c). Given their overlap in magnetic properties distinguishing between either mineral based on present data is difficult (Horng & Roberts, 2006), and we note the lack of other diagnostic properties such as a gyroremanent magnetisation in greigite (Fig. S7b and S7c) and the ~34 K Benus transition in (monoclinic) pyrrhotite (Fig. S9d). Greigite can be problematic from a palaeomagnetic perspective as it is often produced during late diagenesis and can acquire a CRM in the process, thus determining the timing of its growth is crucial (Roberts & Weaver, 2005). Pyrrhotite has been identified as both a primary detrital (e.g. Horng & Roberts, 2006) and secondary authigenic (e.g. Weaver et al., 2002) mineral in sediments and thus remanences may be depositional (primary) or chemical (secondary) in origin. While the presence of iron sulphides is not indicated by the XANES analysis, we suggest that either pyrrhotite or greigite best explains the remanence properties (over magnetite, maghaemite or titanomagnetite, for which evidence is limited) as a trace component alongside the dominant iron oxyhydroxides, namely lepidocrocite.

A final point of note is that IRM experiments show the presence of a small contribution high coercivity component relating to hematite, or more likely goethite (Fig. S9a). The AF and TH demagnetisation spectra indicate that neither mineral contributes to the ChRM. The mineralogy of the Amanzi springs deposits is complex and as such whether the remanence is primary or secondary cannot be established with confidence, although the normal polarity is consistent with the ages based on other methods.


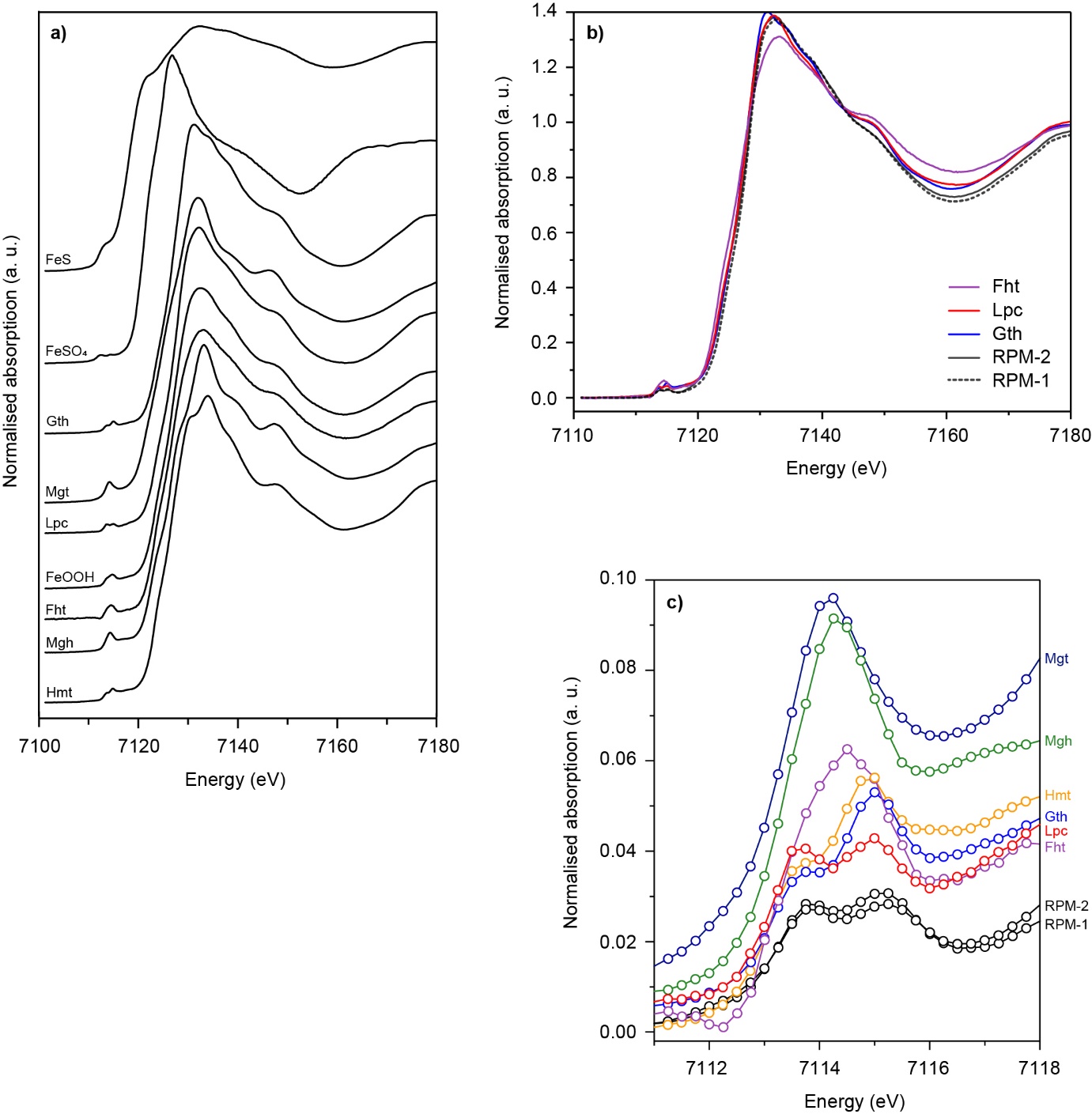


**S1.8 Fig XAS spectra for Amanzi Springs Area 1**. A) Stacked XANES spectra for reference compounds (Gth = goethite, Mgt = magnetite, Lpc = lepidocrocite, Fht = ferrihydrite, Mgh = maghaemite, Hmt = haematite). B) XANES spectra for sample spectra RPM-1 and RPM-2 alongside naturally occurring iron oxyhydroxides. C) Close up on the pre-edge XANES region showing the sample spectra and selected reference compounds.


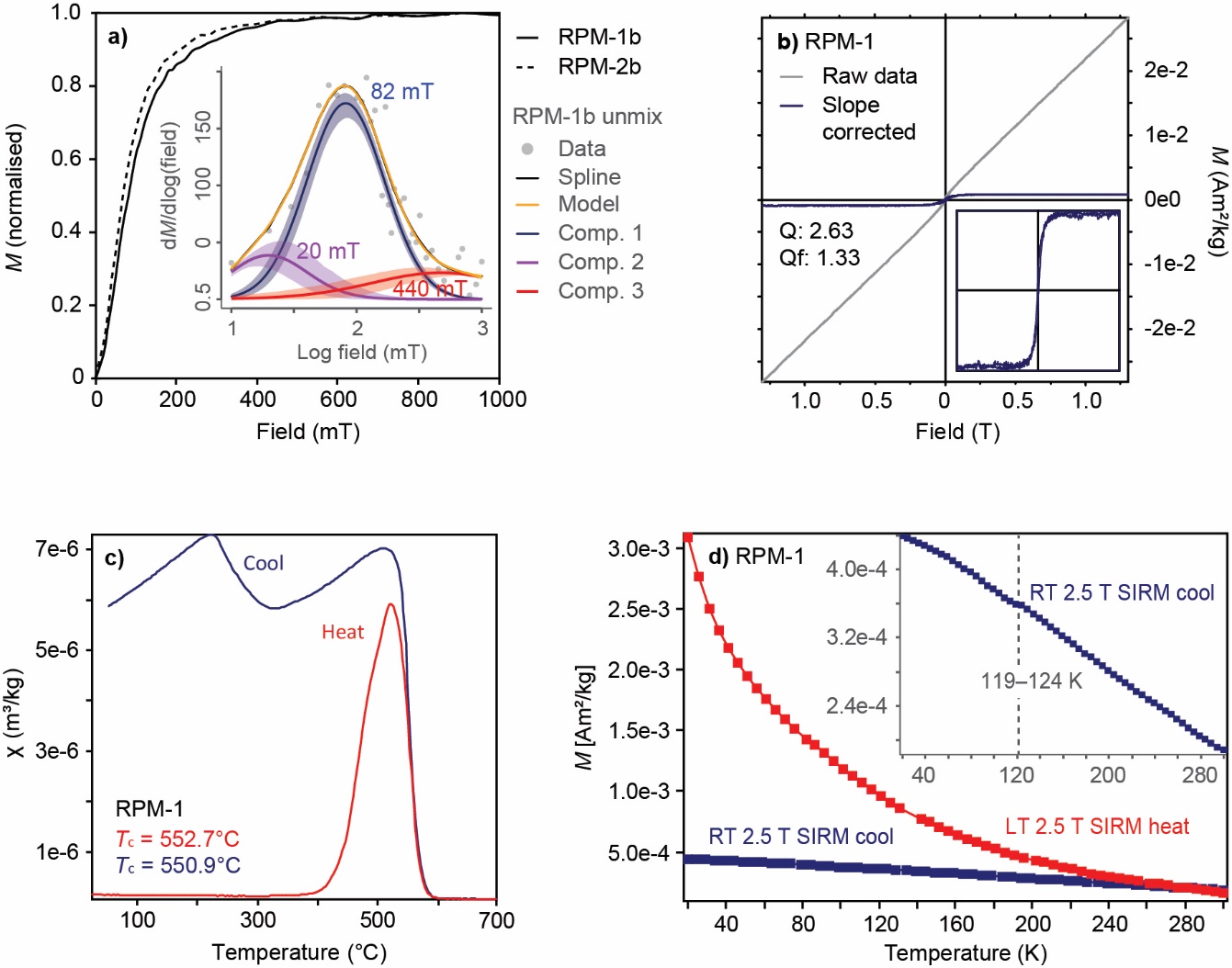


**S1.9 Fig** **Representative rock magnetic data for Amanzi Springs Area 1.** A) IRM acquisition curves with a component unmix insert. B) Hysteresis loop with an insert showing slope corrected data. C) χ*T* heating and cooling curves. D) Low temperature sweep-cool-warm cooling and heating curves (RT = room temperature, LT = low temperature).

| Sample | *n* | Dec (°) | Inc (°) | Mean MAD (°) | α95 (°) | *K* | VGP lat (°) | Polarity |
| --- | --- | --- | --- | --- | --- | --- | --- | --- |
| RPM-1 | 8/9 | 330.0 | -55.7 | 7.95 (σ1.66) | 8.78 | 40.77 | 65.39 | N |
| RPM-2 | 4/6 | 310.7 | -53.32 | 7.75 (σ1.17) | 20.12 | 21.82 | 49.43 | N |

**S1.4 Table. Mean palaeomagnetic directions for Amanzi Springs Area 1.**

______________________________________________________________________

AMZ code        Lab Code      Material F14C%          Result

____________________________________________________________________________

AMZ1-259    Wk-52004 *  charcoal 0.1 +/- 0.0   > 53000 (indistinguishable from background)

AMZ1-264    Wk-52005 *  wood  0.1 +/- 0.0 > 53000 (indistinguishable from background)

* The Carbon-13 stable isotope value (delta 13C) was measured on prepared graphite using the AMS spectrometer. The radiocarbon date has therefore been corrected for isotopic fractionation. However the AMS-measured delta 13C value can differ from the delta 13C of the original material and it is therefore not shown.

**S1.5 Table. Radiocarbon dates for wood and charcoal from Amanzi Springs Area 1.**

|  |  |  |  |  |  |  |
| --- | --- | --- | --- | --- | --- | --- |

**References**

Aitken, M.J., 1985. Thermoluminescence Dating. Academic Press, London, 359 p.

Aitken, M.J., 1998. An Introduction to Optical Dating: The Dating of Quaternary Sediments by the Use of Photon-Stimulated Luminescence. Oxford University Press, Oxford, 267 p.

Alappat, L., Tsukamoto, S., Singh, P., Srikanth, D., Ramesh, R., Frechen, M., 2010. Chronology of Cauvery Delta sediments from shallow subsurface cores using elevated-temperature post-IR IRSL dating of feldspar. Geochronometria 37, 37-47.

Arnold, L.J., Roberts, R.G., MacPhee, R.D.E., Haile, J.S., Brock, F., Möller, P., Froese, D.G., Tikhonov, A.N. Chivas, A.R., Gilbert, M.T.P., Willerslev, E. 2011. Paper II – Dirt, dates and DNA: OSL and radiocarbon chronologies of perennially-frozen sediments and their implications for sedimentary ancient DNA studies. Boreas 40, 417-445.

Arnold, L.J., Duval, M., Falguères, C., Bahain, J.-J., Demuro, M., 2012a. Portable gamma spectrometry with cerium-doped lanthanum bromide scintillators: Suitability assessments for luminescence and electron spin resonance dating applications. Radiation Measurements 47, 6–18.

Arnold, L.J., Demuro, M., Navazo Ruiz, M., 2012a. Empirical insights into multi-grain averaging effects from ‘pseudo’ single-grain OSL measurements. Radiation Measurements 47, 652-658.

Arnold, L.J., Demuro, M., Navazo Ruiz, M., Benito-Calvo, A., Perez-Gonzalez, A., 2013. OSL dating of the Middle Palaeolithic Hotel California site, Sierra de Atapuerca, north-central Spain. Boreas 42, 285-305.

Arnold, L.J., Demuro, M., Parés, J.M., Arsuaga, J.L., Aranburu, A., Bermúdez de Castro, J.M., Carbonell, E. 2014. Luminescence dating and palaeomagnetic age constraint on hominins from Sima de los Huesos, Atapuerca, Spain. Journal of Human Evolution 67, 85-107.

Arnold, L.J., Duval, M., Demuro, M., Spooner, N.A., Santonja, M., Pérez-González, A., 2016. OSL dating of individual quartz 'supergrains' from the Ancient Middle Palaeolithic site of Cuesta de la Bajada, Spain. Quaternary Geochronology 36, 78-101.

Arnold, L.J., Demuro, M., Spooner, N.A., Prideaux, G.J., McDowell, M.C., Camens, A.B., Reed, E.H., Parés, J.M., Arsuaga, J.L., Bermúdez de Castro, J.M., Carbonell, E., 2019. Single-grain TT-OSL bleaching characteristics: Insights from modern analogues and OSL dating comparisons. Quaternary Geochronology, 49, 45-51.

Banerjee, D., Murray, A.S., Bøtter-Jensen, L., Lang, A., 2001. Equivalent dose estimation using a single aliquot of polymineral fine grains. Radiation Measurements 33, 73-94.

Bartz, M., Arnold, L.J., Demuro, M., Duval, M., King, G.E., Rixhon, G., Álvarez Posada, C., Parés, J.M., Brückner, H., 2019. Single-grain TT-OSL dating results confirm an Early Pleistocene age for the lower Moulouya River deposits (NE Morocco) Quaternary Geochronology 49, 254-261.

Berger, G.W., Pérez-González, A., Carbonell, E., Arsuaga, J.L., Bermúdez de Castro, J.-M., Ku, T.-L., 2008. Luminescence chronology of cave sediments at the Atapuerca paleoanthropological site, Spain. Journal of Human Evolution 55, 300–311.

Bøtter-Jensen, L., Mejdahl, M., 1988. Assessment of beta dose-rate using a GM multicounter system. Nuclear Tracks and Radiation Measurements 14, 187-191.

Bowler, J.M., Johnston, H., Olley, J.M., Prescott, J.R., Roberts, R.G., Shawcross, W., Spooner, N.A., 2003. New ages for human occupation and climate change at Lake Mungo, Australia. Nature 421, 837–840.

Brennan, B.J., 2003. Beta doses to spherical grains. Radiation Measurements 37, 299-303.

Brown, N.D., Forman, S.L., 2012. Evaluating a SAR TT-OSL protocol for dating fine grained quartz within Late Pleistocene loess deposits in the Missouri and Mississippi river valleys, United States. Quaternary Geochronology 12, 87-97.

Buylaert, J.P., Thiel, C., Murray, A.S., Vandenberghe, D.A.G., Yi, S., Lu, H., 2011. IRSL and post-IR IRSL residual doses recorded in modern dust samples from the Chinese loess plateau. Geochronometria 38, 432-440.

Channell, J. E. T., B. S. Singer and B. R. Jicha 2020 Timing of Quaternary geomagnetic reversals and excursions in volcanic and sedimentary archives. Quaternary Science Reviews 228:1–29.

Conyers, L.B. and Goodman, D., 1997. *Ground-Penetrating Radar*. Lanham: AltaMira Press.

Conyers, L.B. and Lucius, J.E., 1996. Velocity analysis in archaeological ground-penetrating radar studies. *Archaeological Prospection*, 3 (1), pp. 25–38.

Dekkers, M. J. 1989 Magnetic properties of natural pyrrhotite. II. High- and low-temperature behavior of Jrs and TRM as a function of grain size. Physics of the Earth and Planetary Interiors 57:266–283.

Demuro, M., Arnold, L.J., Froese, D.G., Roberts, R.G., 2013. OSL dating of loess deposits bracketing Sheep Creek tephra beds, northwest Canada: dim and problematic single-grain OSL characteristics and their effect on multi-grain age estimates. Quaternary Geochronology 15, 67-87.

Demuro, M., Arnold, L.J., Pares, J.M., Sala, R., 2015. Extended-range luminescence chronologies suggest potentially complex bone accumulation histories at the Early-to-Middle Pleistocene palaeontological site of Huescar-1 (Guadix-Baza basin, Spain). Quaternary International 389, 191-212.

Demuro, M., Arnold, L.J., Parés, J.M., Sala, R., 2015. Extended-range luminescence chronologies suggest potentially complex bone accumulation histories at the Early-to-Middle Pleistocene palaeontological site of Huéscar-1 (Guadix-Baza basin, Spain). Quaternary International 389, 191-212.

Demuro, M., Arnold, L.J., Aranburu, A., Sala, N., Arsuaga, J.-L., 2019. New bracketing luminescence ages constrain the Sima de los Huesos hominin fossils (Atapuerca, Spain) to MIS 12. Journal of Human Evolution 131, 76-95.

Duller, G.A.T., 2003. Distinguishing quartz and feldspar in single grain luminescence measurements. Radiation Measurements 37, 161–165.

Duller, G.A.T., 2007. Assessing the error on equivalent dose estimates derived from single aliquot regenerative dose measurements. Ancient TL 25, 15-24.

Durcan, J.A., Duller, G.A.T., 2011. The fast ratio: a rapid measure for testing the dominance of the fast component in the initial OSL signal from quartz. Radiat. Meas. 46, 1065–1072.

Duval, M., Arnold, L.J. 2013. Field gamma dose-rate assessment in natural sedimentary contexts using LaBr3(Ce) and NaI(Tl) probes: A comparison between the "threshold" and "windows" techniques. Applied Radiation and Isotopes 74, 36-45.

Feathers, J.K., Casson, M. A., Schmidt, A.H., Chithamboet, M.L., 2012. Application of pulsed OSL to polymineral fine-grained samples. Radiation Measurements 47, 201-209.

Fisher, R. 1953 Dispersion on a sphere. Proceedings of the Royal Society of London. Series A, Mathematical and Physical Sciences 217:512–515.

Galbraith, R.F., 2002. A note on the variance of a background-corrected OSL count. Ancient TL 20, 49-51.

Glover, C., J. Mckinlay, M. Clift, B. Barg, J. Boldeman, M. Ridgeway, G. Foran, R. Garrett, P. Lay and A. Broadbent 2006 Status of the x-ray absorption spectroscopy (XAS) beamline at the Australian Synchrotron. AIP Conference Proceedings 882(1):884–886.

Guérin, G., Mercier, M., Adamiec, G., 2011. Dose-rate conversion factors: update. Ancient TL 29, 5-8.

Hansen, V., Murray, A., Buylaert, J.-P., Yeo, E.-Y., Thomsen, K., 2015. A new irradiated quartz for beta source calibration. Radiation Measurements 81, 123-127.

Heslop, D. and A. P. Roberts 2016 Analyzing paleomagnetic data: to anchor or not to anchor? Journal of Geophysical Research: Solid Earth 121(11):7742–7753.

Horng, C-S and A. P. Roberts 2006 Authigenic or detrital origin of pyrrhotite in sediments?: Resolving a paleomagnetic conundrum. Earth and Planetary Science Letters 241:750–762.

Huntley, D.J., Baril, M.R., 1997. The K content of the K-feldspars being measured in optical dating or in thermoluminescence dating. Ancient TL 15, 11-13.

Huntley, D.J., Clague, J.J., 1996. Optical dating of tsunami-laid sands. Quaternary Research 46, 127-140.

Huntley, D.J., Lian, O.B., 1999. Using optical dating to determine when a sediment was last exposed to sunlight. In D.S. Lemmen and R.E. Vance (eds.), Holocene Climate and Environmental Change in the Palliser Triangle: A Geoscientific Context for Evaluating the Impacts of Climate Change on the Southern Canadian Prairies. Geological Survey of Canada, Ottawa, Bulletin 534, 211-222.

Huntley, D.J., Hancock, R.G.V., 2001. The Rb contents of the K-feldspar grains being measured in optical dating. Ancient TL 19, 43-46.

Huntley, D.J., Lamothe, M., 2001. Ubiquity of anomalous fading in K-feldspars and the measurement and correction for it in optical dating. Canadian Journal of Earth Science 38, 1093-1106.

Jacobs, Z., Duller, G.A.T., Wintle, A.G., 2006a. Interpretation of single-grain D_e_ distributions and calculation of D_e_. Radiation Measurements 41, 264–277.

Jacobs, Z., Duller, G.A.T., Wintle, A.G., Henshilwood, C.S., 2006b Extending the chronology of deposits at Blombos Cave, South Africa, back to 140 ka using optical dating of single and multiple grains of quartz. Journal of Human Evolution 51, 255-273.

Jacobs, Z., Wintle, A.G., Roberts, R.G., Duller, G.A.T., 2008. Equivalent dose distributions from single grains of quartz at Sibudu, South Africa: context, causes and consequences for optical dating of archaeological deposits. Journal of Archaeological Science 35, 1808-1820.

Kappen, P., G. Ruben, and A. Moll 2015 Sakura: a tool to pre-process fluorescence XAS data from multi-pixel detectors <http://archive.synchrotron.org.au/aussyncbeamlines/x-ray-absorption-spectroscopy/sakura>

Kirschvink, J. L. 1980 The least-square line and plane and the analysis of palaeomagnetic data. Geophysical Journal, Royal Astronomical Society 62:699–718.

Klementiev, K. V. 2012 XANES dactyloscope for Windows. Freeware: www.cells.es/Beamlines/CLAESS/software/xanda.html

Koymans, M. R., C. G. Lanereis, D. Pastor-Galan and D. J. J. van Hinsbergen 2016 Paleomagnetism.org: an online multi-platform open source environment for paleomagnetic data analysis. Computers and Geosciences 93:127–137.

Lang, A., Wagner, G. A., 1997. Infrared stimulated luminescence dating of Holocene colluvial sediments using the 410 nm emission. Quaternary Science Reviews 16, 393–396.

Lang, A., Hatté, C., Rousseau, D. D., Antoine, P., Fontugne, M., Zöller, L., Hambach, U., 2003. High-resolution chronologies for loess: comparing AMS 14C and optical dating results. Quaternary Science Reviews 22, 953–959.

Lewis, R., Tibby, J., Arnold, L.J., Barr, C., Marshall, J., McGregor, G., Gadd, P., Yokoyama, Y., 2020. Insights into subtropical Australian aridity from Welsby Lagoon, North Stradbroke Island, over the past 80,000 years. Quaternary Science reviews 234, 106262.

Lurcock, P. C. and G. S. Wilson 2012 PuffinPlot: a versatile, user-friendly program for paleomagnetic analysis. Geochemistry, Geophysics, Geosystems 13(6):1–6.

Maxbauer, D. P., J. M. Feinberg and D. L Fox 2016 MAX UnMix: a web application for unmixing magnetic coercivity distributions. Computers and Geosciences 95:140–145.

Mejdahl, V., 1979. Thermoluminescence dating: beta-dose attenuation in quartz grains. Archaeometry 21, 61–72.

Mejdahl, V., 1987. Internal radioactivity in quartz and feldspar grains. Ancient TL 5, 10–17.

Méndez-Quintas, E., Santonja, M., Pérez-González, A., Duval, M., Demuro, M., Arnold, L.J., 2018. First evidence of an extensive Acheulean large cutting tool accumulation in Europe from Porto Maior (Galicia, Spain). Nature Scientific Reports 8, 3082.

Moskowitz, B. M., M. Jackson and C. Kissel 1998 Low temperature magnetic behaviour of titanomagnetites. Earth and Planetary Science Letters 157:141–149.

Ollé, A., Vergès, J.M., Rodríguez, X.P., Cáceres, I., Angelucci, D.E., Vallverdú, J., Demuro, M., Arnold, L.J., Falguères, C., Bennàsar, M., López-García, J.M., Blain, H-.A., Bañuls-Cardona, S., Burjachs, F., Expósito, I., López-Polín1, L., López-Ortega, E., 2016. The Middle Pleistocene site of La Cansaladeta (Tarragona, Spain): Stratigraphic and archaeological succession. Quaternary International 393, 137-157.

Özdemir, Ö. and D. J. Dunlop 2010 Hallmarks of maghemization in low-temperature remanence cycling of partially oxidized magnetite nanoparticles. Journal of Geophysical Research 115:1–10.

Özdemir, Ö., D. Dunlop and B. M. Moskowitz 1993 The effect of oxidation on the Verway transition in magnetite. Geophysical Research Letters 20:1671–1674.

Pawley, S.M., Bailey, R.M., Rose, J., Moorlock, B.S.P., Hamblin, R.J.O., Booth, S.J., Lee, J.R., 2008. Age limits on Middle Pleistocene glacial sediments from OSL dating, north Norfolk, UK. Quaternary Science Reviews 27, 1363-1377.

Peters, C. and M. J. Dekkers 2003 Selected room temperature magnetic parameters as a function of mineralogy, concentration and grain size. Physics and Chemistry of the Earth, Parts A/B/C 28(16–19):659–657.

Potts, P.J., Thompson, M., Chenery, S.R.N., Webb, P.C., Kasper, H.U., 2003. Geopt13 - An International Proficiency Test for Analytical Geochemistry Laboratories - Report on Round 13 / July 2003 (Köln Loess). International Association of Geoanalysts.

Prescott, J.R., Hutton, J.T., 1994. Cosmic ray contributions to dose rates for luminescence and ESR dating: large depths and long-term time variations. Radiation Measurements 23, 497–500.

Qin, J.T., Zhou, L.P., 2012. Effects of thermally transferred signals in the post-IR IRSL SAR protocol. Radiation Measurements 47, 710-715.

Readhead, M.L., 1987. Thermoluminescence dose rate data and dating equations for the case of disequilibrium in the decay series. Nuclear Tracks and Radiation Measurements 13, 197-207.

Readhead, M.L., 2002. Absorbed dose fraction for ^87^Rb β particles. Ancient TL 20, 25–28.

Rees-Jones, J., 1995. Optical dating of young sediments using fine-grain quartz. Ancient TL 13, 9-14.

Rees-Jones, J., Tite, M.S., 1997. Optical dating results for British archaeological sediments. Archaeometry 39, 177-187.

Roberts, A. P. and R. Weaver 2005 Multiple mechanisms of remagnetization involving sedimentary greigite (Fe3S4). Earth and Planetary Science Letters 231:263–277.

Roberts, A. P., L. Chang, C. J. Rowan, C-S. Horng and F. Florindo 2011 Magnetic properties of sedimentary greigite (Fe3S4): An update. Reviews of Geophysics 49(1):1–46.

Russell, N.J., Armitage, S.J., 2012. A comparison of single-grain and small aliquot dating of fine sand from Cyrenaica, northern Libya. Quaternary Geochronology 10, 62-67.

Stone, A.E.C., Bailey, R.M., 2012. The effect of single grain luminescence characteristics on single-aliquot equivalent dose estimates. Quaternary Geochronology 11, 68-78.

Torsvik, T. H., R. Van der Voo, U. Preeden, C. M. Niocaill, B. Steinberger, P. V. Doubrovine, D. J. J. van Hunsbergen, M. Domeier, C. Gaina, E. Tohver, J. G. Meert, P. J. A. McCausland and L. R. M. Cocks 2012 Phanerozoic polar wander, palaeogeography and dynamics. Earth-Science Reviews 114:325–368.

Tsukamoto, S., Duller, G.A.T., Wintle, A.G., 2008. Characteristics of thermally transferred optically stimulated luminescence (TT-OSL) in quartz and its potential for dating sediments. Radiation Measurements 43, 1204-1218.

Wang, X.L., Wintle, A.G., 2013. Investigating the contribution of recuperated TL to post-IR IRSL signals in a perthitic feldspar. Radiation Measurements 49, 82-87.

Weaver, R., A. P. Roberts and A. J. Barker 2002 A late diagenetic (syn-folding) magnetization carried by pyrrhotite: implications for paleomagnetic studies from magnetic iron sulphide-bearing sediments. Earth and Planetary Science Letters 200(3–4):371–386

Wintle, A.G., Murray, A.S., 2006. A review of quartz optically stimulated luminescence characteristics and their relevance in single-aliquot regeneration dating protocols. Radiation Measurements 41, 369-391.

Yoshida, H., Roberts, R.G., Olley, J.M., Laslett, G.M., Galbraith, R.F., 2000. Extending the age range of optical dating using single ‘supergrains’ of quartz. Radiation Measurements 32, 439-44
